# A proper excitatory/inhibitory ratio is required to develop synchronized network activity in mouse cortical cultures

**DOI:** 10.1016/j.stemcr.2025.102646

**Published:** 2025-09-25

**Authors:** Eleonora Crocco, Ludovico Iannello, Fabrizio Tonelli, Gabriele Lagani, Luca Pandolfini, Marcello Ferro, Giuseppe Amato, Angelo Di Garbo, Federico Cremisi

**Affiliations:** 1 Laboratorio di Biologia Bio@SNS, Scuola Normale Superiore, Pisa, Italy; 2 Institute of Information Science and Technologies (ISTI-CNR), Pisa, Italy; 3 Center for Human Technologies, Central RNA Lab, Istituto Italiano di Tecnologia, 16152 Genova, Italy; 4 Istituto di Linguistica Computazionale “Antonio Zampolli” (CNR-ILC), Pisa, Italy; 5 Istituto di Biofisica, Consiglio Nazionale delle Ricerche, 56124 Pisa, Italy; 6 Dipartimento di Fisica, Università di Pisa, 56127 Pisa, Italy

**Keywords:** spontaneous activity, excitatory/inhibitory balance, cortical development neuronal network activity, HD-MEAs, parvalbumin -positive neurons, functional connectivity, PV

## Abstract

Excitatory/inhibitory (E/I) balance is thought to play a key role in cortical activity development. We modeled an *in vitro* cortical network deployed of the inhibitory neurons normally migrating from the ventral telencephalon and implemented ventral telencephalic (VT) cultures and co-cultures with mixed proportions of dorsal telencephalic (DT) and VT neurons, containing distinct proportions of inhibitory neurons. Interestingly, these pure and mixed cultures developed different patterns of spontaneous activity and functional connectivity. Our findings highlighted a critical role for the inhibitory component in developing correlated network activity. Unexpectedly, networks with 7% of parvalbumin (PV)^+^ neurons were not able to generate appreciable network burst activity due to the development of a strong network inhibition, despite their lowest E/I ratio. Our observations support the notion that an optimal ratio of PV^+^ neurons during cortical development is essential for the establishment of local inhibitory networks capable of generating and spreading correlated activity.

## Highlights

• Cortical activity development relies on an optimal excitatory/inhibitory neuron balance
• *In vitro* modeled cortical networks reveal varied spontaneous activity patterns
• Inhibitory neurons critically influence bursting activity development
• Intermediate levels of E/I balance are required for network synchronized activity

## Graphical abstract

**Figure:**
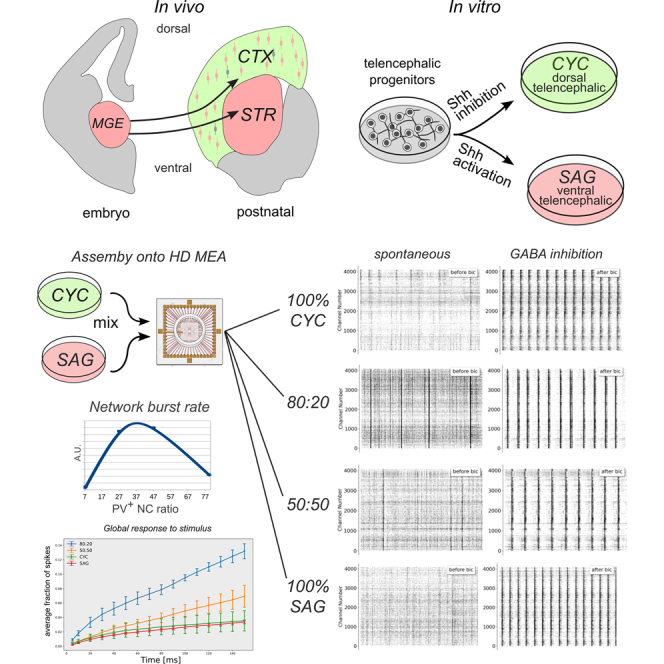

## Teaser

This research explores how the balance of excitatory and inhibitory neurons is critical for proper cortical activity development. We demonstrate that varying proportions of inhibitory neurons, particularly PV^+^ neurons, profoundly influence network activity patterns and functional connectivity. Our findings highlight the necessity of an optimal inhibitory balance for generating and spreading network correlated activity, revealing how even a slight imbalance can significantly disrupt network function.

## Introduction

Mouse embryonic stem cells (mESCs) or human induced pluripotent cells (hiPSCs) are key tools for modeling the development of distinct encephalic regions *in vitro* by controlling different signaling pathways in precise time windows (Chambers et al., 2009; Chiaradia and Lancaster, 2020). Forebrain identity is acquired by default and retained primarily through BMP and Wnt inhibition (Bertacchi et al., 2015; Watanabe et al., 2005). Dorsal telencephalic (DT) progenitors are generated *in vitro* using the sonic Hedgehog (Shh) inhibitor, cyclopamine (Gaspard et al., 2008), while ventral telencephalic (VT) progenitors require Shh activation by the Shh agonist, SAG (Cederquist et al., 2019; Li et al., 2009). *In vivo*, the mature cerebral cortex forms after VT cells migrate, mainly from the medial ganglionic eminence (MGE) (Wonders and Anderson, 2006). These cells differentiate into GABAergic inhibitory interneurons that connect with glutamatergic excitatory neurons generated by local DT progenitors, establishing a balanced Excitatory/Inhibitory(E/I) ratio (Gelman and Marín, 2010; Lodato et al., 2011). Notably, an unbalanced E/I ratio is linked to brain disorders such as schizophrenia or autism spectrum disorders (Nelson and Valakh, 2015; Sohal and Rubenstein, 2019).

To date, relatively few studies have reconstructed cortical circuits using defined ratios of VT and DT neurons (Mossink et al., 2022; Parodi et al., 2023, 2024). These studies used induced neurons (iNeurons) that only partially mimic natural telencephalic neurons. Specifically, *Ngn2*-induced neurons comprise a heterogeneous population displaying features of both central and peripheral nervous system lineages (Lin et al., 2021), while *Ascl1*-induced neurons show limited differentiation into parvalbumin-positive interneurons, which represent the predominant class of cortical inhibitory neurons *in vivo* (Zhang et al., 2013). To investigate more physiological neural networks with different E/I ratios and functionally characterize them during developmental maturation, we modeled two distinct populations of cells *in vitro*, DT and VT progenitors obtained by modulating the Shh pathway in mESC-derived telencephalic cells. We analyzed the activity of a network of DT progenitors alone, comparing it to a network of VT progenitors and to networks with varying ratios of DT and VT cells. Finally, we analyzed basic structural and functional network parameters such as synapse density, firing activity, network burst synchronization and connectivity, together with the ability to respond to electrical stimulations. Our findings indicate that the E/I balance dramatically affects the development and maturation of neuronal cultures, highlighting that a proper E/I ratio is required for the formation and spreading of correlated activity in cortical networks.

## Results

### Cyclopamine and SAG treatments respectively induce the production of DT and VT neurons

**Figure 1:**
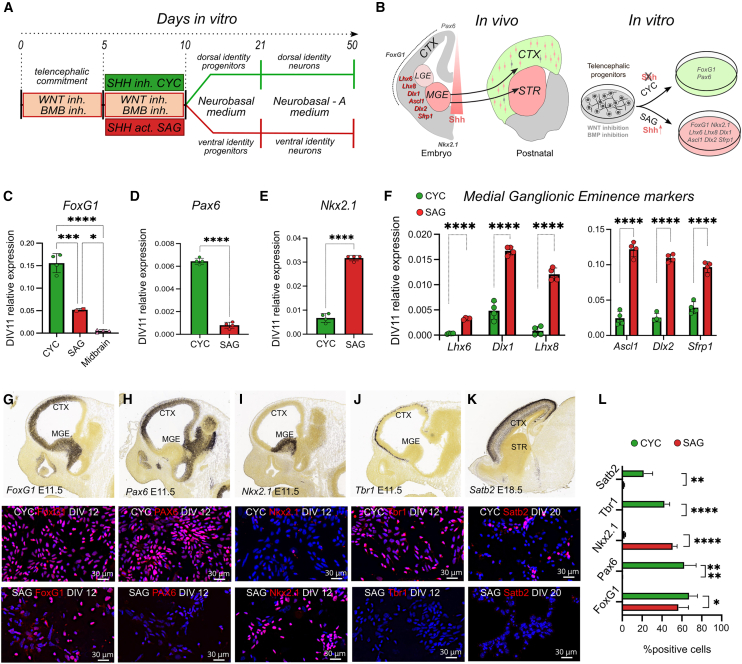
Positional identity and characterization of CYC and SAG progenitor cells (A) Protocol of mESC neuralization. (B) Schematic representation of marker expression *in vivo* and *in vitro*. CTX, cortex; STR, striatum; MGE, medial ganglionic eminence; LGE, lateral ganglionic eminence. (C–E) Relative expression of early pallial markers by quantitative reverse-transcription PCR (RT-qPCR) (*n* = 4 independent experiments). Midbrain in (C): cultures without WiBi-induced neuralization with mesencephalic identity (Bertacchi et al., 2015). Mean ± SD is shown; ordinary one-way ANOVA with Tukey’s multiple comparisons test was performed for *Foxg1* expression; unpaired t test was performed for *Pax6* and *Nkx2.1* markers. (F) Relative expression of MGE-LGE markers by RT-qPCR (*n* = 4 independent experiments). Mean ± SD is shown; multiple unpaired t test with Holm-Šídák correction method. *p* values: ^∗^*p* value < 0.05, ^∗∗∗^*p* value < 0.001, ^∗∗∗∗^*p* value < 0.0001. (G–L) Bottom: immunolabeling and quantification of CYC and SAG cultures for the indicated markers at the *in vitro* differentiation day (DIV) indicated in labels (blue nuclear counterstaining by DAPI). Scale bar, 30 μm. The *in situ* hybridization (ISH) insets at the figure top, showing the *in vivo* expression of the markers for comparison, are taken from Allen Brain Atlas: Developing Mouse Brain (https://developingmouse.brain-map.org/). In (L), mean ± SD and multiple unpaired t test with Holm-Šídák correction method are shown: ^∗∗^*p* value < 0.01, ^∗∗∗^*p* value < 0.001, ^∗∗∗∗^*p* value < 0.0001.

During the first week of mESC neuralization, early double inhibition of Bone Morphogenetic Protein (BMP) and Wnt signaling (WiBi) induces a general telencephalic identity (Terrigno et al., 2018) (Figures 1A and 1B). From *in vitro* differentiation day (DIV)5 to DIV10, we added cyclopamine (3 μM) to WiBi-treated mESC to induce DT identity (Chen et al., 2002a) and smoothened agonist (SAG) (0.1 μM) to induce VT identity (Chen et al., 2002b). At DIV11, we confirmed the positional cell identity using early markers of telencephalic and subpallial identity (Fuccillo et al., 2006; Martynoga et al., 2005) (Figures 1B–1E and S1A–S1C). CYC cells expressed high levels of the dorsal marker *Pax6*, while SAG cells exhibited high expression of the ventral identity marker *Nkx2.1*. Moreover, both types of cells expressed high levels of the telencephalic marker *FoxG1* compared to control neuralized cells without Wnt inhibition (midbrain). We proceeded to analyze specific early markers of the subpallium and medial and lateral ganglionic eminences (MGE-LGE), such as *Lhx6*, *Lhx8*, *and Dlx1* (Chen et al., 2017) and *Ascl1*, *Dlx2*, *and Sfrp1* (Nery et al., 2002). We found an increased expression of these genes in SAG cells compared to the other neural population (Figures 1F and S1D–S1I).

Analysis of marker expression at the cellular level revealed that a high percentage of both CYC and SAG cells were positive for Foxg1 (Figures 1G and 1L). We observed that Nkx2.1 expression was largely restricted to SAG cells and was nearly absent in CYC cells (Figures 1H and 1L), while the dorsal markers Pax6 and Tbr1 were expressed in a large proportion of CYC cells but were barely detectable in SAG cells (Figures 1I, 1J, and 1L). By DIV20 (approximately equivalent to postnatal day P0 in mice), the cortical marker Satb2 was found in a significant fraction of CYC cells but was almost absent in SAG cells (Figures 1K and 1L). These data confirmed that SAG cultures have a ventral identity similar to the MGE and subpallium.

**Figure 2:**
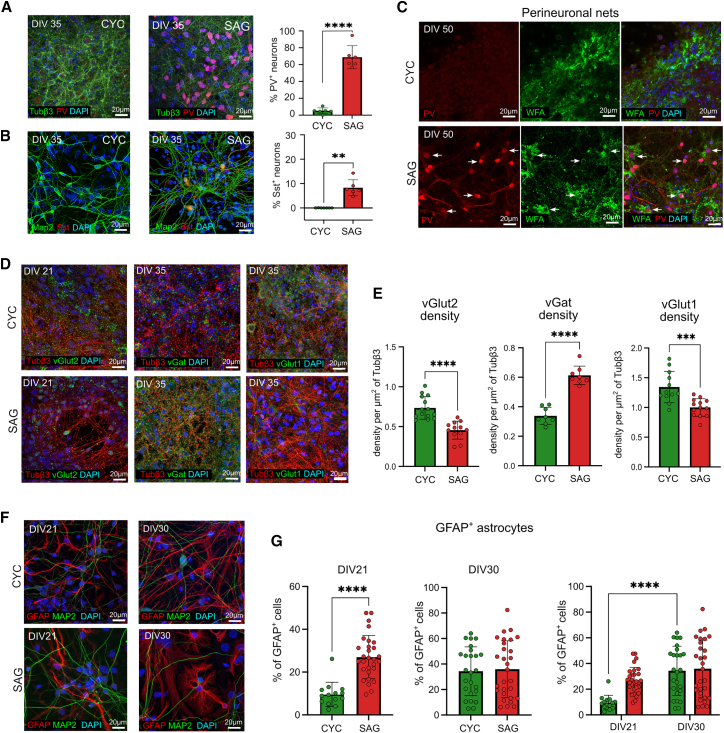
Expression of excitatory and inhibitory neuronal markers in CYC and SAG cultures (A and B) Representative images and quantification of CYC and SAG cells stained with Tubβ3, Map2, parvalbumin (PV), somatostatin (Sst), and DAPI at DIV35. Scale bar, 20 μm. Mean ± SD and unpaired t test are shown for *n* = 3 independent experiments: ^∗∗^*p* value < 0.01, ^∗∗∗∗^*p* value < 0.0001. (C) Representative images of CYC and SAG cells stained with the perineuronal net (PNN) markers PV and WFA at DIV50. White arrows indicate PNN-like structures surrounding PV^+^ neurons. Scale bar, 20 μm. (D) Representative confocal images of glutamatergic and GABAergic markers: vGlut2 (DIV21), vGat, and vGlut1 (DIV35). Scale bar, 20 μm. (E) Quantification of glutamatergic and GABAergic markers shown in (D). Synaptic vesicles were measured in terms of density of Tubβ3 positive area covered by vesicles. Comparisons showed statistical differences between the two treatments (*n* = 3 independent experiments; unpaired t test). (F and G) GFAP expression at DIV21 and DIV30 (Scale bar, 20 μm). In (G), mean ± SD and unpaired t test are shown at distinct DIV; two-way ANOVA followed by Šídák’s multiple comparison test was performed for comparison over time. *p* values: ^∗^*p* value < 0.05, ^∗∗^*p* value < 0.01, ^∗∗∗^*p* value < 0.001, ^∗∗∗∗^*p* value < 0.0001.

Since the MGE is the source of a wide variety of cortical interneurons (Gelman et al., 2011), we evaluated specific subtype markers such as parvalbumin (PV) and somatostatin (SST) (Figures 2A and 2B) (Lim et al., 2018). At DIV35, we counted 73% PV^+^ neurons in SAG cultures, while we found a much lower percentage (7%) in CYC cultures (Figure 2A). SST^+^ neurons accounted for less than 10% in SAG cultures and were virtually absent in CYC cultures (Figure 2B). Vasoactive intestinal peptide (VIP) neurons were not detected in either CYC or SAG cultures, suggesting that both culture conditions may lack progenitors from the caudal ganglionic eminence, where VIP^+^ neurons are predominantly generated (Marín, 2012). Given the association of PV^+^ interneurons with perineuronal nets (PNNs) (Lupori et al., 2023), we investigated also the reactivity against Wisteria floribunda agglutinin (WFA), a common marker for PNNs. A typical stained morphology of PNNs (Dickens et al., 2022), with this protein occasionally surrounding few PV^+^ cells (Figure 2C), was observed as early as DIV50, suggesting the formation of some early PNNs, mostly in SAG cultures.

The immunolabeling of distinct markers of excitatory (vGlut1 and vGlut2) and inhibitory (Pvalb and vGat) cortical neurons showed a different contribution of the two types of cells in SAG and CYC cultures, confirming the prevalence of excitatory and inhibitory neurons in CYC and SAG cultures, respectively (Figure 2D). We investigated the early glutamatergic marker vGlut2 at DIV25: we evaluated the density of vesicles by observing the colocalization of vGlut2 puncta with Tubβ3^*+*^ fibers and found a significantly higher density of vGlut2^+^ vesicles in CYC neurons as compared to the SAG neurons (Figure 2E). Moreover, by counting the number of DAPI-positive nuclei surrounded by vesicle transporters, we evaluated 70% of vGlut2^+^ cells in CYC cultures (Cao et al., 2017), while only 15% of them were present in SAG cultures (Kempf et al., 2021) (Figures S2A and S2B). We also analyzed the later glutamatergic marker vGlut1 and found a significantly higher density of vGlut1-positive puncta in CYC neurons as compared to SAG neurons (Figure 2E), with 80% of CYC neurons surrounded by VGlut1^+^ vesicles (Figures S2C and S2D). Focusing on GABAergic markers, we analyzed the vGat density at DIV35 in our cultures, highlighting a significantly higher number of vGat^+^ vesicles in SAG neurons as compared to CYC neurons (Figure 2E).

To assess the percentage of astrocytes in CYC and SAG cultures, we evaluated the presence of GFAP^+^ cells at two time points of maturation, DIV21 and DIV30. At DIV21, SAG cultures had a higher percentage of GFAP^+^ cells than CYC cultures, suggesting that they complete neurogenesis and start astrogliogenesis earlier. However, by DIV30, both cultures reached the same percentage (40%) of GFAP^+^ cells (Figures 2F and 2G), indicating that cell differentiation was completed.

Altogether, our data indicate that timely treatment with either CYC or SAG from DIV5 to DIV11 generated pallial neural progenitor cells (NPCs) with the competence to differentiate into neurons with gene expression profiles typical of DT and VT neurons, respectively. CYC cells produced mostly glutamatergic pyramidal neurons while SAG cultures were enriched in GABAergic cells, mainly PV^+^ interneurons, similar to those migrating to the embryonic developing cortex. Both cultures also produced an optimal ratio of astrocytes (40%), which is expected to support functional network activity.

### Morphological analysis of CYC and SAG neurons in mixed cultures

**Figure 3:**
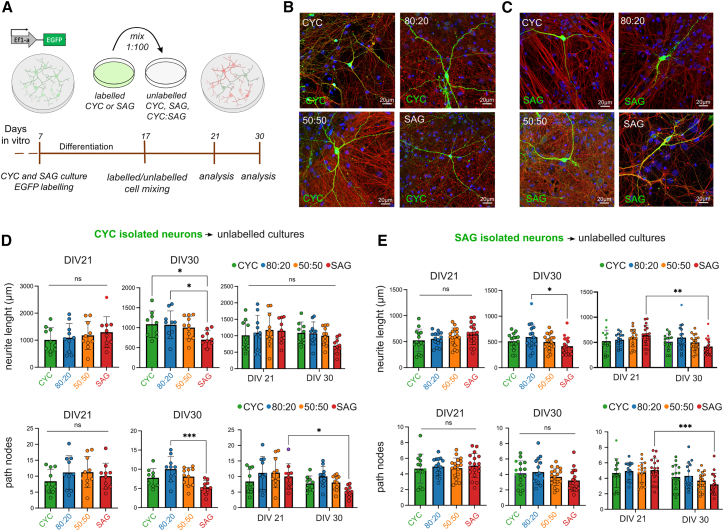
Morphological analysis of CYC and SAG neurons in pure and mixed cultures (A) Method outline. (B and C) Images of EGFP^+^-CYC or EGFP^+^-SAG neurons, respectively, in pure and mixed unlabeled cultures. Scale bar, 20 μm. (D and E) Quantitative analysis of neurite length and path nodes at DIV21 and DIV30. Mean ± SD and ordinary one-way ANOVA with Tukey’s multiple comparisons test are shown to compare samples at each time point; two-way ANOVA followed by Šídák’s multiple comparisons test was performed for comparison over time. *p* values: ^∗^*p* value < 0.05, ^∗∗^*p* value < 0.01, ^∗∗∗^*p* value < 0.001, ^∗∗∗∗^*p* value < 0.0001; ns, not significant.

The correct integration of VT neurons into the cortex is a regulated process requiring saltatory migration (Marín et al., 2010). We evaluated whether early SAG neurons can differentiate when mixed to isochronic CYC neurons and vice versa, in an *in vitro* environment and in the absence of regulated migration. To assess the maturation and development processes of CYC and SAG neurons, we firstly created four culture conditions: pure CYC, pure SAG, and mixed 80:20 and 50:50 (CYC:SAG) ratios. Hence, to enable the analysis of single-neuron morphology, we used EGFP-labeled CYC or SAG cells at DIV7 and mixed them at a 1:100 ratio into the four unlabeled cultures (Figures 3A–3C, S3A, and S3B). We observed CYC and SAG neurons at DIV21 (early development) and DIV30 (maturation onset), measuring neurite length, branching (nodes), and dendritic spine percentage. In the analysis, we avoided EGFP-labeled cells showing glial morphology (Figure S3E), which appeared in proportions similar to those detected through immunodetection.

At DIV30, we found that CYC neurons in the SAG environment showed decreased neurite length and fewer nodes compared to neurons in other environments, especially in 80:20 cultures, resulting in lower maturation (Figure 3D). Similarly, SAG neurons showed a significant decrease in the length of neurites in SAG cultures at DIV30 and over time, compared with those in 80:20 cultures. In addition, when comparing the two time points, we also observed a reduced number of nodes concluding that SAG neurons showed altered maturation in branching patterns (Figure 3E).

The analysis of the dendritic spines (Figures S3A and S3B) highlighted a reduced percentage of spines on CYC neurons in both 50:50 and SAG conditions at DIV21 and DIV30 (Figure S3C). In contrast, the maturation of SAG neurons’ spines seemed impaired at DIV21 in inhibitory-rich environments but showed no difference at DIV30 (Figure S3D). However, their spine percentage increased over time only in the 50:50 and SAG conditions, which is in line with the knowledge that medium spiny neurons in the striatum form abundant dendritic spines during development (Steiner and Tseng, 2017). Overall, our results indicate that the composition of the surrounding neuronal population might influence the morphological maturation of a neuron. We observed alterations in neurite length, branching complexity, and dendritic spine density in both CYC and SAG neurons when cultured in mostly inhibitory environments, suggesting that the diverse cellular environments may impact the functional integration and connectivity of these neurons within cortical circuits.

### Functional activity development of pure and mixed CYC and SAG neuronal networks

Primary cortical neurons in culture spontaneously develop neuronal networks with various activity patterns (Charlesworth et al., 2015; Dias et al., 2021). The E/I balance forms the basis for functional neural networks, supporting cognition and memory. This balance is maintained at a single-neuron level by an appropriate ratio of excitatory to inhibitory synaptic inputs and at the network level by regulating the interaction between various excitatory and inhibitory circuits (He and Cline, 2019). To simulate various E/I conditions, we analyzed pure CYC cultures (lacking ventrally migrated interneurons), pure SAG cultures (enriched with GABAergic neurons), and mixed CYC:SAG cultures with different E/I ratios. Our analysis focused on the development of network activity patterns, specifically targeting the most abundant inhibitory neuron types, PV^+^ and SST^+^ neurons, which were maintained at consistent proportions throughout the maturation period. The immunostaining analysis showed that the relative proportions of PV^+^ (Figures S4A and S4B) and SST^+^ (Figures S4C and S4D) neurons remained the same at DIV30 and DIV45 (Figures S4E and S4F).

**Figure 4:**
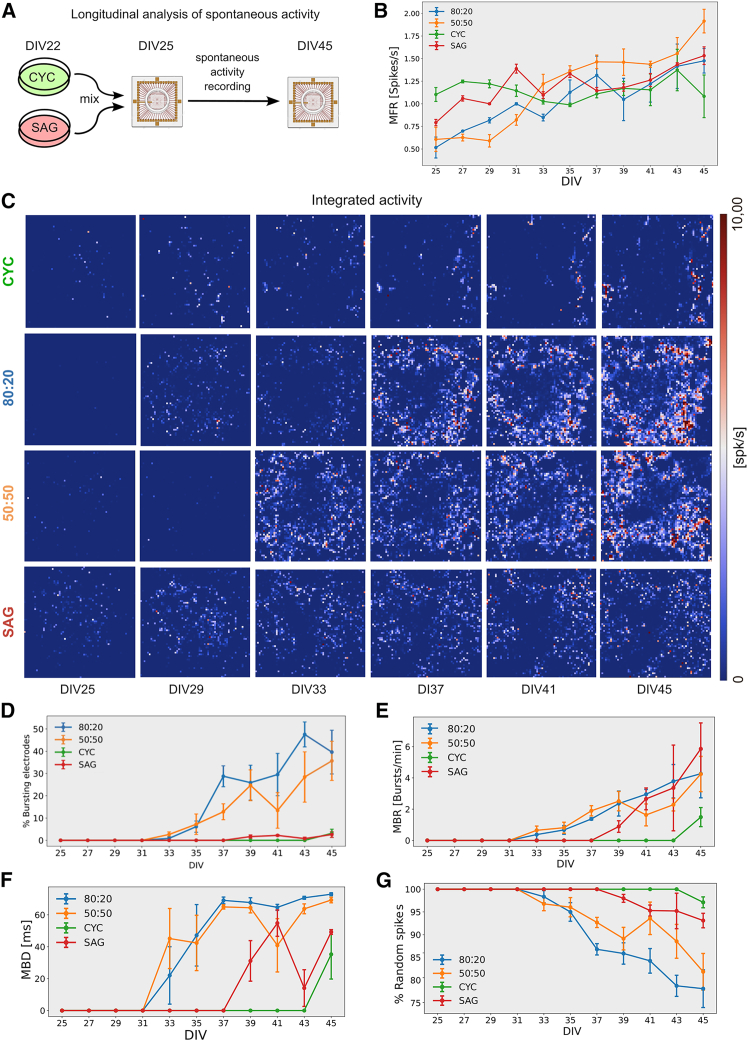
Longitudinal analysis of electrophysiological activity of CYC, SAG, and mixed cultures (A) Experimental outline. (B) Mean firing rate (MFR) of individual channels over time (*n* = 3 independent experiments; mean ± SEM is shown). (C) Firing rate (spk/s) heatmap of representative HD-MEAs; each pixel represents the integrated activity (5 min) of one channel at different DIV. (D–G) Percentage of bursting electrodes, mean burst rate (MBR), mean burst duration (MBD), and percentage of random spikes of individual channels at distinct DIV (*n* = 3 independent experiments; mean ± SEM is shown).

Using a high-density microelectrode array (HD-MEA) comprising 4,096 channels, we conducted a longitudinal study of pure CYC and SAG cultures and mixed cultures with an 80:20 or 50:50 ratio (Figure 4A). As CYC culture contained 7% of PV^+^ cells and a negligible ratio of SST^+^ cells (Figure 2A) and SAG culture contained 73% of PV^+^ cells and 7% of SST^+^ cells (Figure 2B), the theoretical number of PV^+^ and SST^+^ inhibitory neurons in 80:20 and 50:50 cultures is 21.6% and 43.5%, respectively. It should be noted, however, that the relative proportions of PV^+^ and SST^+^ neurons in both pure and mixed cultures differ from those observed in the cortex, where the ratio of inhibitory interneurons PV:Sst:VIP is approximately 40:30:30 (Druga et al., 2023), thus representing an artificial experimental condition.

We first analyzed the mean firing rate (MFR) per channel, the mean burst rate (MBR) and duration (MBD), and the percentage of bursting electrodes and of random spikes. MFR increased over the time in all cultures with no significant differences between them (Figure 4B; Videos S1, S2, S3, S4, and S5). However, a clear difference was observed in bursting activity. Mixed cultures had a much higher number of bursting electrodes (Figures 4C and 4D, Supplementary movies VS1,3,4), with bursts appearing first in 50:50 and 80:20 cultures (DIV33), followed by SAG cultures (DIV39) and finally CYC cultures (DIV45) (Figures 4E and 4F). MBR and MBD were also higher in mixed cultures compared to pure cultures (Figures 4E and 4F), indicating that combining the two cell types greatly enhances the ability to generate burst activity. Pure cultures showed the highest percentage of random spikes (Figure 4G), despite having a comparable MFR to mixed cultures (Figure 4B).

Video S1. Network activity heatmap of CYC, SAG, 80:20 and 50:50 cultures

Video S2. CYC culture firing activity

Video S3. 80:20 culture firing acitvity

Video S4. 50:50 culture firing activity

Video S5. SAG culture firing activity

The pure CYC network developed different adhesion properties than SAG cultures (Figures S4G–S4I). They tended to form clusters (Figure S4G) with a thicker cell layer than SAG cultures (Figure S4I), although the average cell density was comparable between the two types of cultures (Figure S4H), suggesting different adhesion and connectivity properties. This led to a lower number of active channels in CYC cultures compared to mixed and SAG cultures (Figure S4J). However, the analysis of double-density cultures confirmed that the difference in activity between CYC and 80:20 networks was not due to cell proportion on the electrodes (Figure S4K).

Because SAG cultures are predominantly inhibitory (80% inhibitory neurons) and CYC cultures have only 7% inhibitory neurons, it appears that inhibitory neurons play a key role in establishing bursting activities. To test this, we simulated self-organizing artificial neural networks with varying neuronal E/I ratios using the Izhikevich spiking neuron model (2006). The model is simpler and faster than more complex biophysical models, making it ideal for studying large-scale networks (Izhikevich and Edelman, 2008). Our simulations of a 1,000-neuron network with varying E/I ratios showed that the Instantaneous Network Firing (INF, see methods), a metric that in Izhikevich’s formulation most closely resembles the burst rate observed in biological networks, followed a trend similar to our biological networks: maximum INF occurred at E/I ratios between 800:200 and 500:500 (Figure S4L). Although the model aims to replicate the excitability and connectivity characteristics of cortical excitatory and inhibitory neurons, it remains a simplification. Nonetheless, the consistent results across two fundamentally different network types suggest that the E/I ratio’s effect on burst activity is an emergent property arising from complex network interactions rather than being solely determined by individual neuronal characteristics.

### Pure and mixed CYC and SAG cultures show distinct patterns of network burst activity

**Figure 5:**
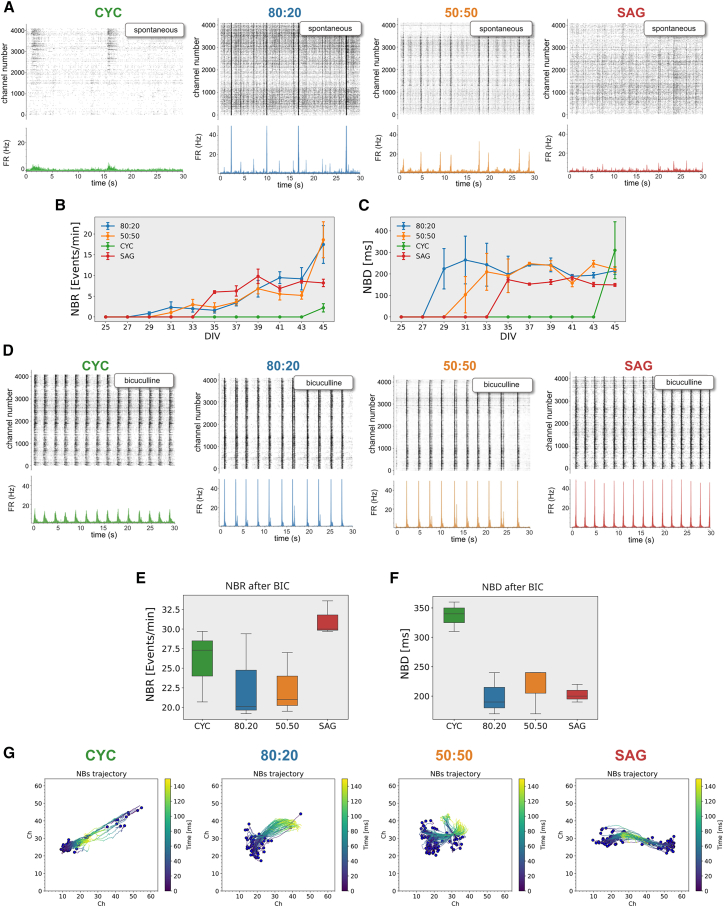
GABA inhibition critically affects network activity in pure and mixed CYC and SAG cultures (A) Top: activity raster plot (spike times per channel) of representative HD-MEA at the analysis endpoint (DIV45); bottom: average network firing rate. (B and C) Network burst rate (NBR) and network burst duration (NBD) (*n* = 3 independent experiments; mean ± SEM is shown). (D) Analysis as in (A), after BIC administration. (E and F) NBR and NBD after BIC administration (*n* = 3 independent experiments). (G) Analysis of the center of activity trajectories (CATs). Each blue dot represents the physical center of mass of the activity where the NB starts, while the colored line represents its own trajectory. The colored scale bar represents the time elapsed during the propagation of an NB.

Spontaneous network activity underpins the development of functional networks in early stages (Teppola et al., 2019), and a key feature of this activity is the recurrence of intense network bursts (NBs) that rapidly propagate throughout the culture (Weihberger et al., 2013). We analyzed NBs in our cultures and found that they first appeared in mixed CYC:SAG cultures starting from DIV31 and then in pure SAG cultures, although at a very low extent, from DIV35, whereas they were almost absent in pure CYC cultures (Figures 5A and 5B).

At DIV45, NB duration (NBD) was comparable in the four cultures (Figure 5C), indicating that this parameter is independent of the NB frequency. The contrasting NB trends between CYC and 80:20 cultures, despite both having low PV^+^ and high glutamatergic neuron percentages, suggest a difference in their excitatory/inhibitory connectivity. We thus investigated the role of GABA, AMPA, and NMDA receptors in NB occurrence of mature networks (DIV45). Antagonists for AMPA (CNQX) and NMDA (AP5) receptors blocked NBs in all cultures (Figures S5A–S5E), consistent with their known role in burst activity (Jimbo et al., 2000). As the four cultures have different degrees of GABAergic signaling, mainly due to the presence of different percentages of PV^+^ and SST^+^ interneurons, they respond differently to the administration of bicuculline (BIC), a GABA_A_ receptor antagonist. Mixed 80:20 and 50:50 cultures showed a slight increase in NB rate after BIC administration, while pure CYC and SAG cultures showed a massive increase in both rate and duration (Figures 5D–5F). Notably, CYC NBR was even higher than the 80:20 one, suggesting that this effect involves the activity recruitment of new neurons and is independent of cell density (see Figure S5F). This observation in the CYC culture, despite its low number of inhibitory neurons, indicates that the ratio of PV^+^ and SST^+^ cells does not directly correlate with NB activity. We believe that this may be due to the development of different structural and functional connectivity patterns, although this hypothesis requires further experimental investigation.

Using center of activity trajectory analysis (Chao et al., 2007) to quantify network burst events, we found that when inhibitory activity was suppressed by BIC, NBs propagated with similar properties across all four cultures (Figure 5G). In conclusion, BIC appeared to unmask an intrinsic capacity of CYC and SAG cultures to generate and propagate NBs.

### GABAergic and glutamatergic components differently impact on pure and mixed CYC and SAG functional networks

**Figure 6:**
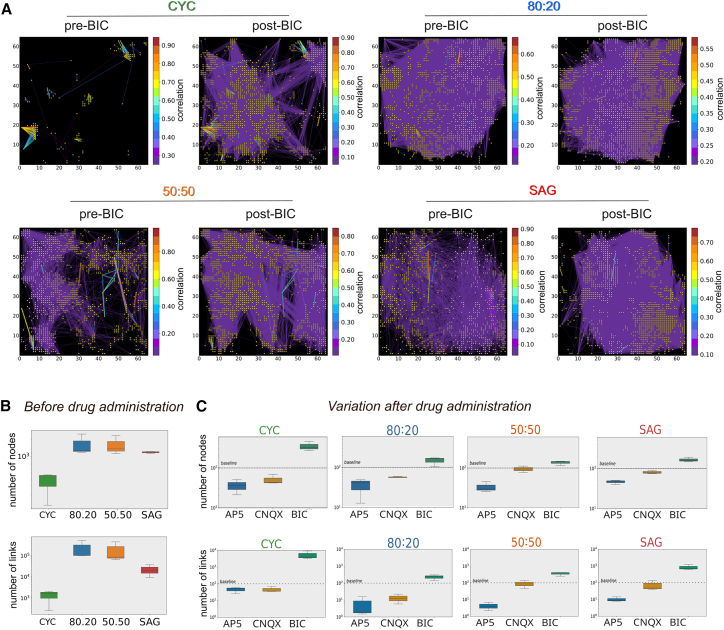
GABA inhibition unmasks complex connectivity of the CYC network (A) Connectivity plots of representative neuronal cultures during spontaneous activity before (pre-BIC) and after (post-BIC) BIC administration. Each yellow point represents a node of the functional graph; colored lines represent the correlation strength between two points (only the 10% of the functional links are shown). The color bar indicates the correlation index. (B and C) Number of nodes and links before and after drug administration, respectively (*n* = 3 independent experiments).

As GABAergic inhibition is responsible for masking the intrinsic NB activity of pure cultures compared to mixed cultures, we investigated whether a specific functional connectivity analysis could explain the different effect of BIC on the NB activity of the four conditions. We compared functional network connectivity, which is the activity correlation between channels, before and after administering BIC (Figure 6A), CNQX, and AP5 (Figure S6). The correlation analysis allowed us to calculate the number of nodes and links of the spontaneous network activity (Figure 6B) and their changes after drug administration (Figure 6C). In the absence of drugs, the mixed cultures showed a similar number of nodes and links while SAG and CYC networks had fewer, with CYC showing the lowest number (Figure 6B). Each drug affected the four cultures differently (Figures 6C and S6). BIC had its most pronounced effect on CYC cultures, where it dramatically increased the number of nodes and links. This is significant because SAG cultures have a much higher proportion of PV^+^ and SST^+^ neurons than CYC cultures. Our results suggest that a very complex part of the CYC functional network was under strong GABAergic inhibition. This is consistent with studies showing that PV^+^ interneurons can significantly influence the excitatory activity of pyramidal neurons to compensate for overactive excitatory forces (Haider et al., 2006; Xue et al., 2014). Finally, while CNQX had almost no effect on SAG and 50:50 cultures as compared to CYC and 80:20, AP5 decreased the number of nodes and links in all the cultures, although at different extents (Figure 6C). This indicates that the activity of NMDA receptors is important in the developmental regulation of synaptic transmission, also mediated by both AMPA and GABA_A_ receptors (Lu et al., 2011; Marsden et al., 2007), and plays a pivotal role in establishing NB activity and maintaining the delicate E/I balance.

### Network stimulation discloses different signal propagation properties of pure and mixed cultures

**Figure 7:**
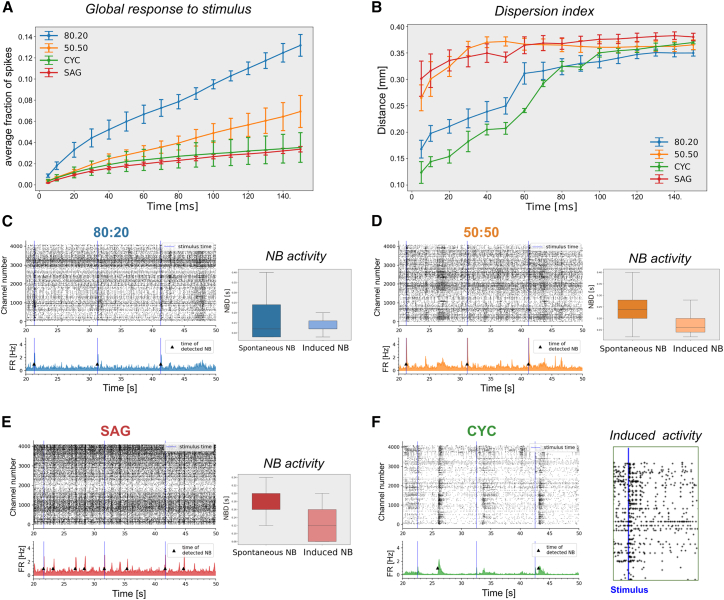
Global response, dispersion index, and NBs evoked by stimulus are affected by E/I ratio (A) Distribution of the global response to single-channel stimulation, showing the average fraction of spikes starting from 5 to 150 ms (*n* = 3 independent experiments; mean ± SEM is shown). (B) Dispersion index indicating the distance of responses from the stimulated channel at different times from the stimulus (*n* = 3 independent experiments; mean ± SEM is shown). (C–F) Representative raster plots (C–F) and boxplots (C–E) showing NBs and NBD, respectively, during multiple electrode stimulation. The stimulus time is indicated by the blue line. On the right in (F): enlarged detail of CYC raster plot, highlighting only the electrodes that show a response to the stimulus (*n* = 3 independent experiments).

We analyzed the patterns of activity evoked by electrical stimulation in our cultures and compared them to the patterns of spontaneous activity. At DIV50, we stimulated a single channel with 25 biphasic pulses. We measured the response by comparing spike counts before and after each pulse and averaging the responses over 25 repetitions. The single-channel stimulation evoked the activity of channels located at different distances within a 5 ms delay, suggesting direct functional connectivity (Figures 7A and S7A–S7D). The 80:20 condition was the most responsive, with the stimulus first recruiting a high number of positively correlated channels (red channels in Figure S7B), followed by the appearance of negatively correlated channels (yellow channels in Figure S7B). In 50:50 and SAG cultures, the induction of positively correlated active channels was moderate, while the appearance of negatively correlated channels was more pronounced and earlier compared to 80:20 cultures (Figures S7C and S7D). CYC cultures behaved differently, showing recruitment of almost all activated channels within 5 ms and appearance of negatively correlated channels with a 60 ms delay, more like the 80:20 culture (Figure S7A).

We also analyzed the spatial dispersion of the signal based on the physical distance from the stimulation point (dispersion index in Figure 7B). Our findings reveal a correlation between the dispersion index of the signal evoked by the stimulus and the PV^+^ cell ratio. All four cultures had a common peak in the dispersion index around 0.3–0.4 mm at 150 ms post stimulation. The 80:20 cultures had the most robust overall response but a low dispersion index at early times, suggesting a tendency to form local circuits. Conversely, 50:50 and SAG cultures showed more distant initial responses but a significantly lower overall response. We hypothesized that stronger inhibition in these cultures may locally suppress the initial response, promoting it farther from the stimulation site. We concluded that intermediate ratios of inhibitory neurons provide the best global activity due to a balance between signal propagation and inhibition.

Single-channel stimulation was never able to propagate to the entire network and to generate NBs. We speculated that stimulating a sufficient number of spontaneously active electrodes could evoke NBs. We selected the 7 most active channels in each culture for simultaneous stimulation. In 80:20, 50:50, and SAG conditions, the electrical stimulation evoked a synchronized network activity comparable to spontaneous NBs (Figures 7C–7E). CYC cultures did not generate NBs in response to the stimulus (Figure 7F): indeed, the response did not propagate beyond the activated channels (enlarged detail of the raster plot in Figure 7F). This last observation suggests that a proper inhibitory ratio is required to integrate and organize a network circuit capable of propagating NBs (Tremblay et al., 2016). Moreover, we evaluated the duration of the induced NB (NBD). In the 80:20 configuration (Figure 7C), the duration was similar to the one of spontaneous NBs, leaving the network almost unchanged. In 50:50 and SAG conditions, the duration of the induced NBs was shorter than the spontaneous NBs (Figures 7D and 7E) and chemically induced NBs (Figure 5F). These findings demonstrate that the presence of PV^+^ interneurons significantly impacts the dispersion of neuronal activity, influencing network connectivity and determining the capacity of cortical networks to generate synchronous NBs.

## Discussion

We studied how isolated and mixed cultures of DT and VT neurons develop and mature *in vitro*. Our analysis showed that these cultures, which have different molecular identities, also develop distinct network activity patterns.

CYC and SAG cultures expressed key marker genes consistent with DT and VT identity and matured opposite ratios of PV^+^ and SST^+^ inhibitory interneurons, very high (73% and 7%) in SAG cultures and very low (7% and less than 1%) in CYC cultures. Moreover, CYC cultures were enriched in glutamatergic vGlut1^+^ neurons (73%) whereas vGlut1^+^ neurons were 16% in SAG cultures. We observed that isolated CYC and SAG neurons could mature in each other’s cultures, allowing us to investigate the activity properties of networks with different E/I balance. We aimed to understand the basic requirements for generating neuronal networks that can produce spontaneous, correlated activity, similar to what’s seen in early brain regions (Chiu and Weliky, 2001; Crochet et al., 2005). Although pure CYC cultures and mixed cultures had a similar MFR, the pure CYC cultures showed very little correlated (burst) activity. This changed when a low percentage of SAG neurons were added: the 80:20 cultures exhibited more synchronous firing patterns. This also reduced the percentage of random spikes and increased the number of bursting electrodes, significantly boosting the network burst rate (NBR) and NBD. This is a puzzling finding, because SAG cultures, which contain 80% PV^+^ and SST^+^ GABAergic neurons, show almost no network bursting activity on their own.

Sukenik et al. sorted hippocampal neurons from E17 mouse embryos by fluorescence-activated cell sorting into GAD^+^ and GAD^−^ populations, which were then seeded at different ratios onto glial layers in microfluidic chambers (Sukenik et al., 2021). Using patch-clamp analysis, the authors observed that an increase in the percentage of inhibitory neurons in primary neuronal cultures leads to a reduction of the total number of active incoming connections received by a neuron. This mechanism allows the network to maintain a similar E/I balance and, consequently, to stabilize its spontaneous excitatory activity, adapting to diverse cellular compositions. While this observation is in line with the similar values of channel MFR that we observed in our different cultures (Figure 4B), it does not explain the lack of proportionality between E/I and synchronous network activity. Thus, we speculated that a proper ratio of GABAergic neurons is required to mature local circuitry capable of burst activity formation and spreading. Our findings indicate that bursting activity peaks within an intermediate range of E/I ratios, both *in vitro* and *in silico* networks. The remarkable consistency across these disparate network models—namely *in vivo* biological systems and *in silico* computational simulations—suggests that the observed relationship between E/I ratio and bursting activity is not merely a sum of individual neuronal characteristics. Instead, it appears to be an emergent property, arising from the intricate and complex interactions within the neural network itself. This emphasizes the critical role of network dynamics in shaping neuronal activity patterns, rather than solely attributing them to intrinsic cellular properties. However, the mechanistic explanation of this phenomenon remains to be addressed both through *in silico* network models and in biological networks.

The presence of different circuits in pure and mixed cultures is suggested by the different types of network activity generated under spontaneous conditions, upon GABAergic release by BIC administration or electrical stimulation. CYC cultures, which showed the lowest spontaneous NBR, generated the highest NBR together with SAG cultures after BIC treatment but were unable to induce NBs upon electrical stimulation. These observations, together with the shortest dispersion index shown by CYC cultures, point out that a too high E/I ratio develops a different network circuitry, confirming that an optimal E/I ratio is required to form local inhibitory networks capable of developing and spreading correlated activity.

Few studies have so far modeled cortical networks with precise E/I ratios. Different E/I ratios of iNeurons were used to evaluate the seizure liability of compounds by microelectrode array (MEA), identifying the 84/16 E/I balance as the best suited to detect concentration-dependent changes and to classify the mechanism of action of seizurogenic compounds (Yokoi et al., 2022). Interestingly, this intermediate E/I balance showed the highest NB activity compared to higher and lower balances, in accordance with our observations that NBs are better supported by an intermediate E/I balance. Yokoi and co-workers observed that iNeuron cultures with the lowest E/I balance have the highest activity and the lowest response to GABA inhibitors, contrary to our observations in mESC cultures. A possible explanation for this difference could lie in the diverse characteristics and composition of the cell subtypes (i.e., different ratios of PV^+^ SST^+^ interneurons) and the connectivity of the circuits formed in the two types of culture. MEA recordings were also utilized by other groups to study hiPSC-derived networks with varying E/I balance (see introduction) (Parodi et al., 2023, 2024). These studies, which used different types of iNeurons, reported contrasting behaviors of networks with the highest E/I balance (100:0), showing lower or higher network activity and NBR than the other E/I balances in the case of low- or high-density MEAs, respectively. However, the different molecular nature of iNeurons compared to our cultures, especially the expression of non-telencephalic markers (Lin et al., 2021) and the lack of PV^+^ interneurons (Zhang et al., 2013) (see introduction), makes it difficult to compare the different datasets.

In our study, we expanded the analysis of various networks by examining their capacity to propagate stimulus-induced activity, underscoring the pivotal role of E/I balance. In addition, our study analyzes the network connectivity at two levels, structural (dendritic branching and number of nodes, Figure 3) and functional (Figures 4, 5, 6, and 7). By taking into account these network parameters, which were neglected in previous research, we could better explain divergent activity patterns in networks with different E/I balances.

Our current *in vitro* cortical network model, derived from *in vivo* observations, has limitations compared to an intact *in vivo* network. These include differing ratios of PV^+^ and SST^+^ inhibitory interneurons, absence of VIP^+^ neurons, lack of thalamic afferents, and the general absence of vascularization, microglia, and fine cellular complexity. Despite these differences, our findings suggest that our model offers significant advantages over iNeuron-based models, as it more faithfully recapitulates the cortical identity of glutamatergic neurons and PV^+^ interneurons. When applied to hiPSs, it represents a powerful tool for studying the role of E/I balance in shaping network activity during human neurodevelopment, particularly in the context of neurodevelopmental disorders.

## Methods

mESCs were differentiated into DT or VT lineages following a four-step protocol. In the first step (DIV0–DIV5), cells were cultured in a chemically defined minimal medium supplemented with Wnt and BMP inhibitors (WiBi) and plated on poly-ornithine/laminin-coated (PL) surfaces. In the second step (DIV6–DIV10), DT and VT cells were generated adding cyclopamine (3 μM) and SAG (0.1 μM), respectively, to the WiBi medium. In the third step (DIV11-DIV20), cells were replated onto PL and maintained in neurobasal-A. From DIV20 onward, neurons were maintained in neurobasal-A medium supplemented with 0.2 mM ascorbic acid and 20 ng/mL recombinant human BDNF protein. To prepare the mixed cultures of 80:20 and 50:50, neurons were splitted at specific time points. For MEA preparation, CYC and SAG neurons were detached at DIV22, counted, mixed with the designed proportions and seeded on sterilized HD-MEA (Accura, 3Brain) (60,000 cells/chip), and allowed to adhere O/N.

Quantification analyses for density of glutamatergic and GABAergic vesicles were done by using the ImageJ Synapse Counter plugin (https://github.com/SynPuCo/SynapseCounter). To study neuronal development in pure and mixed cultures, CYC and SAG cells were transduced with EGFP lentivirus at DIV7 and selected via PuroR. At DIV17, EGFP-labeled and unlabeled neurons were combined to create pure (cyclopamine-only, SAG-only) and mixed (80:20, 50:50) cultures, each with 1% EGFP-positive cells, and seeded on PL-treated glass (150,000 cells/cm^2^). Cultures were fixed with 2% paraformaldehyde at DIV21 and DIV30 and analyzed by immunofluorescence. Neural length and the node number were evaluated by the Fiji SNT plugin.

Electrophysiological recordings were conducted using high-density CMOS-based 4096 microelectrode arrays (Accura, 3Brain) to evaluate spiking activity, bursting behavior, and network synchronization. Drug responses and functional connectivity were analyzed using custom Python scripts, with spike sorting and network analysis performed using principal-component analysis and cross-correlation methods.

The response to stimuli was measured by comparing spike counts before and after the stimulus within a defined time window (5–150 ms). The stimulus was repeated 25 times, responses were averaged, and statistical confidence intervals were calculated. For data visualization, responses were displayed on a grid, with pixel intensity indicating response strength. The dispersion index, quantifying how spread out significant responses were across the grid, was calculated by estimating the average distances between responsive electrodes.

The self-organizing artificial neural network modeling was performed as described by Izhikevich, 2006 (see supplemental methods).

Comprehensive descriptions of all experimental procedures are provided in the methods section of the supplemental information.

## Resource availability

### Lead contact

Further information and requests for resources and reagents should be directed to and will be fulfilled by the lead contact, Federico Cremisi (federico.cremisi@sns.it).

### Materials availability

This study did not generate new unique reagents. However, any questions about reagents or animals used can be directed to the lead contact.

### Data and code availability

This study did not generate new sequencing data. Any questions about gene expression experiments can be directed to the lead contact.

## Acknowledgments

We are thankful to Robert Vignali and Lucio Calcagnile for helpful discussions, Maria Antonietta Calvello, Vania Liverani, and Alessandro Puntoni for technical support, Michèle Studer and Michele Bertacchi for advise in marker immunodetection analysis, and Stefano Guglielmo, Claudia Alia, and Nicola Origlia for advice on the functional analysis of cultured networks. The research was supported by the Matteo Caleo Foundation, by intramural funding of 10.13039/501100009531IIT (L.P.) and 10.13039/100009093Scuola Normale Superiore (F.C.), by the PRIN grant #2022M95RC7 from the Italian Ministry of University and Research (F.C.), and by the Tuscany Health Ecosystem – THE grant from MUR (F.C. and A.D.G.). This work was funded by the European Union – Next Generation EU, Mission 4 Component 1 CUP E53C24001460006, project TNE – NEUROBRIDGE, which covered the publication costs.

## Author contributions

E.C. and F.C. conceptualized and designed the study and wrote the manuscript. E.C., F.T., and L.I. performed the experiments. E.C. set up the strategy of culture treatments, the connectivity analysis, and the longitudinal functional study. F.T. set up MEA stimulation. L.P. conceptualized the experiments of gene expression analysis. L.I. set up the time-series analysis of MEA data under the advice of A.D.G. G.L. performed the analysis of MEA stimulation under the supervision of G.A. M.F. modeled the artificial neural networks with STDP. All authors discussed the results and commented on the manuscript.

## Declaration of interests

The authors declare no competing interests.

## Supplemental information

Document S1. Figures S1–S7, Tables S1 and S2, and supplemental methods

Document S2. Article plus supplemental information
