# Supplementary material for: A proper excitatory/inhibitory ratio is required to develop synchronized network activity in mouse cortical cultures
Source: Stem Cell Reports. 2025 Sep 25;20(10):102646. doi: 10.1016/j.stemcr.2025.102646 (PMC12790722; doi:10.1016/j.stemcr.2025.102646)
Supplement: Document S1. Figures S1–S7, Tables S1 and S2, and supplemental methods [file mmc1.pdf]

Stem Cell Reports, Volume *20*

Supplemental Information

A proper excitatory/inhibitory ratio is required to develop synchronized network activity in mouse cortical cultures

Eleonora Crocco, Ludovico Iannello, Fabrizio Tonelli, Gabriele Lagani, Luca Pandolfini, Marcello Ferro, Giuseppe Amato, Angelo Di Garbo, and Federico Cremisi

Supplemental Figures

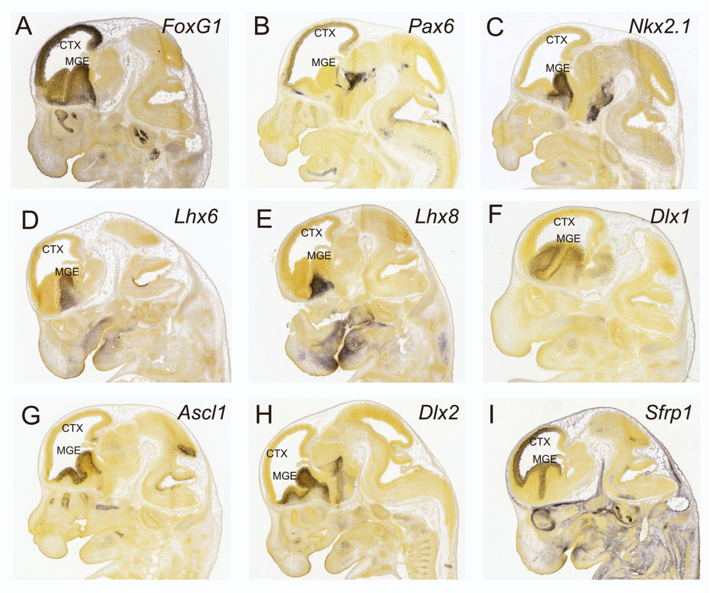

Figure S1. Positional markers of dorsal and ventral telencephalon. A-I) ISH of P13.5 mouse showing the expression of markers of telencephalic (A), dorsal (B), ventral (C) and subpallial (D-I) identity analyzed in Figure 1C-F. CTX: cortex; MGE: medial ganglionic eminence. Images from Allen Brain Atlas: Developing Mouse Brain (https://developingmouse.brain-map.org/).

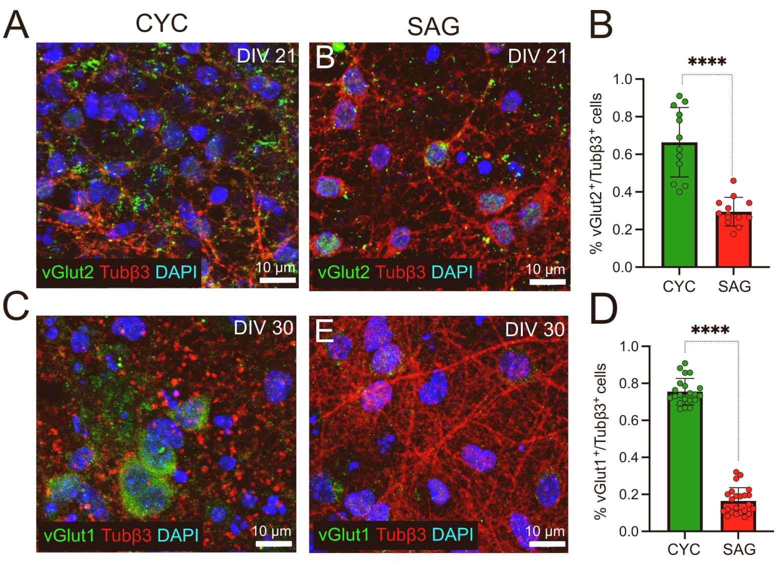

Figure S2. Glutamatergic marker expression in CYC and SAG cultures. A,C) Representative images of vGlut2⁺ and vGlut1⁺ neurons in CYC and SAG neurons at DIV21 and DIV30. B,D) Percentages of vGlut1 and vGlut2 positive neurons, respectively, in CYC and SAG cultures (n = 3 independent experiments). Positive cells were evaluated as Tubβ3 positive neurons surrounded by synaptic vesicles. Mean ± SD is shown, unpaired t-test, ****p-value < 0.0001.

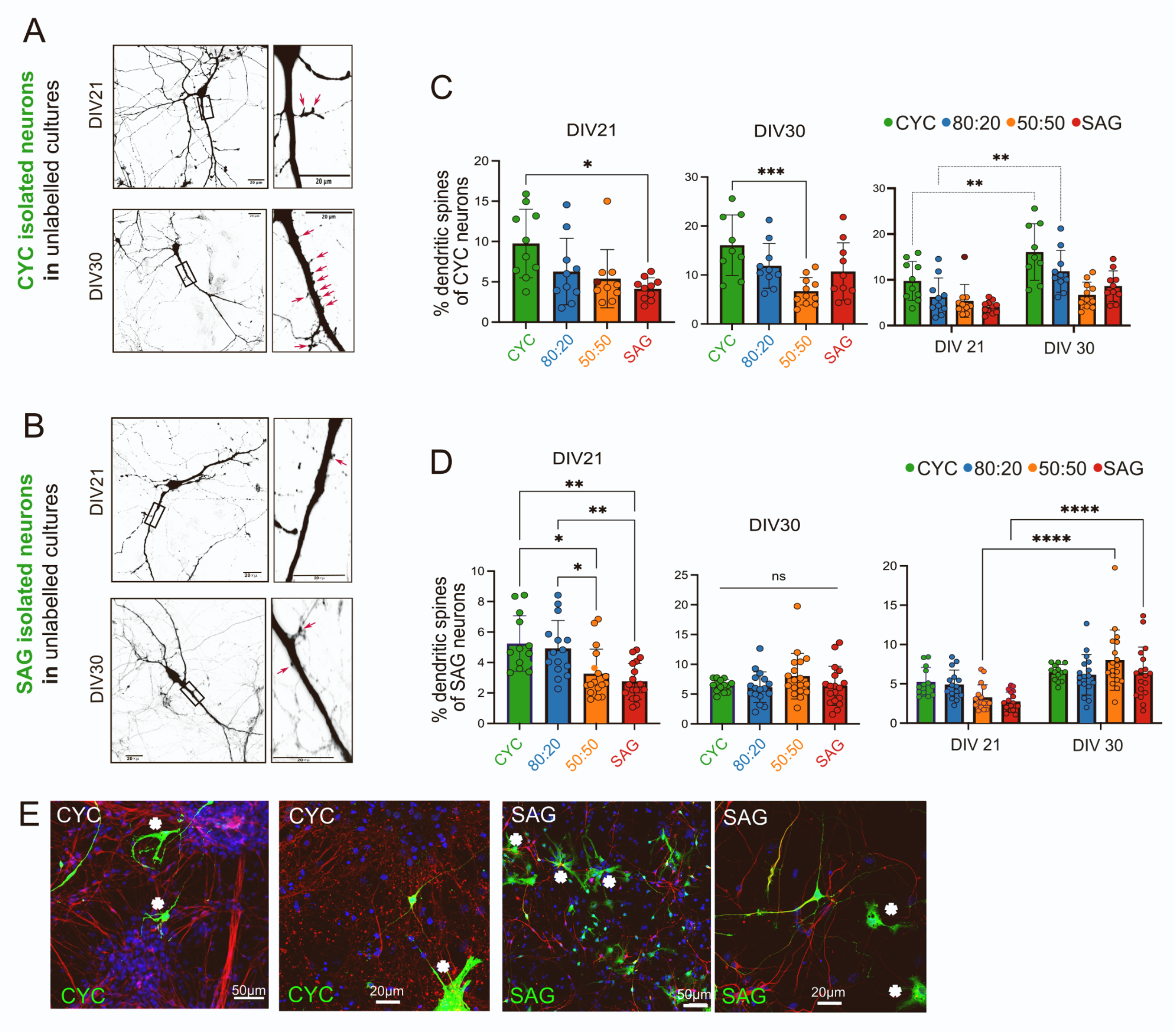

Figure S3. Spine analysis of CYC and SAG labeled neurons in pure and mixed CYC and SAG unlabeled cultures.

A,B) Representative images of dendritic branching of CYC and SAG EGFP-labeled neurons in pure and mixed cultures of unlabeled cells at DIV21 and DIV30. Insets on the right show enlarged details of the main image, with spines indicated by red arrows. Spines were identified according to Miura et al., (2020) using the Simple Neurite Tracer (SNT) toolbox (see Methods). C,D) Quantification of dendritic spines of CYC (C) and SAG (D) neurons at DIV21 and DIV30; mean ± SD is shown. Ordinary one-way ANOVA with Tukey's multiple comparisons test was performed to compare samples at each time point, and two-way ANOVA with Šídák's multiple comparisons test was performed for comparisons over time. N = 3 independent experiments; p-values: *p-value < 0.05, **p-value < 0.01, ***p-value < 0.001, ****p-value < 0.0001; ns = not significant. F) Examples of astrocytes (asterisks) labeled by the EGFP lentivector and not included in the analysis.

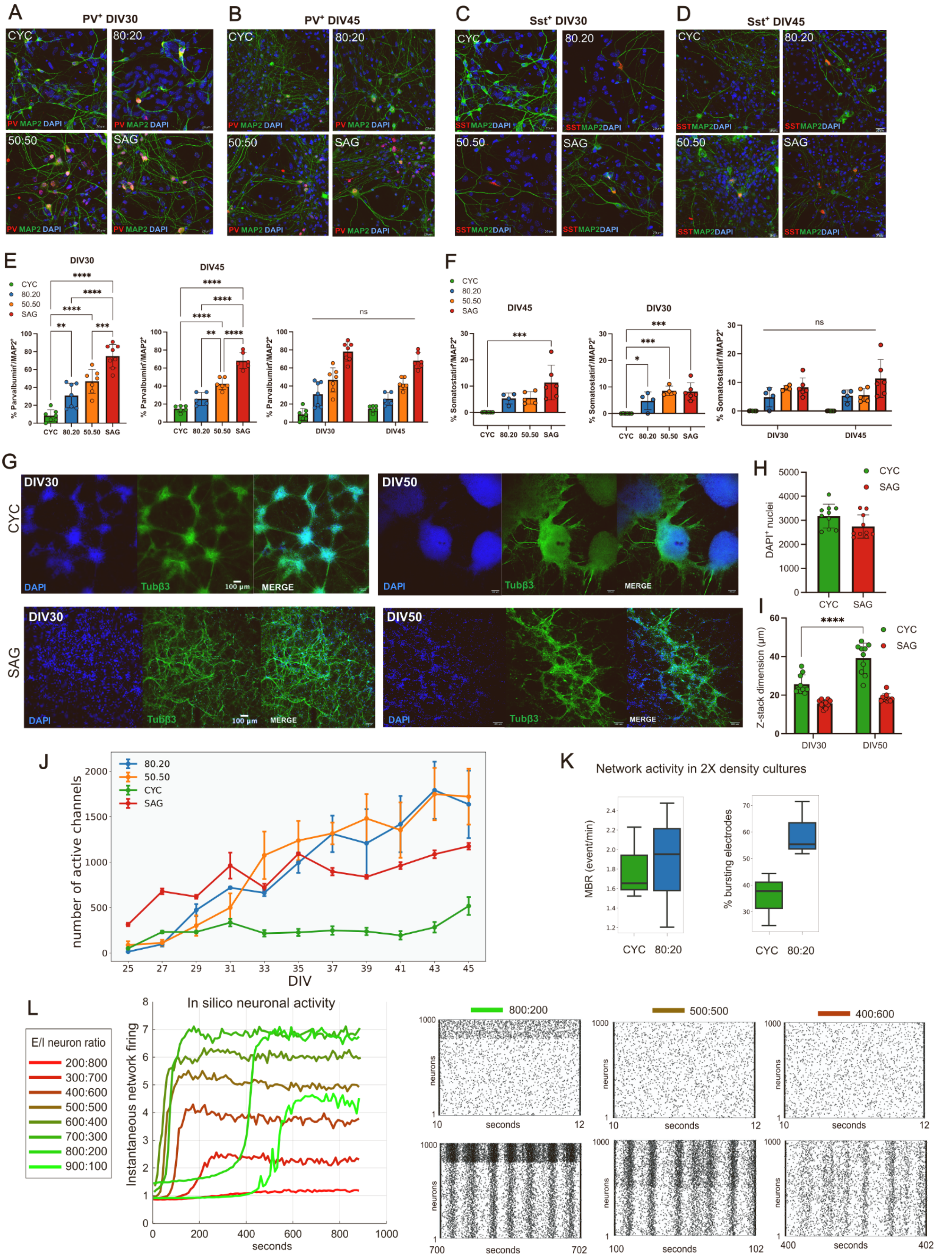

Figure S4. ***In vitro*** **and** ***in silico*** **analysis of networks with different E/I balance.** A–D) Representative immunodetection images of pure and mixed CYC and SAG cultures. Parvalbumin (PV) is shown in A and B, Somatostatin (SST) in C and D. Images were taken at DIV30 in A and C, and at DIV45 in B and D. E,F) Quantification of positive cells as in A-D); mean ± SD is shown; Ordinary one-way ANOVA with Tukey's multiple comparisons test was performed to compare samples

at each time point, and two-way ANOVA followed by Šídák's multiple comparisons test was performed for comparisons over time. P-values: **p-value < 0.01, ***p-value < 0.001, ****p-value < 0.0001, ns = not significant; (n = 3 independent experiments). G) Representative images of CYC and SAG cultures at DIV30 and at DIV50, showing morphological differences between the two cultures. H) Quantification of DAPI$^+$ nuclei in each image (n = 3 independent experiments); mean ± SD is shown. I) Quantification of cluster size in CYC and SAG cultures (n = 3 independent experiments); mean ± SD is shown; two-way ANOVA followed by Šídák's multiple comparisons test was performed; ****p-value < 0.0001. J) Number of active channels in each condition over time (n = 3 independent experiments; mean ± SEM is shown). K) MBR and percentage of active electrodes at DIV 45 in CYC and 80:20 cultures seeded at double cell density (to be compared to cultures shown in Figure 4D,E). L) *In silico* modeling of network activity. Spiking activity is shown from a numerically integrated Izhikevich neuron network of 1000 neurons, varying the E/I neuronal ratio from 900:100 to 200:800. Each neuron formed 100 random connections with other neurons. The network incorporated axonal conduction delays, with excitatory synapses having random delays up to 20 ms and inhibitory synapses fixed at 1 ms. Excitatory synaptic strengths underwent Spike-Timing Dependent Plasticity (STDP), dynamically adapting based on the precise relative timing of pre- and postsynaptic spikes throughout the simulation. The total analysis duration was segmented into 10 seconds-bins and each neuron's instantaneous firing rate was determined within smaller 10 ms sub-bins, identifying bursts when the firing rate exceeded 10 Hz. Instantaneous Network Firing (INF) for each 10 seconds-bin was determined by averaging individual neuronal burst rates across all recorded channels. The evolution of INF (left) and raster plots (right) illustrate the network's evolving activity patterns as synaptic weights are continuously modulated by STDP.

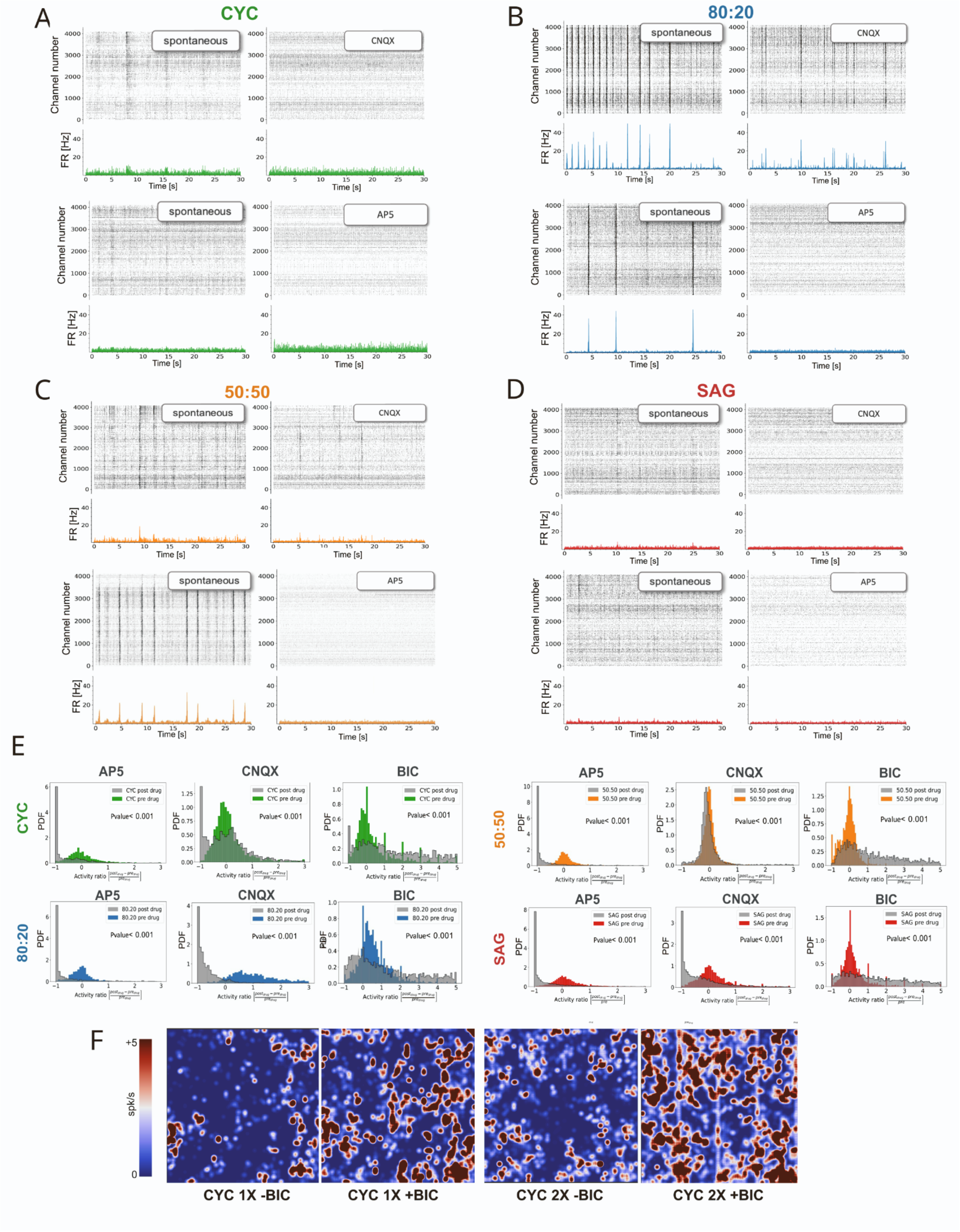

Figure S5. AP5, CNQX and Bicuculline significantly affect the firing activity in pure and mixed cultures. A-D) Representative raster plots for each culture condition showing the activity before and after drug administration (AP5 and CNQX). E) Probability distribution of the activity variation of each channel, before and after drug administration. The colored distributions represent the variation of baseline activity (without drugs) considering different time portions of spontaneous activity. The gray

distributions represent the variation after drug administration; Non-parametric Mann-Whitney U test between the baseline and post-drug distributions (p-values are displayed on the graph); PDF, probability density function. F) Heatmaps of global activity (5 minutes) of CYC cultures at normal (1X) or double (2X) cell density, before (-) and after (+) bicuculline (BIC) administration.

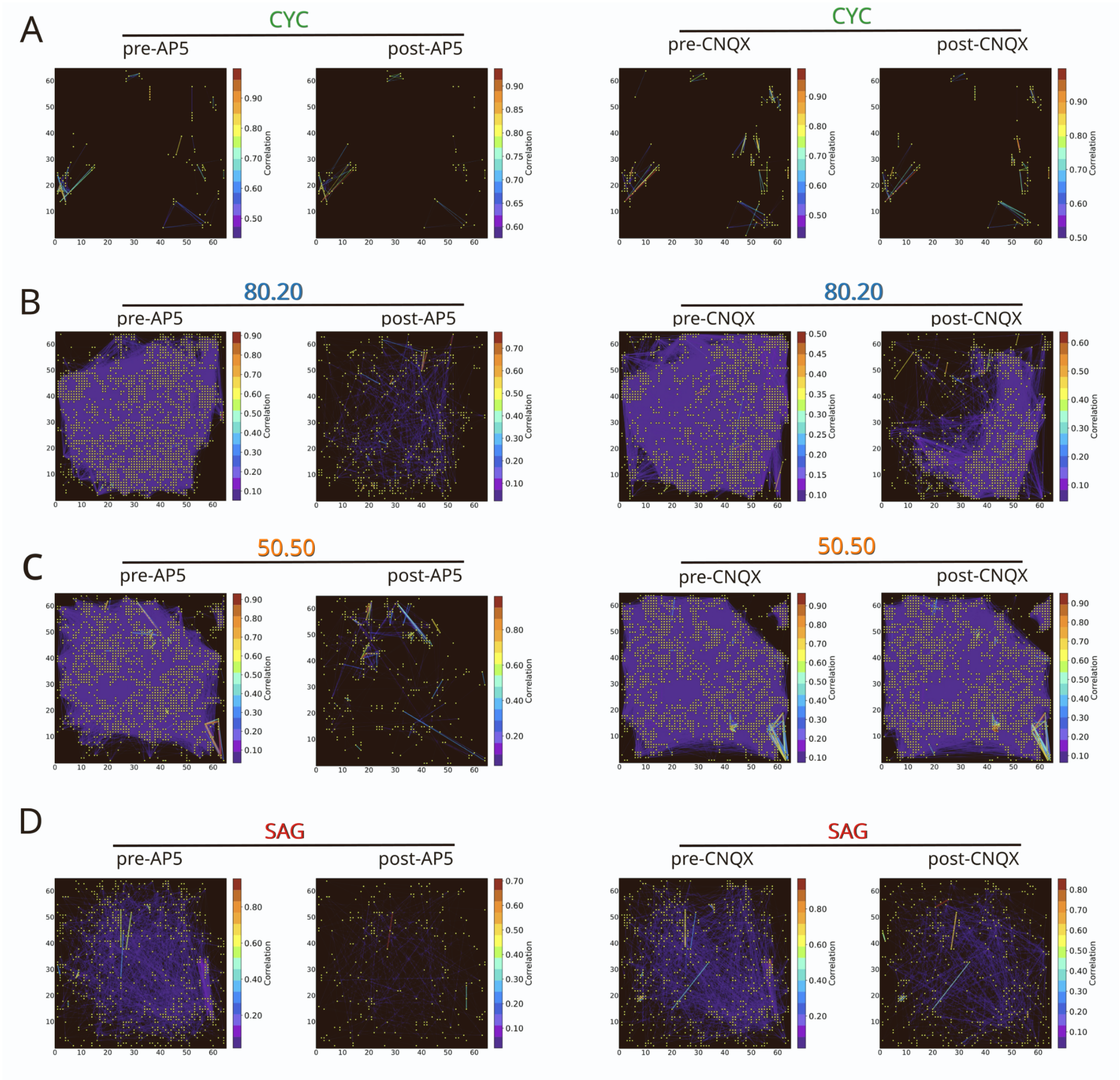

Figure S6. Representation of functional connectivity upon AP5 and CNQX administration. A-D) Connectivity plots of representative CYC (A), 80:20 (B), 50:50 (C) and SAG (D) cultures during spontaneous activity before (pre-drug) and after (post-drug) drug administration. Each yellow point represents a node of the functional graph; colored lines represent the correlation strength between two points (only the 10% of the functional links are shown). The color bar indicates the correlation index.

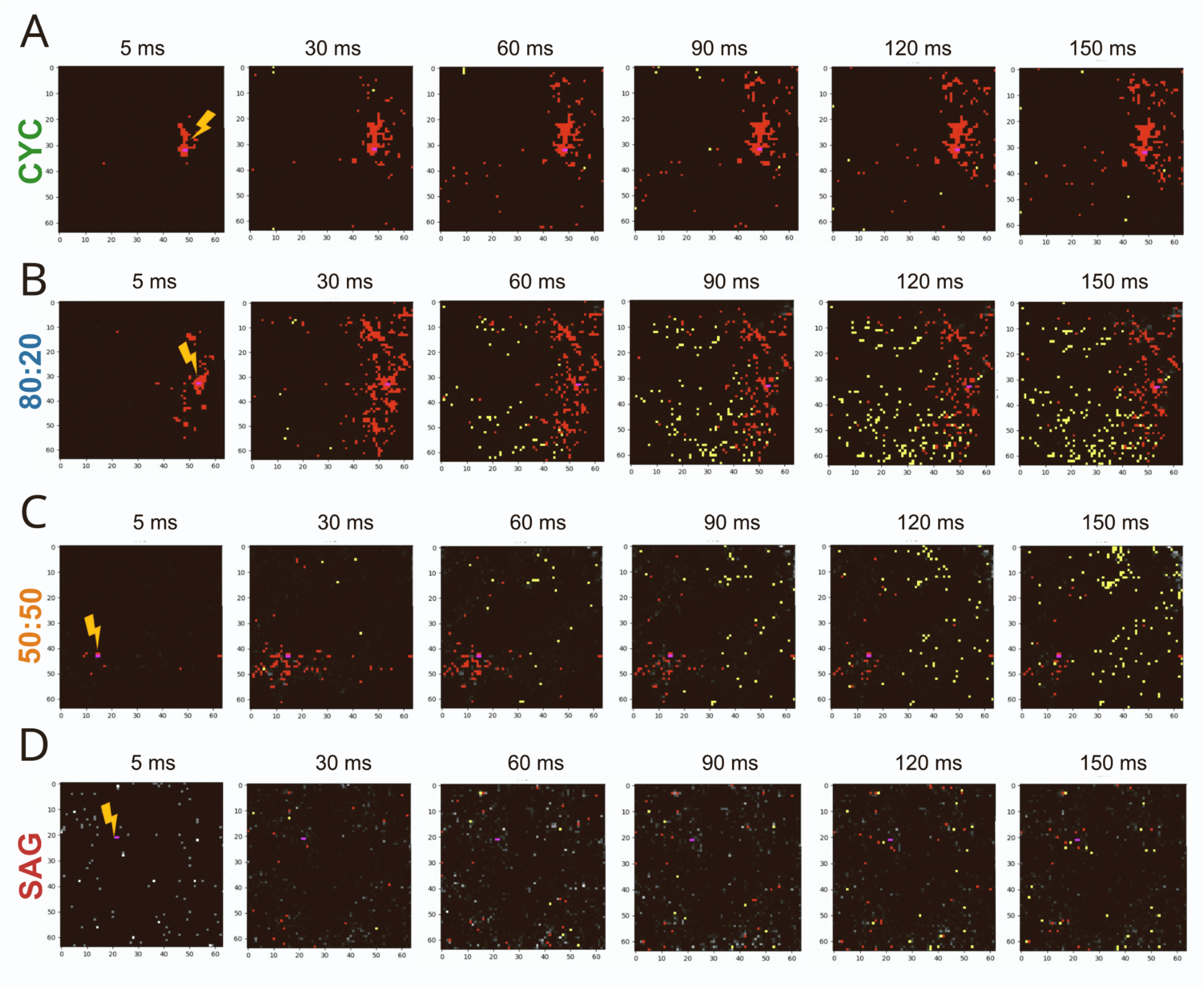

Figura S7. Schematic representation of single channel stimulation. A) Schematic representation of the chip plate, where each pixel represents a channel: purple pixels are two stimulated electrodes (cathode and anode), also indicated by the yellow lighting; white/grey pixels represent electrodes with baseline activity, red pixels represent channels that had a significantly increased modification as compared to the baseline activity (above the 95% CI), while yellow pixels are those with a significantly decreased modification as compared to the baseline activity (below 5% CI) (See Methods).

Supplemental information

Methods

Maintenance of mouse ES cells

Mouse embryonic stem cells (ES, clone E14Tg2A) were expanded and differentiated based on our previous method (Bertacchi et al., 2015) as follows. Cells were kept on 0,1% gelatin-coated culture dishes, seeding at a density of 50000 cells/cm^2 and splitting when at 70-80% of confluence. Cells were maintained in ES cell medium based on GMEM (Gibco, 11710035), containing 10% Fetal Bovine Serum (Euroclone ECS0180L), 2mM Glutamine, 1 mM Sodium Pyruvate, 100 U/ml Penicillin-streptomycin, 1mM Non-essential amino acids, 0.05mM β-mercaptoethanol. The medium was replaced daily.

ES cells were expanded in ES medium for three to four passages, then cultured in 2i+LIF medium, based on GMEM supplemented with 1x N-2 Supplement (Gibco, 17502001), 1x B-27 Supplement minus Vitamin A (Gibco, 12587010), 2mM Glutamine, 1mM Sodium Pyruvate, 1mM NEAA, 0.05mM β-mercaptoethanol, 1μM MEK inhibitor PD0325901 (Mirdametinib, Selleck Chemicals, S1036), 3μM GSK3 inhibitor CHIR99021 (Sigma-Aldrich, SML1046), and 10ng/mL recombinant mouse LIF (Silva et al., 2008).

Differentiation of ES cells to different cortical fates

Differentiation of ES cells into cortical neurons was performed as previously described (Bertacchi et al., 2015; Tonelli et al., 2025). For neural induction, a chemically defined minimal medium (CDMM) containing DMEM/F12 (Gibco, 11320033), 2mM Glutamine, 1mM Sodium Pyruvate, 0.1mM NEAA, 0.05mM β-mercaptoethanol, 1x N-2 Supplement, and 1x B-27 Supplement minus Vitamin A was used. The differentiation protocol started by culturing ES cells (3x10^4 cell/cm^2) onto 0,1% gelatin plastic coated dishes in 2i+LIF Medium for one day, marked as Day *in Vitro* -1 (DIV -1).

The next day (DIV0), the medium was replaced with Wnt and BMP double inhibition (WiBi) medium: CDMM with 2.5μM 53AH (Wnt pathway inhibitor, Cellagen Technology, C5324-2s) and 0.25μM LDN193189 hydrochloride (BMP inhibitor, Sigma-Aldrich, SML0559). Cells were cultured in WiBi medium for 3 days (DIV0 - DIV3). At DIV3, differentiating ES cells were dissociated and seeded (30000 cells/cm2) in CDMM on dishes coated with Poly-ornithine (PLO, Sigma; 4 μg/cm^2 in sterile water, 3 hours coating at 37°C) and purified mouse Laminin (msLam, Sigma-Aldrich, CC095-M; 1 μg/cm^2 in PBS, O/N coating at 37°C). Next day, the medium was changed to WiBi medium and cells were cultured until DIV5 with daily medium changes.

From DIV5 until DIV10 Cyclopamine (Sigma, S- 4116, 3μM) or Smoothened agonist SAG (Santa Cruz Biotechnology, SC-212905, 0.1μM) were added to WiBi medium to

differentiate the two neural populations. At DIV7 cells were split again onto mouse-Laminin coating dishes with a density of 110.000 cells/cm^2.
From DIV11 until DIV21 cells were maintained in “young” Neurobasal (yNb), containing Neurobasal medium, 2mM Glutamine, 1mM sodium Pyruvate, 0.05mM β-mercaptoethanol, 0.2mM Ascorbic Acid (Vit. C), and B-27 Supplement minus Vitamin A. From DIV13, half of the eNb medium was changed daily to allow conditioning of the medium by the differentiating neurons.
At DIV21, medium was changed to “old” Neurobasal medium (oNb), containing Neurobasal-A (Gibco, 10888022), 2mM Glutamine, 1mM sodium Pyruvate, 0.05mM β-mercaptoethanol, 0.2mM Ascorbic Acid (Vit. C), B-27 Supplement 50x (Gibco, 17504044), and 20 ng/ml recombinant human BDNF protein (Novus Biologicals, NBP2-52006). The oNb medium was changed every 2 to 3 days to condition the medium and allow the differentiated neurons to mature.

Passaging and long-term culture of neurons
Cells were passaged during differentiation to avoid overgrowth and hypoxia, last time at DIV22 for long term experiments. Cells were first washed with 1x Versene and then incubated with 1x Trypsin for 5-20 min at 37°C. When most of the cells detached, trypsin was inactivated by adding 20% FCS to the cell suspension and diluting in warm PBS (1:5 ratio).
The cell pellet was rinsed with a warm yNb medium containing 4μM of Rock inhibitor Y-27632 (Cell Guidance Systems, SM02) to reduce the mortality of post-mitotic neurons. Cells were seeded on PLO/msLam-treated glass (200000 cells/cm2) or sterilized high-density microelectrode array (HD-MEA Accura, 3Brain) (60000 cells/chip) and allowed to adhere O/N. The next day, the medium was changed, and the Rock inhibitor was removed. Occasionally, 1.5μg/mL msLam was added to the medium to increase neuron attachment and long-range projection.
To prepare the mixed cultures of 80:20 and 50:50, neurons were splitted at DIV22.

Lentiviral vectors transduction
Lentiviral vectors were prepared by transfecting HEK293T cells O/N with Lipofectamine 2000 (Invitrogen, 11668019) and a DNA mixture according to the manufacturer's protocols. The DNA mixture consisted of the lentiviral vector of interest together with the psPAX2 packaging (Addgene #12260), the pCMV-VSV-G envelope (Addgene #8454), and the pCMV-Rev(NL4.3) (Addgene #115776) expressing plasmids in a 4:3:1:1 ratio. Transfection medium was discarded the next morning, and viral particles were collected 48 hours later and used immediately or frozen at -80°C. To achieve a high rate of cell transduction, lentiviral vectors were used fresh in a 1:1 ratio with the culture medium along with 8μg/mL Polybrene (Sigma-Aldrich, TR-1003), and cells were typically transduced O/N after passaging to increase lentiviral access to the cell surface. The lentiviral vectors used in this work consisted of the pWPXLd lentiviral backbone (Addgene #12258) containing the EGFP coding sequence. To clone the EGFP reporter with PuroR sequence, the EGFP enhancer construct was amplified by PCR using a forward and reverse primer

carrying a MluI and EcoRI restriction site, respectively (forward: actacgggatccaggcctaagcttACGCGT; reverse: tagctagctactaGAATTCgagatctgagt). The vector carrying EGFP was constructed replacing the original EF1α promoter and PuroR sequence in the pLV-EF1α-IRES-Puro vector (Addgene #85132) with the amplicon carrying the EGFP reporter using the MluI/EcoRI restriction sites. The ligated vector was then sequenced to ensure correct cloning of the reporter.

Immunofluorescence (IF) analysis

All cell cultures were fixed with PFA solution (2%), incubated at RT for 9 minutes, followed by aspiration and 3 washes with 1XPBS at room temperature (RT). Cells were then permeabilized and blocked in 3%FCS, 3%BSA (Blocking Buffer solution) + 0.3% Triton at RT for 1 hour. The permeabilization buffer was then aspirated and replaced with a primary antibody solution in Blocking containing 0,1% Triton and the corresponding dilution in the table S1 below. Primary antibody solution was incubated at 4°C overnight. The next day, the primary antibody solution was removed, and cells were washed 3 times with 1XPBS at RT.

The secondary antibody solution was supplemented with corresponding anti-(host) secondary antibodies in the Blocking Buffer solution, diluted at 1:1000, and cells were incubated for 1 hour at RT. Secondary antibody solution was removed, and cells were washed 3 times with 1XPBS at RT. DAPI was added to the last 10' PBS wash, diluted 1:10.000. After the final PBS wash, all PBS was aspirated, and cells were mounted in Aqua/Poly-mount (Polysciences, 18606-100) for confocal imaging.

Images were produced on a Leica Stellaris 5 or Zeiss LSM 900 confocal microscope, by acquiring z-stack images 10-15 optical slices thick at 40x or 63x magnification.

Table S1. Primary,secondary antibodies and chemicals used in this section:

Antibody	Dilution	Host	Company	Catalog no
GFP	1:3000	Chicken	Aves	GFP-1020
Tubulinβ3	1:10000	Mouse	BioLegend	801202
PARVALBUMIN	1:1000	Guinea pig	Synaptic Systems	AB-2156476
SST	1:500	Rat	Millipore	MAB354
vGLUT2	1:1000	Rabbit	Synaptic Systems	135403
vGLUT1	1:1000	Rabbit	Invitrogen	482400
MAP2	1:6000	Chicken	Novus	NB300-213
vGAT	1:500	Guinea pig	Synaptic Systems	131011C2
GFAP	1:1000	Mouse	Sigma-Aldrich	MAB360

SATB2	1:200	Mouse	Santa Cruz Biotechnology	sc-81376
Tbr1	1:400	Guinea pig	Synaptic System	328005
Pax6	1:400	Rabbit	Covance	PRB-278P
Nkx2.1	1:400	Rabbit	Abcam	ab76013
FOXG1	1:500	Rabbit	Abcam	ab18259

**Antibody**	**Fluorophore**	**Company**	**Catalog no**
DONKEY<br>Anti-Mouse IgG	Alexa fluor 546	Invitrogen	A10036
GOAT Anti-Rabbit IgG	Alexa fluor 488	Abcam	AB150077
GOAT Anti-Chicken IgG	Alexa fluor 488	Invitrogen	A11039
GOAT Anti-Guinea Pig IgG	Alexa fluor 488	Invitrogen	A11073
GOAT Anti-Rabbit IgG	Alexa fluor 546	Invitrogen	A11035

**Chemicals**	**Company**	**Catalog no**
biotinylated Wisteria Floribunda Lectin (WFA)	Vector Laboratories	B-1355-2
Streptavidin, Alexa Fluor™ 488 conjugate	Thermo Fisher	S11223

Quantification of Neurite Branch Length and Spine Number Using SNT

Images were acquired with an HC PL APO 63x/1,40 OIL CS2 objective lens and quantification of neuronal morphology, including neurite branch length and spine number, was performed using the Simple Neurite Tracer (SNT) toolbox (v3.1.114), implemented within the Fiji distribution of ImageJ (Arshadi et al., 2021). Quantification of images was conducted under a double-blind protocol. Images were imported into Fiji and preprocessed as necessary to ensure optimal contrast for tracing (e.g., background subtraction, channel separation for multi-channel images). Neuronal processes were traced using SNT's semiautomated pathfinding algorithm. Centerlines of dendritic and axonal arbors were generated from intensity thresholded images, with tracing supported across multi-dimensional image stacks. After completion of the tracing procedure, quantitative morphometric analysis was carried out using SNT's built-in metrics. Individual branch segments were defined between bifurcation or termination points, and total cable length was computed per cell.

Spine detection and quantification were performed from EGFP-labeled fluorescent images for dendritic morphology (Miura et al., 2020). The analysis was performed observing the morphology of different types of spines according to the literature (Harris KM et al., 1992; Kuo et al., 2023). Spot density (number of spines) was computed for each traced segment and aggregated per cell. Tracing data were stored in SNT's traces format and exported in CSV format for compatibility with downstream analysis tools. Statistical comparisons between conditions were performed using ANOVA with Tukey's multiple comparisons test.

Quantification analyses of the density of glutamatergic and GABAergic vesicles

This analysis was performed using the ImageJ Synapse Counter plugin (https://github.com/SynPuCo/SynapseCounter). To determine the density of synaptic puncta per fiber, vGlut1, vGlut2, and vGat positive puncta were divided by the total area of the Tubβ3 fluorescent signal in each field (see Figure 2). Moreover, the counting of the percentage of vGlut1 and vGlut2 positive cells was calculated by dividing the percentage of nuclei surrounded by synaptic vesicles by the number of neurons (Tubβ3 positive cells) (see Figure S2) (Kempf et al., 2021).

For the analysis of cell clusters in CYC and SAG cultures at DIV30 and DIV50 (Figure S4), the number of DAPI positive cells was calculated using ImageJ's "Analyze Particles" feature for each image (confocal images taken with a 10x objective).

Quantification was performed on three independent experiments and on selected fields for each sample. Statistical significance was assessed using one-way ANOVA and Student's t-test followed by Tukey's multiple comparison test after testing for normality and lognormality. To compare the two time points, statistical significance was assessed using two-way ANOVA with Šídák's multiple comparisons test.

RNA extraction and qRT-PCR analysis

Samples for RNA extraction were harvested following the same protocol used for splitting. After the centrifugation step, the supernatant was removed, and the cell pellet was processed using the NucleoSpin® RNA kit (Machery-Nagel, 740955.250). RNA concentration was measured with the NanoDropTM Lite Spectrophotometer. For each RNA sample, approximately 200 ng of RNA were reverse transcribed into cDNA for qRT-PCR analysis using the Reverse Transcriptase Core Kit 300 (Eurogentec RT-RTCK-03). 8 μL of cDNA were then mixed with SensiFAST SYBR mix (12 ml, BioLine BIO-98020) and the amplification analyses were quantified with Qiagen 72- Well Rotorgene (Corbett).

The Relative Expression method supplied with the software of the Rotorgene device was employed. The CT for each gene was obtained directly from Rotorgene. An internal control was used to reduce the variability caused by possible changes in the amount of RNA/DNA between each sample, following the ΔCT analysis method (Pfaffl, 2001). β-actin was used as the reference gene. The PCR efficiency of each

sample was raised to the ∆CT to obtain the fold change of the target gene relative to the expression of β-actin (which expression was set to 1 with this method).

Table S2. qRT-PCR primer sequences.

GENE OF INTEREST	FORWARD PRIMER	REVERSE PRIMER
βActin	AATCGTGCGTGACATCAAAG	AAGGAAGGCTGGAAAAGAGC
FoxG1	CGACCCTGCCCTGTG	GGAAGAAGACCCCTG
Nkx2.1	CAATGAGGCTGACGC	GAAGTGGGTTTCCTG
VIP	GCACCAGCAGGCAGTAACAG	ACAAGGAGCTGGGCCTTATT
Sst	TCGCTCTAAGTCTCACTCGCC	CGCTCCCATATTTCAGCCAC
Pvalb	TCTTTTCGCACTTGCTCTGC	CCTTCTTCACCTCATCCGGG
Ascl1	GCCCGAATCACAGATGGGTT	ATCAACCCAGTTTCAGGGGG
Dlx2	TCCTACTCCGCCAAAAGCAG	GGAGTAGATGGTGCGTGGTT
Pax6	CCTCCTTCACATCAGGTTCC	CATAACTCCGCCCATTCACT
Lhx6	CGGCCTGATGGATCTCACTG	CTGGGCCATCACCTGCAAT
Lhx8	AAACACGTCAGTCCCAACCA	ACGTAGGCAGAATAAGCCATTT
Dlx1	GGTTTCTGGGGCGGGAAGCG	GGAGCGGGACGCACAATGGG
Sfrp1	CTGCCTCCTGCATGTGTGTA	TCTGGATGGGCTTTTCGCTT

Electrophysiological recordings and analysis

Neuronal cultures (DIV22) were seeded for recordings at a density of $5*10^4$ cells/MEA chip in all experiments, except control experiments with double density in which 10^5 cells/MEA chip were seeded. Cells were cultured onto commercially available Accura HD-MEA chips (3Brain GmbH), each equipped with 4096 CMOS microelectrodes with 60µm pitch and 21×21µm size, allowing recording of extracellular local field potentials. The 4096 electrodes are arranged in a 64×64 grid of 3.8 x 3.8 mm. Electrophysiological recordings were performed on DIV25-45 using the BioCam DupleX system (3Brain GmbH). After a 5 min acclimation period outside the incubator, spontaneous neuronal activity was recorded for 5 min under stable conditions (37°C, 5% CO_2) and sampled at 20kHz. Spike detection was performed using BrainWave 5 software (3Brain GmbH; see next paragraph). Finally, chemical stimulation was induced by adding specific compounds to the medium: Bicuculline (BIC, 20µM; Sigma-Aldrich, 14340) to block GABA receptors, D-2-amino-5-phosphonopentanoic acid (AP5, 25µM; Sigma-Aldrich, A8054) to block NMDA receptors, and 6-cyano-7-nitroquinoxaline-2,3-dione (CNQX, 25µM;

Sigma-Aldrich, C127) to block AMPA receptors. Electrophysiological activity was recorded 5 min before and 6 min after drug administration.

Protocol of stimulation

We stimulated all the types of cultures with electrical stimulation of one pair of electrodes (for single stimulation) or on seven pairs of electrodes (for multiple stimulations) that had the highest Firing Rate (>5 spks/sec). We used a biphasic stimulus with the following parameters: current amplitude of 10 µA per electrode, with a duration of 100 µs (50 % duty cycle) and an interphase delay of 10 µs. We applied a protocol of 25 stimuli at 0,1 Hz. To evaluate network-induced activity, we performed network burst (NB) detection and classified a network burst as electrically induced if the absolute difference between its start time and the stimulus time was less than twice the time bin used in the NB detection algorithm. Then we computed the temporal duration for the spontaneous and the induced NB.

Dispersion Index and Distribution of the global response to the stimulus

The results of dispersion index and global response were based on the computation of temporally aggregated neural activity in response to a given stimulus.

When a stimulation pulse was delivered, electrical activity was recorded from every other MEA electrode. A spike sequence was then extracted by means of a spike detection algorithm (described in the following subsection).

Some spurious spikes and electrical artifacts were removed by means of a filtering algorithm, removing spike signals with amplitude $|V|_{max}$ above a threshold $V_{thr} = 1000\,mV$. Another filtering algorithm calculates the area under the spike curve A and divides it by the spike amplitude in order to obtain an estimate of the spike temporal width, and filters out the spike when this value is above another threshold $W_{thr} = 25us$:

$$|V_{max} - V_{min}| > V_{thr}\ , \quad \frac{A}{|V_{max} - V_{min}|} > W_{thr}$$

The response to a stimulus observed on a given electrode is defined as the difference in spike count between a time window immediately after the stimulus instant, and one immediately preceding it. The window size W is chosen to range from 5 to 150 ms. Mathematically, the response r_i over electrode i (i=1, 2, …, 4096) can be expressed as:

$$r_i^{(t_s)} = \sum_{t=t_s}^{t_s+W} s_i(t) - \sum_{t=t_s-W}^{t_s} s_i(t)$$

Where t_s denotes the stimulus delivery time, and $s_i(t)$ is the spike signal recorded from electrode i, i.e. a function that takes value 1 when t corresponds to the time of a spike event, and 0 everywhere else.

During a recording session, stimulations were repeated multiple times, in order to account for statistical variability. Therefore, from the recordings, multiple values of the response variable over each electrode are obtained. Calling the stimulation

instants t_0, t_1, …, t_k, it is possible to obtain responses $r_i^{(t\_0)}$, $r_i^{(t\_1)}$, …, $r_i^{(t\_k)}$. We used k=25 in our experiments. Each of these values can be seen as a realization of a random variable R_i, of which we wish to provide a statistically grounded estimate. This is by computing the sample mean and the corresponding confidence intervals from the observations $r_i^{(t\_0)}$, $r_i^{(t\_1)}$, …, $r_i^{(t\_k)}$:

$$\hat{R}_i = \frac{1}{k} \sum_{s=0}^{k} r_i^{(t_s)}$$

Concerning the confidence intervals, initially we employed both a parametric and a nonparametric estimator. However, we found the non-parametric estimator to be ill suited for scenarios with very few spikes in the time window, so we resorted to the parametric estimator instead. This estimator is simply based on the T-Student estimation for the confidence intervals of the sample mean:

$$CI_\alpha^{+},\ CI_\alpha^{-} = \hat{R}_i \pm t^{k-1}_{1-\alpha/2} \frac{\sigma_i}{\sqrt{k}}$$

where σ_i is the sample standard deviation of the observations $r_i^{(t\_0)}$, $r_i^{(t\_1)}$, …, $r_i^{(t\_k)}$, and $t^{k-1}_{1-\alpha/2}$ is the required T-Student's percentile.

Each pixel of the matrices in Fig. S7 represent the corresponding value $\hat{R}_i$ measured for each electrode i, and represented graphically in grayscale: darker colors represent weaker responses, while brighter colors correspond to stronger responses. Moreover, when the measured confidence intervals indicate that the response value of a given pixel is significantly above zero in a statistical sense, then the corresponding pixel is colored in red. Specifically, the required confidence level is set to 95%. Similarly, when a confidence level indicates that the response of an electrode is significantly below zero, the pixel is denoted in yellow. In this case we require the positive tail of the distribution to be below zero at the 100 - 95 = 5 percentile.

Finally, the dispersion index in Fig. 7 is obtained by considering the geometric dispersion of the electrodes with significant response to a stimulus. Ideally, if all the response is concentrated in a very localized portion of the MEA grid, the corresponding dispersion should be low, while if the response is spread out all over the grid, the dispersion is large. More formally, the dispersion index is computed by considering the positions of the significantly responding electrodes over the grid, represented as x-y coordinates in the domain [0, 1]x[0, 1] (where coordinates 0, 0 denote the top-left corner, and 1-1 denote the bottom right corner of the MEA grid).

Let's denote with (x_i, y_i) the coordinates of electrode i, and let R = {i_1, i_2, …} be the set of significantly responsive electrodes. It is possible to evaluate the distance between any pair of such electrodes as

$$d_{a,b} = \sqrt{(x_a - x_b)^2 + (y_a - y_b)^2}$$

This can be again considered as a realization of a random variable D, which depends on the particular pair a, b that was chosen. It is also possible to obtain

several samples of D by selecting all possible electrode pairs from R. From all these samples, we can once more evaluate the sample mean D, and the corresponding confidence intervals, for statistical comparisons.

$$\hat{D} = \frac{1}{k} \sum_{a,b} d_{a,b}$$

$$CI_{\alpha}^{+}, CI_{\alpha}^{-} = \hat{D}_i \pm t^{k-1}_{1-\alpha/2} \frac{\sigma}{\sqrt{k}}$$

In this case, k=$|R|(|R| - 1)/2$, and σ is the sample standard deviation of the observations $d_{a,b}$.

The parametric estimation methods discussed above are based on the central limit theorem, and rely on the assumption of variance finiteness in the random variables being statistically estimated. This assumption was checked by observing the homoscedasticity of the observations, i.e. sample variance converging stably as the number of observations increased.

Spike and Burst detection

The spike detection algorithm used is the PTSD (precision time spike detection) (Maccione et al., 2009) that requires 3 parameters:

- Noise threshold (set to 10 times the standard deviation of the baseline noise for each channel individually).
- Peak lifetime period (set to 2 ms), corresponding approximately to the spike duration.
- Refractory period (set to 2 ms), which corresponds to the minimum time interval between one spike and the next (Parodi et al., 2023).

After spike detection, each channel burst was detected based on the channel spike train. The burst detection was performed as proposed in the literature (Chiappalone et al., 2005). The implemented algorithm requires 2 parameters: the maximum inter-spike interval (ISI) between two consecutive spikes of a burst (*maxISI*, set to 50 ms) and the minimum number of spikes in a burst (*minspk*, set to 5 spikes).

Spike bursts are defined as sequences of spikes with ISI smaller than *maxISI* and containing at least a number of spikes equal to *minspk*. The values of 50 ms and 5 spikes for these two parameters were set after a series of comparisons between the results of the burst detection algorithm and visual inspection of various experimental recordings. Mean firing rate (MFR) and mean burst rate (MBR) were calculated by counting the average number of spikes or bursts in 5 minute recordings divided by the number of active channels (firing rate greater than 0.1 spikes/s for spiking activity and burst rate greater than 0.3 bursts/min for bursting activity). To fully characterize the bursting activity, the mean burst duration (MBD), the percentage of bursting electrodes and the percentage of random spikes were calculated. The mean burst duration (MBD) was calculated as the average temporal length of the bursts. The percentage of bursting electrodes was determined as the proportion of active channels exhibiting bursting activity. Lastly, the percentage of random spikes was computed by considering all spikes that were not part of any burst activity. All these

quantities were calculated for all replicates of the same culture and the Mean ± SEM was plotted over time (Figure 4).

Network Burst activity

Network bursts (NBs) are events of collective synchronization within the culture. To quantify the level of synchronization of the neuronal network activity, we derived the mean network burst rate (NBR, number of network events per minute) and the network burst duration (NBD) for each culture. A NB is identified when the activity is composed of at least 50 consecutive spikes within a 50 ms window and the firing rate (number of spikes per bin) exceeds a threshold determined from the mean and standard deviation of the firing rate signal. NB detection was performed as follows:

- The network's firing rate (number of spikes per bin) was calculated using a time bin of 50 ms.
- Firing rate peaks were considered if the local firing rate maxima were greater than the mean of the firing rate signal plus 4 times the standard deviation.
- The beginning and end of each NB were defined such that the firing rate before and after each peak fell below the mean of the firing rate signal plus 2 times the standard deviation (in cases where two or more adjacent local maxima correspond to the same onset, only one event is detected).

NBR was calculated for all replicates of the same culture and the Mean ± SEM was plotted over time.

Center of Activity Trajectories (CATs)

To quantify the propagation of coordinated network activity, we performed Center of Activity Trajectory (CAT) (Chao et al., 2007) analysis, which calculates the spatial and temporal evolution of each NB event. CAT provides a sort of center of mass for spikes, where the location of the center of mass is the physical location of the channels in the MEA map, and mass is replaced by spike activity.

The algorithm to compute NB trajectories is as follows: firstly, the start and end points of a NB are detected (as explained in the previous section) to define the time window of interest. All active channel spikes are then considered to compute the neuronal activity trajectory in this time window. A time step $\delta_t = 20$ ms, is fixed for the binning of neuronal firing. Then, the activity is computed by counting all spikes for each channel in each time window $[t, t + \delta_t]$. Finally, the activity trajectory is defined by : $CA(t) = \frac{\sum_{ch} A_{ch}(t)(row_{ch}, col_{ch})}{\sum_{ch} A_{ch}(t)}$, where $A_{ch}(t)$ represents the activity (counted spikes) of the channel ch at time t (in the corresponding time window $[t, t + \delta_t]$) and (row_{ch}, col_{ch}) are the physical coordinates in the MEA map (row and column) of the channel ch.

For each NB, we computed the spatial and temporal evolution of its activity trajectory throughout the duration of the synchronized event. The time evolution (with a time window of 150 ms for each NB) is represented in the plots by a colored scale, while the onset of each NB is highlighted by a blue dot.

Network connectivity analysis

To estimate the functional connectivity within the neural networks, we used a previously validated cross-correlation-based approach (Ullo et al., 2014), dealing with point processes of events (e.g., spike trains). More precisely, for each pair of active electrodes $\{x,\ y\}$, the cross-correlation function (cross-correlogram) between their spike trains was estimated as:

$$C_{x,y}(\tau) = \frac{1}{\sqrt{N_x N_y}} \sum_{s=1}^{N_x} \sum_{t=t_s-\frac{\Delta_t}{2}}^{t_s+\frac{\Delta_t}{2}} x(t_s) y(t + \tau)$$

where N_x, N_y are the total number of spikes for channels x and y respectively. The time bin Δ_t was set to 1 ms and the time delay tau (τ) was varied in a range from -2 to 2 ms. The absence of delay (τ = 0 ms) was excluded to eliminate synchronous spikes less than 1 ms apart, which are incompatible with the synaptic time delay (Poli et al., 2015). For each pair of channels, the maximum correlation value at delay τ was considered. To determine which correlations are statistically significant, it is necessary to select a threshold. We implemented a shuffling procedure that randomizes the temporal order of events to create a null hypothesis scenario where all observed correlations are purely due to chance as described in Tonelli et al., 2025.

The connectivity matrix of the randomized spike trains is computed, and the threshold is determined by computing the 99.9th percentile of the random correlation distribution. From the connectivity matrix, we derived the adjacency matrix, where the strength of the connections for the graph is represented by the correlation values (Figure 6A). Connectivity graphs were plotted while maintaining the physical position of the channels in the MEA map, with each connection colored based on the correlation value. From these graphs, we extracted the number of nodes and links (Figure 6).

Characterization of neuronal activity after chemical drug administration

To evaluate the effect of chemical drugs on the five different cultures, we computed the probability distributions of single channel activity variation before and after drug administration. For each culture, we computed the number of spikes per channel, considering all active channels under normal and drug conditions. The drug condition was considered excluding the first 100 seconds of recording after the moment of administration. The activity variation was calculated as the differences between the channel activity after and before drug administration (number of spikes per channel), divided for the channel activity before administration. To evaluate the real effect of the drugs, for each replicate of the same culture, we calculated the variation of the baseline activity in normal condition, considering different time portions of spontaneous activity (excluding 100 seconds of recordings between one portion and another). The distributions of firing rate variation account for the activity of individual

channels across all replicates. Finally, a non-parametric Mann-Whitney U test was performed to determine statistically significant differences between the baseline and post-drug distributions. Furthermore, to quantify the changes in the functional connectivity before and after drug administration, we computed the variation of the number of nodes and links compared to the baseline conditions (Figure 6C).

Neural network simulation

We implemented a published neuronal network model that establishes random connectivity with distinct populations of excitatory and inhibitory neurons, explicitly modeling axonal conduction delays and implementing Spike-Timing Dependent Plasticity (STDP) (Izhikevich, 2006). Neural network simulation was performed in the Matlab environment. The neural network was constructed with a total of 1000 neurons, varying the number of inhibitory cells, from 100 to 900. The dynamic behavior of each individual neuron was modeled using the Izhikevich formalism. For excitatory neurons, the recovery variable time scale was set at 0.02, with an after-spike reset value for the recovery variable of 8. Inhibitory neurons, in contrast, were characterized by a faster recovery variable time scale of 0.1 and a smaller after-spike reset value of 2 for the recovery variable. All neurons were initialized with a membrane potential of −65 mV, and their recovery variable was set to 0.2 times this initial membrane potential. A uniform spike threshold of 30 mV was applied across all neurons.

To stick to the Izhikevich formalism, network connectivity was established such that each neuron formed 100 synaptic connections with randomly selected target neurons. Synaptic weights were initialized differently based on the presynaptic neuron type: excitatory synapses began with a weight of 6, while inhibitory synapses were set to a fixed weight of −5. Importantly, excitatory synaptic weights were subject to a maximum constraint of 10 and a minimum of 0, and their values evolved over time through a mechanism of Spike-Timing Dependent Plasticity (STDP). In contrast, inhibitory synaptic weights remained constant throughout the simulation. To introduce biological realism, axonal conduction delays were incorporated into the model. Excitatory connections were assigned delays randomly drawn from a uniform distribution spanning 1 to 20 milliseconds. All inhibitory connections, reflecting their typically faster local influence, were assigned a fixed conduction delay of 1 millisecond. These values differ from those used by biological networks because in Izhikevich's formalism they proved optimal for modelling network activity.

The network received a continuous stochastic external drive, modeled as a random thalamic-like input. At each millisecond of the simulation, a single neuron was randomly selected to receive an excitatory current of 20 units, ensuring persistent but non-targeted external stimulation. This served to stimulate background activity within the network. The core of the network's learning capability lay in its implementation of STDP, which modulated excitatory synaptic strengths based on the precise relative timing of pre- and postsynaptic spikes. Upon a neuron's firing, its STDP trace was immediately incremented by 0.1, with this trace decaying

exponentially by a factor of 0.95 per millisecond between subsequent spikes. The synaptic weight derivatives accumulated based on these STDP traces, incorporating a scaling factor of −1.2 for presynaptic effects. At the completion of each simulated second, the excitatory synaptic weights were updated following the rule: se(t+1)=max(0,min(10,0.01+se(t)+sd(t))), where 0.01 was a small constant preventing excessive weight decay. Concurrently, the accumulated weight derivatives themselves decayed by a factor of 0.9 at the end of each second.

The simulation was carried out for a total duration of 15 minutes (900 seconds). The Izhikevich neuron model equations were numerically integrated using an Euler method, effectively employing a 0.5 ms time step for the membrane potential update, applied twice per millisecond simulation step. To ensure reproducibility of the simulation results, a specific random seed was set at the outset of the script. All occurrences of neural spiking, including the time of the spike and the identity of the firing neuron, were meticulously recorded and subsequently stored in a .mat file for comprehensive post-simulation analysis.

Network activity analysis was performed by analyzing spikes across multiple channels, with spikes binned into a predefined time interval of 10s, to estimate firing rates. Since the Izhikevich network does not generate burst dynamics with metrics directly comparable to biological networks, we employed an alternative metric that best approximates the average bursting activity observed in biological systems. We calculated Instantaneous Network Firing (INF). The burst rate for each neuron was quantified by first segmenting the activity within 10-second analysis bins into smaller, 10-millisecond sub-bins. Within these sub-bins, the instantaneous firing rate for individual neurons was computed. Bursts were then identified as discrete periods where the instantaneous firing rate exceeded a threshold of 10 Hz. The number of such burst events per second was subsequently calculated for each neuron. Finally, INF for each 10-second bin was determined by averaging these individual neuronal burst rates across all recorded channels.

Quantification and statistical data analysis

Unless otherwise stated, the data presented herein were analyzed with: GraphPad Prism software was used for statistical analysis and data plotting; Proprietary Leica and Zeiss confocal software was used for IF imaging, while Fiji software (imageJ) was used for downstream analysis; BrainWave 5 software and custom codes developed in Python were used to analyze the raw recording data of neuronal activity on the HD-MEA.

SUPPLEMENTAL REFERENCES

Arshadi, C., Günther, U., Eddison, M., Harrington, K.I.S., and Ferreira, T.A. (2021). SNT: a unifying toolbox for quantification of neuronal anatomy. Nat Methods *18*, 374–377. https://doi.org/10.1038/s41592-021-01105-7.

Bertacchi, M., Pandolfini, L., D'Onofrio, M., Brandi, R., and Cremisi, F. (2015). The double inhibition of endogenously produced BMP and W nt factors synergistically triggers dorsal telencephalic differentiation of mouse ES cells. Developmental Neurobiology *75*, 66–79. https://doi.org/10.1002/dneu.22209.

Chao, Z.C., Bakkum, D.J., and Potter, S.M. (2007). Region-specific network plasticity in simulated and living cortical networks: comparison of the center of activity trajectory (CAT) with other statistics. J. Neural Eng. *4*, 294–308. https://doi.org/10.1088/1741-2560/4/3/015.

Chiappalone, M., Novellino, A., Vajda, I., Vato, A., Martinoia, S., and Van Pelt, J. (2005). Burst detection algorithms for the analysis of spatio-temporal patterns in cortical networks of neurons. Neurocomputing *65–66*, 653–662. https://doi.org/10.1016/j.neucom.2004.10.094.

Harris KM, Jensen FE, and Tsao B (1992). Three-dimensional structure of dendritic spines and synapses in rat hippocampus (CA1) at postnatal day 15 and adult ages: implications for the maturation of synaptic physiology and long-term potentiation. Journal of Neuroscience 12 (7) 2685-2705. https://doi.org/10.1523/JNEUROSCI.12-07-02685.1992.

Izhikevich, E.M. (2006). Polychronization: Computation with Spikes. Neural Computation *18*, 245–282. https://doi.org/10.1162/089976606775093882.

Kempf, J., Knelles, K., Hersbach, B.A., Petrik, D., Riedemann, T., Bednarova, V., Janjic, A., Simon-Ebert, T., Enard, W., Smialowski, P., et al. (2021). Heterogeneity of neurons reprogrammed from spinal cord astrocytes by the proneural factors Ascl1 and Neurogenin2. Cell Reports *36*, 109409. https://doi.org/10.1016/j.celrep.2021.109409.

Kuo, H.-Y., Yang, Y.-H., Chen, S.-Y., Kuo, T.-H., Lin, W.-T., and Liu, F.-C. (2023). Differential Development of Dendritic Spines in Striatal Projection Neurons of Direct and Indirect Pathways in the Caudoputamen and Nucleus Accumbens. eNeuro *10*, ENEURO.0366-22.2023. https://doi.org/10.1523/ENEURO.0366-22.2023.

Maccione, A., Gandolfo, M., Massobrio, P., Novellino, A., Martinoia, S., and Chiappalone, M. (2009). A novel algorithm for precise identification of spikes in extracellularly recorded neuronal signals. Journal of Neuroscience Methods *177*, 241–249. https://doi.org/10.1016/j.jneumeth.2008.09.026.

Miura, Y., Li, M.-Y., Birey, F., Ikeda, K., Revah, O., Thete, M.V., Park, J.-Y., Puno, A., Lee, S.H., Porteus, M.H., et al. (2020). Generation of human striatal organoids and cortico-striatal assembloids from human pluripotent stem cells. Nature Biotechnology *38*, 1421–1430. https://doi.org/10.1038/s41587-020-00763-w.

Parodi, G., Brofiga, M., Pastore, V.P., Chiappalone, M., and Martinoia, S. (2023). Deepening the role of excitation/inhibition balance in human iPSCs-derived neuronal networks coupled to MEAs during long-term development. J. Neural Eng. *20*, 056011. https://doi.org/10.1088/1741-2552/acf78b.

Pfaffl, M.W. (2001). A new mathematical model for relative quantification in real-time RT-PCR. Nucleic Acids Research *29*, 45e–445. https://doi.org/10.1093/nar/29.9.e45.

Poli, D., Pastore, V.P., and Massobrio, P. (2015). Functional connectivity in in vitro neuronal assemblies. Front. Neural Circuits *9*. https://doi.org/10.3389/fncir.2015.00057.

Silva, J., Barrandon, O., Nichols, J., Kawaguchi, J., Theunissen, T.W., and Smith, A. (2008). Promotion of Reprogramming to Ground State Pluripotency by Signal Inhibition. PLoS Biol *6*, e253. https://doi.org/10.1371/journal.pbio.0060253.

Tonelli, F., Iannello, L., Gustincich, S., Di Garbo, A., Pandolfini, L., and Cremisi, F. (2025). Dual inhibition of MAPK/ERK and BMP signaling induces entorhinal-like identity in mouse ESC-derived pallial progenitors. Stem Cell Reports 102387. https://doi.org/10.1016/j.stemcr.2024.12.002.

Ullo, S., Nieus, T.R., Sona, D., Maccione, A., Berdondini, L., and Murino, V. (2014). Functional connectivity estimation over large networks at cellular resolution based on electrophysiological recordings and structural prior. Front. Neuroanat. *8*. https://doi.org/10.3389/fnana.2014.00137.
