# Supplementary material for: A proper excitatory/inhibitory ratio is required to develop synchronized network activity in mouse cortical cultures
Source: Stem Cell Reports. 2025 Sep 25;20(10):102646. doi: 10.1016/j.stemcr.2025.102646 (PMC12790722; doi:10.1016/j.stemcr.2025.102646)
Supplement: Document S2. Article plus supplemental information [file mmc7.pdf]

Stem Cell Reports
Article
OPEN ACCESS

A proper excitatory/inhibitory ratio is required to develop synchronized network activity in mouse cortical cultures

Eleonora Crocco,[1] Ludovico Iannello,[2] Fabrizio Tonelli,[1] Gabriele Lagani,[2] Luca Pandolfini,[3] Marcello Ferro,[4] Giuseppe Amato,[2] Angelo Di Garbo,[5,6] and Federico Cremisi[1,5,7,*]
[1]Laboratorio di Biologia Bio@SNS, Scuola Normale Superiore, Pisa, Italy
[2]Institute of Information Science and Technologies (ISTI-CNR), Pisa, Italy
[3]Center for Human Technologies, Central RNA Lab, Istituto Italiano di Tecnologia, 16152 Genova, Italy
[4]Istituto di Linguistica Computazionale "Antonio Zampolli" (CNR-ILC), Pisa, Italy
[5]Istituto di Biofisica, Consiglio Nazionale delle Ricerche, 56124 Pisa, Italy
[6]Dipartimento di Fisica, Università di Pisa, 56127 Pisa, Italy
[7]Lead contact
*Correspondence: federico.cremisi@sns.it
https://doi.org/10.1016/j.stemcr.2025.102646

SUMMARY

Excitatory/inhibitory (E/I) balance is thought to play a key role in cortical activity development. We modeled an *in vitro* cortical network deployed of the inhibitory neurons normally migrating from the ventral telencephalon and implemented ventral telencephalic (VT) cultures and co-cultures with mixed proportions of dorsal telencephalic (DT) and VT neurons, containing distinct proportions of inhibitory neurons. Interestingly, these pure and mixed cultures developed different patterns of spontaneous activity and functional connectivity. Our findings highlighted a critical role for the inhibitory component in developing correlated network activity. Unexpectedly, networks with 7% of parvalbumin (PV)$^+$ neurons were not able to generate appreciable network burst activity due to the development of a strong network inhibition, despite their lowest E/I ratio. Our observations support the notion that an optimal ratio of PV$^+$ neurons during cortical development is essential for the establishment of local inhibitory networks capable of generating and spreading correlated activity.

INTRODUCTION

Mouse embryonic stem cells (mESCs) or human induced pluripotent cells (hiPSCs) are key tools for modeling the development of distinct encephalic regions *in vitro* by controlling different signaling pathways in precise time windows (Chambers et al., 2009; Chiaradia and Lancaster, 2020). Forebrain identity is acquired by default and retained primarily through BMP and Wnt inhibition (Bertacchi et al., 2015; Watanabe et al., 2005). Dorsal telencephalic (DT) progenitors are generated *in vitro* using the sonic Hedgehog (Shh) inhibitor, cyclopamine (Gaspard et al., 2008), while ventral telencephalic (VT) progenitors require Shh activation by the Shh agonist, SAG (Cederquist et al., 2019; Li et al., 2009). *In vivo*, the mature cerebral cortex forms after VT cells migrate, mainly from the medial ganglionic eminence (MGE) (Wonders and Anderson, 2006). These cells differentiate into GABAergic inhibitory interneurons that connect with glutamatergic excitatory neurons generated by local DT progenitors, establishing a balanced Excitatory/Inhibitory(E/I) ratio (Gelman and Marín, 2010; Lodato et al., 2011). Notably, an unbalanced E/I ratio is linked to brain disorders such as schizophrenia or autism spectrum disorders (Nelson and Valakh, 2015; Sohal and Rubenstein, 2019).

To date, relatively few studies have reconstructed cortical circuits using defined ratios of VT and DT neurons (Mossink et al., 2022; Parodi et al., 2023, 2024). These studies used induced neurons (iNeurons) that only partially mimic natural telencephalic neurons. Specifically, *Ngn2*-induced neurons comprise a heterogeneous population displaying features of both central and peripheral nervous system lineages (Lin et al., 2021), while *Ascl1*-induced neurons show limited differentiation into parvalbumin-positive interneurons, which represent the predominant class of cortical inhibitory neurons *in vivo* (Zhang et al., 2013). To investigate more physiological neural networks with different E/I ratios and functionally characterize them during developmental maturation, we modeled two distinct populations of cells *in vitro*, DT and VT progenitors obtained by modulating the Shh pathway in mESC-derived telencephalic cells. We analyzed the activity of a network of DT progenitors alone, comparing it to a network of VT progenitors and to networks with varying ratios of DT and VT cells. Finally, we analyzed basic structural and functional network parameters such as synapse density, firing activity, network burst synchronization and connectivity, together with the ability to respond to electrical stimulations. Our findings indicate that the E/I balance dramatically affects the development and maturation of neuronal cultures, highlighting that a proper E/I ratio is required for the formation and spreading of correlated activity in cortical networks.

© 2025 The Authors.
Published by Elsevier Inc. on behalf of International Society for Stem Cell Research.
This is an open access article under the CC BY license (http://creativecommons.org/licenses/by/4.0/).

RESULTS

Cyclopamine and SAG treatments respectively induce the production of DT and VT neurons

During the first week of mESC neuralization, early double inhibition of Bone Morphogenetic Protein (BMP) and Wnt signaling (WiBi) induces a general telencephalic identity (Terrigno et al., 2018) (Figures 1A and 1B). From *in vitro* differentiation day (DIV)5 to DIV10, we added cyclopamine (3 μM) to WiBi-treated mESC to induce DT identity (Chen et al., 2002a) and smoothened agonist (SAG) (0.1 μM) to induce VT identity (Chen et al., 2002b). At DIV11, we confirmed the positional cell identity using early markers of telencephalic and subpallial identity (Fuccillo et al., 2006; Martynoga et al., 2005) (Figures 1B–1E and S1A–S1C). CYC cells expressed high levels of the dorsal marker *Pax6*, while SAG cells exhibited high expression of the ventral identity marker *Nkx2.1*. Moreover, both types of cells expressed high levels of the telencephalic marker *FoxG1* compared to control neuralized cells without Wnt inhibition (midbrain). We proceeded to analyze specific early markers of the subpallium and medial and lateral ganglionic eminences (MGE-LGE), such as *Lhx6*, *Lhx8*, *and Dlx1* (Chen et al., 2017) and *Ascl1*, *Dlx2*, *and Sfrp1* (Nery et al., 2002). We found an increased expression of these genes in SAG cells compared to the other neural population (Figures 1F and S1D–S1I).

Analysis of marker expression at the cellular level revealed that a high percentage of both CYC and SAG cells were positive for Foxg1 (Figures 1G and 1L). We observed that Nkx2.1 expression was largely restricted to SAG cells and was nearly absent in CYC cells (Figures 1H and 1L), while the dorsal markers Pax6 and Tbr1 were expressed in a large proportion of CYC cells but were barely detectable in SAG cells (Figures 1I, 1J, and 1L). By DIV20 (approximately equivalent to postnatal day P0 in mice), the cortical marker Satb2 was found in a significant fraction of CYC cells but was almost absent in SAG cells (Figures 1K and 1L). These data confirmed that SAG cultures have a ventral identity similar to the MGE and subpallium.

Since the MGE is the source of a wide variety of cortical interneurons (Gelman et al., 2011), we evaluated specific subtype markers such as parvalbumin (PV) and somatostatin (SST) (Figures 2A and 2B) (Lim et al., 2018). At DIV35, we counted 73% PV$^+$ neurons in SAG cultures, while we found a much lower percentage (7%) in CYC cultures (Figure 2A). SST$^+$ neurons accounted for less than 10% in SAG cultures and were virtually absent in CYC cultures (Figure 2B). Vasoactive intestinal peptide (VIP) neurons were not detected in either CYC or SAG cultures, suggesting that both culture conditions may lack progenitors from the caudal ganglionic eminence, where VIP$^+$ neurons are predominantly generated (Marín, 2012). Given the association of PV$^+$ interneurons with perineuronal nets (PNNs) (Lupori et al., 2023), we investigated also the reactivity against Wisteria floribunda agglutinin (WFA), a common marker for PNNs. A typical stained morphology of PNNs (Dickens et al., 2022), with this protein occasionally surrounding few PV$^+$ cells (Figure 2C), was observed as early as DIV50, suggesting the formation of some early PNNs, mostly in SAG cultures.

The immunolabeling of distinct markers of excitatory (vGlut1 and vGlut2) and inhibitory (Pvalb and vGat) cortical neurons showed a different contribution of the two types of cells in SAG and CYC cultures, confirming the prevalence of excitatory and inhibitory neurons in CYC and SAG cultures, respectively (Figure 2D). We investigated the early glutamatergic marker vGlut2 at DIV25: we evaluated the density of vesicles by observing the colocalization of vGlut2 puncta with Tubβ3$^+$ fibers and found a significantly higher density of vGlut2$^+$ vesicles in CYC neurons as compared to the SAG neurons (Figure 2E). Moreover, by counting the number of DAPI-positive nuclei surrounded by vesicle transporters, we evaluated 70% of vGlut2$^+$ cells in CYC cultures (Cao et al., 2017), while only 15% of them were present in SAG cultures (Kempf et al., 2021) (Figures S2A and S2B). We also analyzed the later glutamatergic marker vGlut1 and found a significantly higher density of vGlut1-positive puncta in CYC neurons as compared to SAG neurons (Figure 2E), with 80% of CYC neurons surrounded by VGlut1$^+$ vesicles (Figures S2C and S2D). Focusing on GABAergic markers, we analyzed the vGat density at DIV35 in our cultures, highlighting a significantly higher number of vGat$^+$ vesicles in SAG neurons as compared to CYC neurons (Figure 2E).

To assess the percentage of astrocytes in CYC and SAG cultures, we evaluated the presence of GFAP$^+$ cells at two time points of maturation, DIV21 and DIV30. At DIV21, SAG cultures had a higher percentage of GFAP$^+$ cells than CYC cultures, suggesting that they complete neurogenesis and start astrogliogenesis earlier. However, by DIV30, both cultures reached the same percentage (40%) of GFAP$^+$ cells (Figures 2F and 2G), indicating that cell differentiation was completed.

Altogether, our data indicate that timely treatment with either CYC or SAG from DIV5 to DIV11 generated pallial neural progenitor cells (NPCs) with the competence to differentiate into neurons with gene expression profiles typical of DT and VT neurons, respectively. CYC cells produced mostly glutamatergic pyramidal neurons while SAG cultures were enriched in GABAergic cells, mainly PV$^+$ interneurons, similar to those migrating to the embryonic developing cortex. Both cultures also produced an optimal ratio of astrocytes (40%), which is expected to support functional network activity.

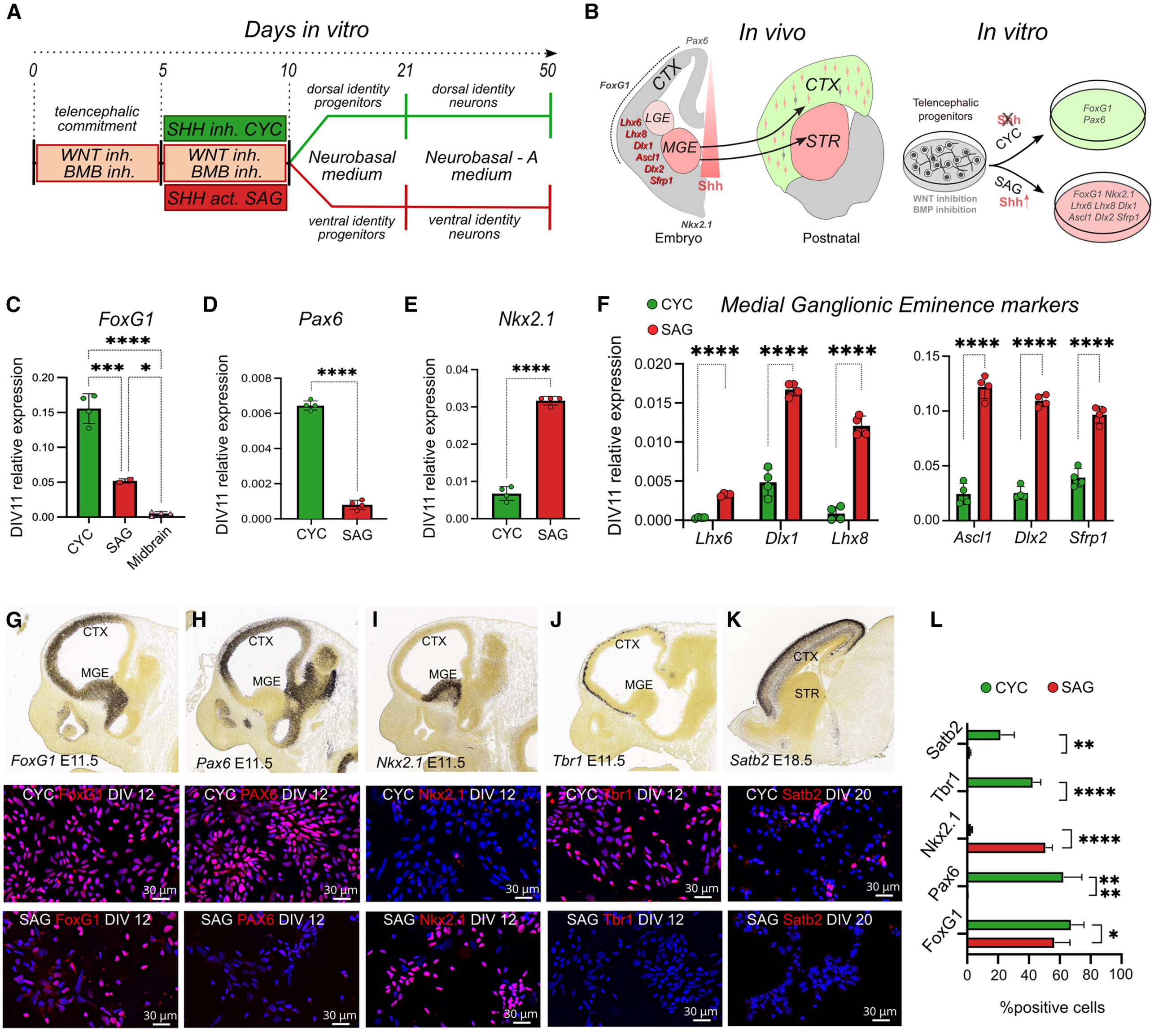

Figure 1. Positional identity and characterization of CYC and SAG progenitor cells

(A) Protocol of mESC neuralization.

(B) Schematic representation of marker expression *in vivo* and *in vitro*. CTX, cortex; STR, striatum; MGE, medial ganglionic eminence; LGE, lateral ganglionic eminence.

(C–E) Relative expression of early pallial markers by quantitative reverse-transcription PCR (RT-qPCR) ($n = 4$ independent experiments). Midbrain in (C): cultures without WiBi-induced neuralization with mesencephalic identity (Bertacchi et al., 2015). Mean ± SD is shown; ordinary one-way ANOVA with Tukey's multiple comparisons test was performed for *Foxg1* expression; unpaired t test was performed for *Pax6* and *Nkx2.1* markers.

(F) Relative expression of MGE-LGE markers by RT-qPCR ($n = 4$ independent experiments). Mean ± SD is shown; multiple unpaired t test with Holm-Šídák correction method. p values: *p value < 0.05, ***p value < 0.001, ****p value < 0.0001.

(G–L) Bottom: immunolabeling and quantification of CYC and SAG cultures for the indicated markers at the *in vitro* differentiation day (DIV) indicated in labels (blue nuclear counterstaining by DAPI). Scale bar, 30 μm. The *in situ* hybridization (ISH) insets at the figure top, showing the *in vivo* expression of the markers for comparison, are taken from Allen Brain Atlas: Developing Mouse Brain (https://developingmouse.brain-map.org/). In (L), mean ± SD and multiple unpaired t test with Holm-Šídák correction method are shown: **p value < 0.01, ***p value < 0.001, ****p value < 0.0001.

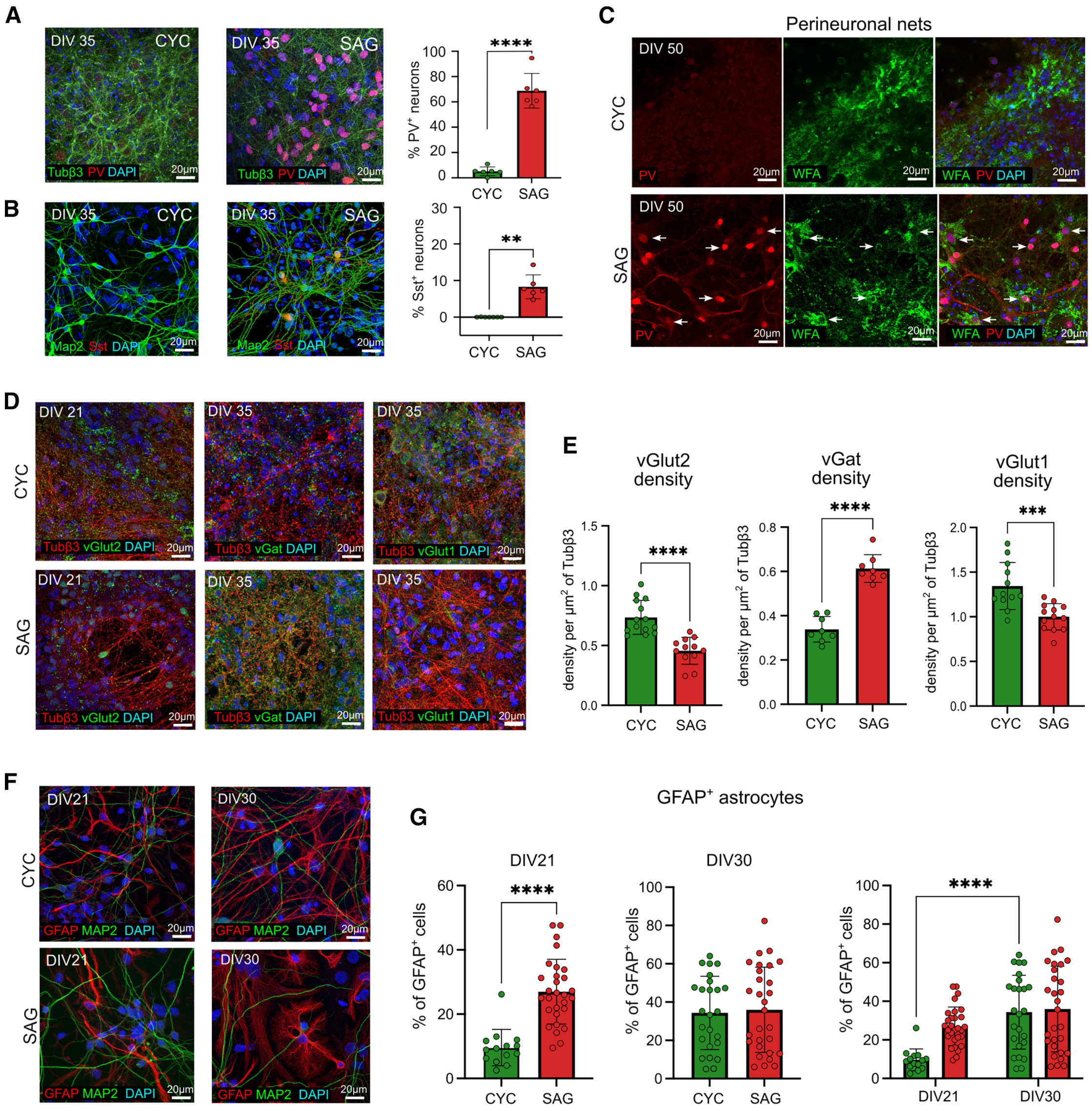

Figure 2. Expression of excitatory and inhibitory neuronal markers in CYC and SAG cultures

(A and B) Representative images and quantification of CYC and SAG cells stained with Tubβ3, Map2, parvalbumin (PV), somatostatin (Sst), and DAPI at DIV35. Scale bar, 20 μm. Mean ± SD and unpaired t test are shown for $n = 3$ independent experiments: $**p$ value < 0.01, $****p$ value < 0.0001.

(C) Representative images of CYC and SAG cells stained with the perineuronal net (PNN) markers PV and WFA at DIV50. White arrows indicate PNN-like structures surrounding PV^+ neurons. Scale bar, 20 μm.

(D) Representative confocal images of glutamatergic and GABAergic markers: vGlut2 (DIV21), vGat, and vGlut1 (DIV35). Scale bar, 20 μm.

(E) Quantification of glutamatergic and GABAergic markers shown in (D). Synaptic vesicles were measured in terms of density of Tubβ3 positive area covered by vesicles. Comparisons showed statistical differences between the two treatments ($n = 3$ independent experiments; unpaired t test).

(F and G) GFAP expression at DIV21 and DIV30 (Scale bar, 20 μm). In (G), mean ± SD and unpaired t test are shown at distinct DIV; two-way ANOVA followed by Šídák's multiple comparison test was performed for comparison over time. p values: $*p$ value < 0.05, $**p$ value < 0.01, $***p$ value < 0.001, $****p$ value < 0.0001.

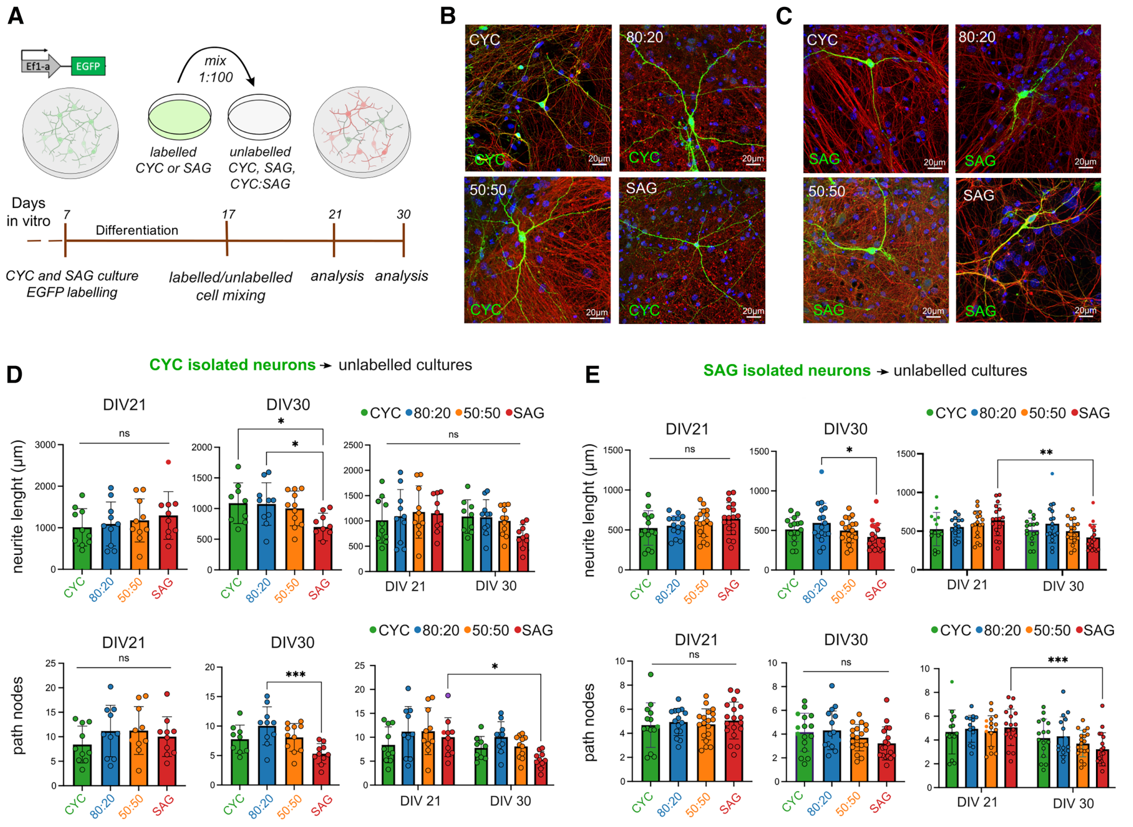

Figure 3. Morphological analysis of CYC and SAG neurons in pure and mixed cultures
(A) Method outline.
(B and C) Images of EGFP⁺-CYC or EGFP⁺-SAG neurons, respectively, in pure and mixed unlabeled cultures. Scale bar, 20 μm.
(D and E) Quantitative analysis of neurite length and path nodes at DIV21 and DIV30. Mean ± SD and ordinary one-way ANOVA with Tukey's multiple comparisons test are shown to compare samples at each time point; two-way ANOVA followed by Šídák's multiple comparisons test was performed for comparison over time. *p* values: **p* value < 0.05, ***p* value < 0.01, ****p* value < 0.001, *****p* value < 0.0001; ns, not significant.

Morphological analysis of CYC and SAG neurons in mixed cultures

The correct integration of VT neurons into the cortex is a regulated process requiring saltatory migration (Marín et al., 2010). We evaluated whether early SAG neurons can differentiate when mixed to isochronic CYC neurons and vice versa, in an *in vitro* environment and in the absence of regulated migration. To assess the maturation and development processes of CYC and SAG neurons, we firstly created four culture conditions: pure CYC, pure SAG, and mixed 80:20 and 50:50 (CYC:SAG) ratios. Hence, to enable the analysis of single-neuron morphology, we used EGFP-labeled CYC or SAG cells at DIV7 and mixed them at a 1:100 ratio into the four unlabeled cultures (Figures 3A–3C, S3A, and S3B). We observed CYC and SAG neurons at DIV21 (early development) and DIV30 (maturation onset), measuring neurite length, branching (nodes), and dendritic spine percentage. In the analysis, we avoided EGFP-labeled cells showing glial morphology (Figure S3E), which appeared in proportions similar to those detected through immunodetection.

At DIV30, we found that CYC neurons in the SAG environment showed decreased neurite length and fewer nodes compared to neurons in other environments, especially in 80:20 cultures, resulting in lower maturation (Figure 3D). Similarly, SAG neurons showed a significant decrease in the length of neurites in SAG cultures at DIV30 and over time, compared with those in 80:20 cultures. In addition, when comparing the two time points, we also observed a reduced number of nodes concluding that SAG neurons showed altered maturation in branching patterns (Figure 3E).

The analysis of the dendritic spines (Figures S3A and S3B) highlighted a reduced percentage of spines on CYC neurons in both 50:50 and SAG conditions at DIV21 and DIV30 (Figure S3C). In contrast, the

maturation of SAG neurons' spines seemed impaired at DIV21 in inhibitory-rich environments but showed no difference at DIV30 (Figure S3D). However, their spine percentage increased over time only in the 50:50 and SAG conditions, which is in line with the knowledge that medium spiny neurons in the striatum form abundant dendritic spines during development (Steiner and Tseng, 2017). Overall, our results indicate that the composition of the surrounding neuronal population might influence the morphological maturation of a neuron. We observed alterations in neurite length, branching complexity, and dendritic spine density in both CYC and SAG neurons when cultured in mostly inhibitory environments, suggesting that the diverse cellular environments may impact the functional integration and connectivity of these neurons within cortical circuits.

Functional activity development of pure and mixed CYC and SAG neuronal networks

Primary cortical neurons in culture spontaneously develop neuronal networks with various activity patterns (Charlesworth et al., 2015; Dias et al., 2021). The E/I balance forms the basis for functional neural networks, supporting cognition and memory. This balance is maintained at a single-neuron level by an appropriate ratio of excitatory to inhibitory synaptic inputs and at the network level by regulating the interaction between various excitatory and inhibitory circuits (He and Cline, 2019). To simulate various E/I conditions, we analyzed pure CYC cultures (lacking ventrally migrated interneurons), pure SAG cultures (enriched with GABAergic neurons), and mixed CYC:SAG cultures with different E/I ratios. Our analysis focused on the development of network activity patterns, specifically targeting the most abundant inhibitory neuron types, PV^+ and SST^+ neurons, which were maintained at consistent proportions throughout the maturation period. The immunostaining analysis showed that the relative proportions of PV^+ (Figures S4A and S4B) and SST^+ (Figures S4C and S4D) neurons remained the same at DIV30 and DIV45 (Figures S4E and S4F).

Using a high-density microelectrode array (HD-MEA) comprising 4,096 channels, we conducted a longitudinal study of pure CYC and SAG cultures and mixed cultures with an 80:20 or 50:50 ratio (Figure 4A). As CYC culture contained 7% of PV^+ cells and a negligible ratio of SST^+ cells (Figure 2A) and SAG culture contained 73% of PV^+ cells and 7% of SST^+ cells (Figure 2B), the theoretical number of PV^+ and SST^+ inhibitory neurons in 80:20 and 50:50 cultures is 21.6% and 43.5%, respectively. It should be noted, however, that the relative proportions of PV^+ and SST^+ neurons in both pure and mixed cultures differ from those observed in the cortex, where the ratio of inhibitory interneurons PV:Sst:VIP is approximately 40:30:30 (Druga et al., 2023), thus representing an artificial experimental condition.

We first analyzed the mean firing rate (MFR) per channel, the mean burst rate (MBR) and duration (MBD), and the percentage of bursting electrodes and of random spikes. MFR increased over the time in all cultures with no significant differences between them (Figure 4B; Videos S1, S2, S3, S4, and S5). However, a clear difference was observed in bursting activity. Mixed cultures had a much higher number of bursting electrodes (Figures 4C and 4D, Supplementary movies VS1,3,4), with bursts appearing first in 50:50 and 80:20 cultures (DIV33), followed by SAG cultures (DIV39) and finally CYC cultures (DIV45) (Figures 4E and 4F). MBR and MBD were also higher in mixed cultures compared to pure cultures (Figures 4E and 4F), indicating that combining the two cell types greatly enhances the ability to generate burst activity. Pure cultures showed the highest percentage of random spikes (Figure 4G), despite having a comparable MFR to mixed cultures (Figure 4B).

The pure CYC network developed different adhesion properties than SAG cultures (Figures S4G–S4I). They tended to form clusters (Figure S4G) with a thicker cell layer than SAG cultures (Figure S4I), although the average cell density was comparable between the two types of cultures (Figure S4H), suggesting different adhesion and connectivity properties. This led to a lower number of active channels in CYC cultures compared to mixed and SAG cultures (Figure S4J). However, the analysis of double-density cultures confirmed that the difference in activity between CYC and 80:20 networks was not due to cell proportion on the electrodes (Figure S4K).

Because SAG cultures are predominantly inhibitory (80% inhibitory neurons) and CYC cultures have only 7% inhibitory neurons, it appears that inhibitory neurons play a key role in establishing bursting activities. To test this, we simulated self-organizing artificial neural networks with varying neuronal E/I ratios using the Izhikevich spiking neuron model (2006). The model is simpler and faster than more complex biophysical models, making it ideal for studying large-scale networks (Izhikevich and Edelman, 2008). Our simulations of a 1,000-neuron network with varying E/I ratios showed that the Instantaneous Network Firing (INF, see methods), a metric that in Izhikevich's formulation most closely resembles the burst rate observed in biological networks, followed a trend similar to our biological networks: maximum INF occurred at E/I ratios between 800:200 and 500:500 (Figure S4L). Although the model aims to replicate the excitability and connectivity characteristics of cortical excitatory and inhibitory neurons,

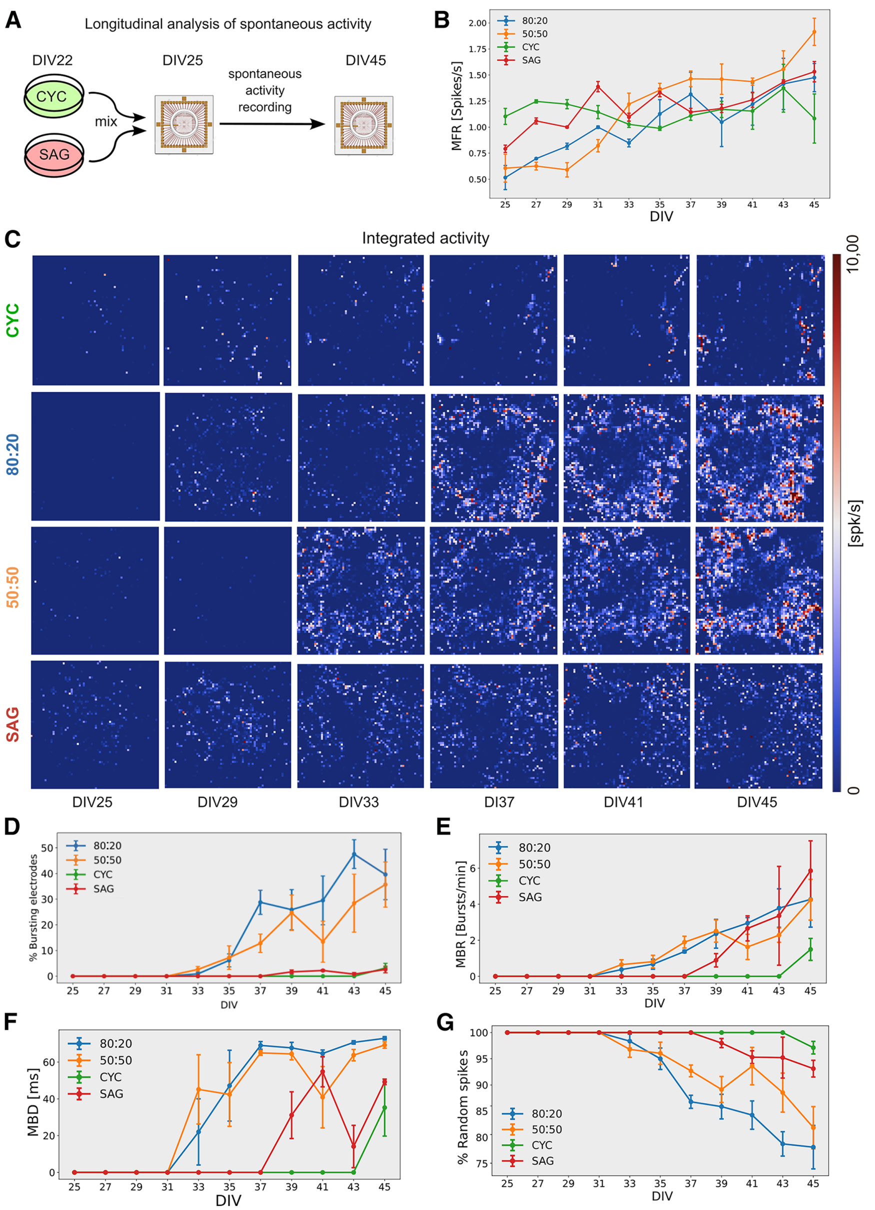

Figure 4. Longitudinal analysis of electrophysiological activity of CYC, SAG, and mixed cultures

(A) Experimental outline.

(B) Mean firing rate (MFR) of individual channels over time (*n* = 3 independent experiments; mean ± SEM is shown).

(C) Firing rate (spk/s) heatmap of representative HD-MEAs; each pixel represents the integrated activity (5 min) of one channel at different DIV.

(D–G) Percentage of bursting electrodes, mean burst rate (MBR), mean burst duration (MBD), and percentage of random spikes of individual channels at distinct DIV (*n* = 3 independent experiments; mean ± SEM is shown).

it remains a simplification. Nonetheless, the consistent results across two fundamentally different network types suggest that the E/I ratio's effect on burst activity is an emergent property arising from complex network interactions rather than being solely determined by individual neuronal characteristics.

Pure and mixed CYC and SAG cultures show distinct patterns of network burst activity

Spontaneous network activity underpins the development of functional networks in early stages (Teppola et al., 2019), and a key feature of this activity is the recurrence of intense network bursts (NBs) that rapidly propagate throughout the culture (Weihberger et al., 2013). We analyzed NBs in our cultures and found that they first appeared in mixed CYC:SAG cultures starting from DIV31 and then in pure SAG cultures, although at a very low extent, from DIV35, whereas they were almost absent in pure CYC cultures (Figures 5A and 5B).

At DIV45, NB duration (NBD) was comparable in the four cultures (Figure 5C), indicating that this parameter is independent of the NB frequency. The contrasting NB trends between CYC and 80:20 cultures, despite both having low PV^+ and high glutamatergic neuron percentages, suggest a difference in their excitatory/inhibitory connectivity. We thus investigated the role of

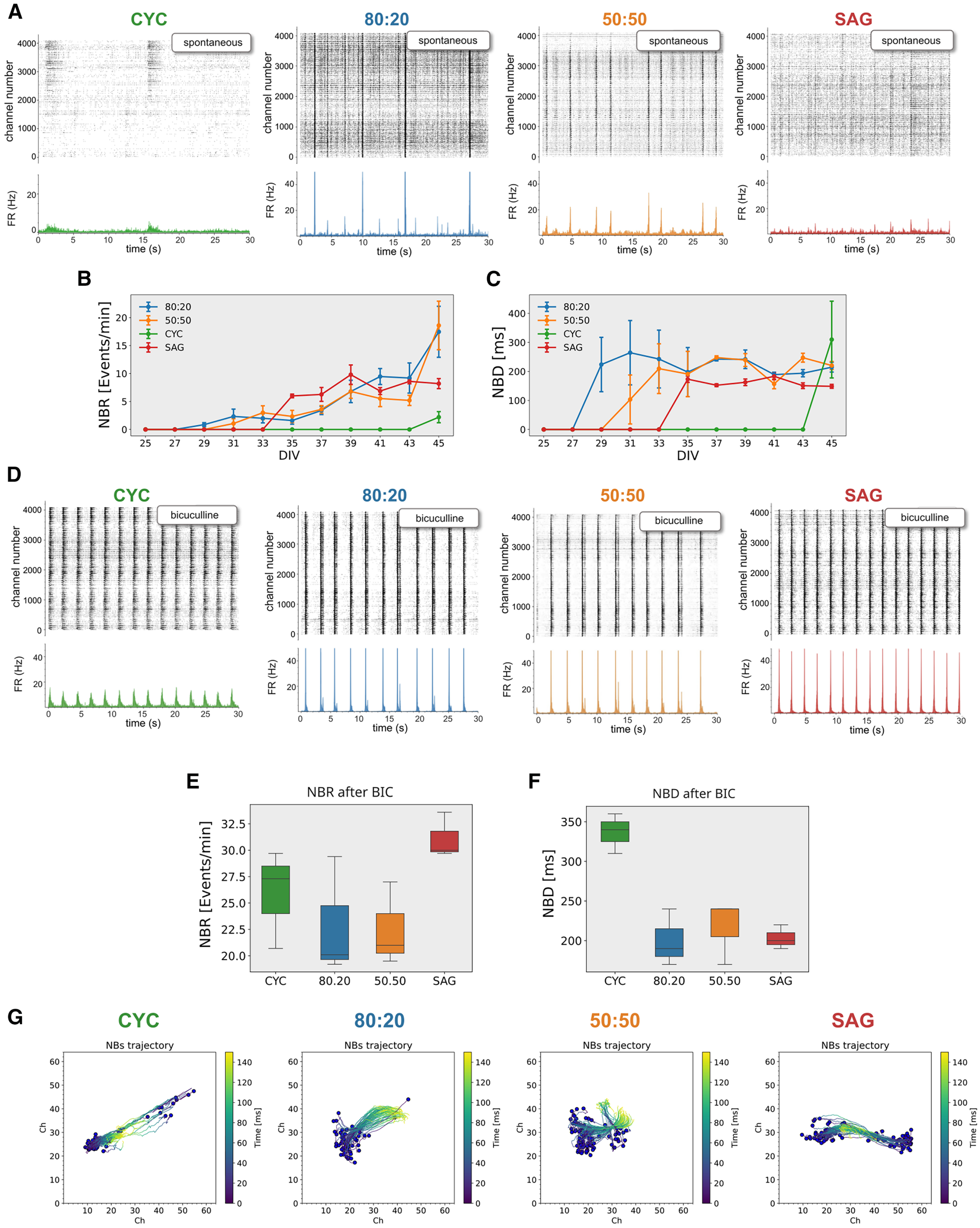

Figure 5. GABA inhibition critically affects network activity in pure and mixed CYC and SAG cultures

(A) Top: activity raster plot (spike times per channel) of representative HD-MEA at the analysis endpoint (DIV45); bottom: average network firing rate.

(B and C) Network burst rate (NBR) and network burst duration (NBD) (*n* = 3 independent experiments; mean ± SEM is shown).

(D) Analysis as in (A), after BIC administration.

(E and F) NBR and NBD after BIC administration (*n* = 3 independent experiments).

(G) Analysis of the center of activity trajectories (CATs). Each blue dot represents the physical center of mass of the activity where the NB starts, while the colored line represents its own trajectory. The colored scale bar represents the time elapsed during the propagation of an NB.

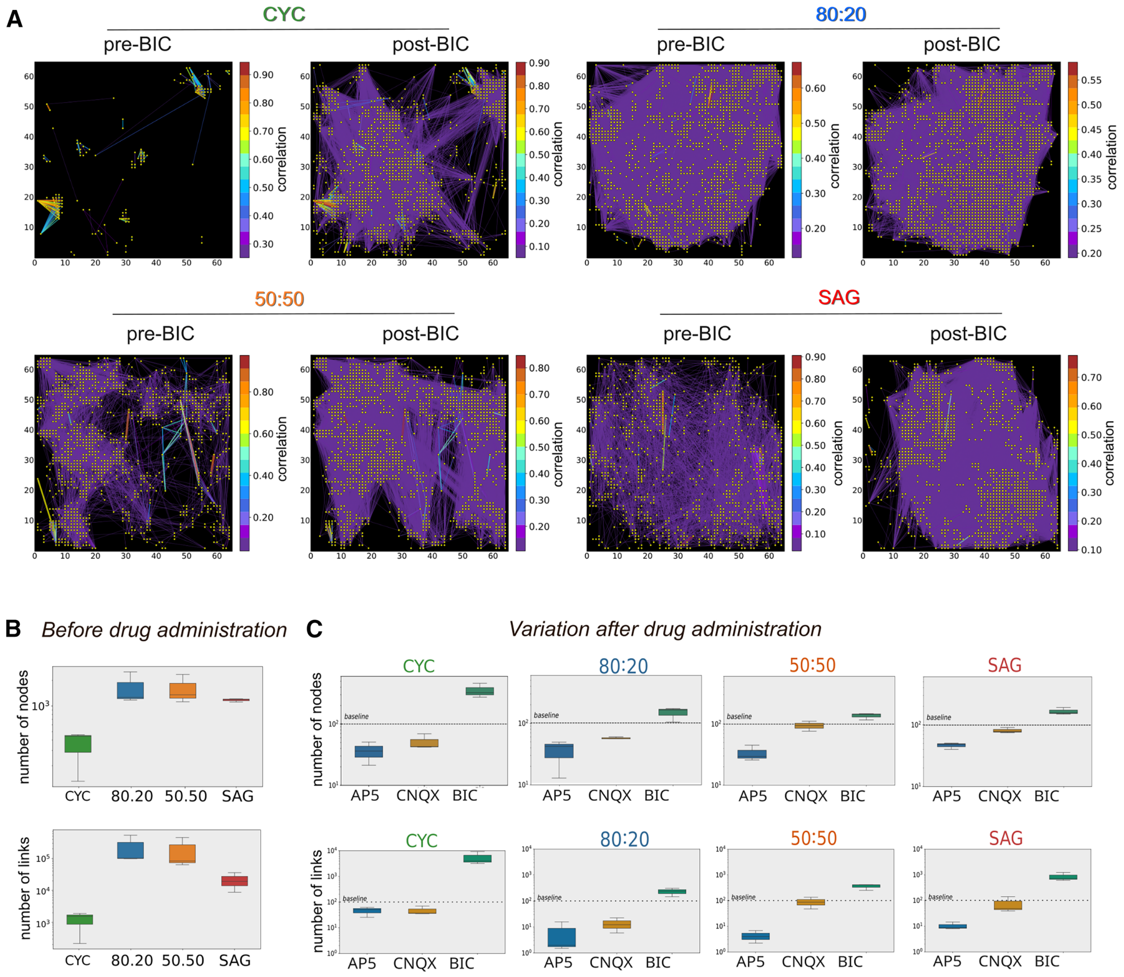

Figure 6. GABA inhibition unmasks complex connectivity of the CYC network

(A) Connectivity plots of representative neuronal cultures during spontaneous activity before (pre-BIC) and after (post-BIC) BIC administration. Each yellow point represents a node of the functional graph; colored lines represent the correlation strength between two points (only the 10% of the functional links are shown). The color bar indicates the correlation index.

(B and C) Number of nodes and links before and after drug administration, respectively (*n* = 3 independent experiments).

GABA, AMPA, and NMDA receptors in NB occurrence of mature networks (DIV45). Antagonists for AMPA (CNQX) and NMDA (AP5) receptors blocked NBs in all cultures (Figures S5A–S5E), consistent with their known role in burst activity (Jimbo et al., 2000). As the four cultures have different degrees of GABAergic signaling, mainly due to the presence of different percentages of PV^{+} and SST^{+} interneurons, they respond differently to the administration of bicuculline (BIC), a $GABA_A$ receptor antagonist. Mixed 80:20 and 50:50 cultures showed a slight increase in NB rate after BIC administration, while pure CYC and SAG cultures showed a massive increase in both rate and duration (Figures 5D–5F). Notably, CYC NBR was even higher than the 80:20 one, suggesting that this effect involves the activity recruitment of new neurons and is independent of cell density (see Figure S5F). This observation in the CYC culture, despite its low number of inhibitory neurons, indicates that the ratio of PV^{+} and SST^{+} cells does not directly correlate with NB activity. We believe that this may be due to the development of different structural and functional connectivity patterns, although this hypothesis requires further experimental investigation.

Using center of activity trajectory analysis (Chao et al., 2007) to quantify network burst events, we found that when inhibitory activity was suppressed by BIC, NBs propagated with similar properties across all four cultures (Figure 5G). In conclusion, BIC appeared to unmask an intrinsic capacity of CYC and SAG cultures to generate and propagate NBs.

GABAergic and glutamatergic components differently impact on pure and mixed CYC and SAG functional networks

As GABAergic inhibition is responsible for masking the intrinsic NB activity of pure cultures compared to mixed cultures, we investigated whether a specific functional connectivity analysis could explain the different effect of BIC on the NB activity of the four conditions. We compared functional network connectivity, which is the activity correlation between channels, before and after administering BIC (Figure 6A), CNQX, and AP5 (Figure S6). The correlation analysis allowed us to calculate the number of nodes and links of the spontaneous network activity (Figure 6B) and their changes after drug administration (Figure 6C). In the absence of drugs, the mixed cultures showed a similar number of nodes and links while SAG and CYC networks had fewer, with CYC showing the lowest number (Figure 6B). Each drug affected the four cultures differently (Figures 6C and S6). BIC had its most pronounced effect on CYC cultures, where it dramatically increased the number of nodes and links. This is significant because SAG cultures have a much higher proportion of PV^+ and SST^+ neurons than CYC cultures. Our results suggest that a very complex part of the CYC functional network was under strong GABAergic inhibition. This is consistent with studies showing that PV^+ interneurons can significantly influence the excitatory activity of pyramidal neurons to compensate for overactive excitatory forces (Haider et al., 2006; Xue et al., 2014). Finally, while CNQX had almost no effect on SAG and 50:50 cultures as compared to CYC and 80:20, AP5 decreased the number of nodes and links in all the cultures, although at different extents (Figure 6C). This indicates that the activity of NMDA receptors is important in the developmental regulation of synaptic transmission, also mediated by both AMPA and $GABA_A$ receptors (Lu et al., 2011; Marsden et al., 2007), and plays a pivotal role in establishing NB activity and maintaining the delicate E/I balance.

Network stimulation discloses different signal propagation properties of pure and mixed cultures

We analyzed the patterns of activity evoked by electrical stimulation in our cultures and compared them to the patterns of spontaneous activity. At DIV50, we stimulated a single channel with 25 biphasic pulses. We measured the response by comparing spike counts before and after each pulse and averaging the responses over 25 repetitions. The single-channel stimulation evoked the activity of channels located at different distances within a 5 ms delay, suggesting direct functional connectivity (Figures 7A and S7A–S7D). The 80:20 condition was the most responsive, with the stimulus first recruiting a high number of positively correlated channels (red channels in Figure S7B), followed by the appearance of negatively correlated channels (yellow channels in Figure S7B). In 50:50 and SAG cultures, the induction of positively correlated active channels was moderate, while the appearance of negatively correlated channels was more pronounced and earlier compared to 80:20 cultures (Figures S7C and S7D). CYC cultures behaved differently, showing recruitment of almost all activated channels within 5 ms and appearance of negatively correlated channels with a 60 ms delay, more like the 80:20 culture (Figure S7A).

We also analyzed the spatial dispersion of the signal based on the physical distance from the stimulation point (dispersion index in Figure 7B). Our findings reveal a correlation between the dispersion index of the signal evoked by the stimulus and the PV^+ cell ratio. All four cultures had a common peak in the dispersion index around 0.3–0.4 mm at 150 ms post stimulation. The 80:20 cultures had the most robust overall response but a low dispersion index at early times, suggesting a tendency to form local circuits. Conversely, 50:50 and SAG cultures showed more distant initial responses but a significantly lower overall response. We hypothesized that stronger inhibition in these cultures may locally suppress the initial response, promoting it farther from the stimulation site. We concluded that intermediate ratios of inhibitory neurons provide the best global activity due to a balance between signal propagation and inhibition.

Single-channel stimulation was never able to propagate to the entire network and to generate NBs. We speculated that stimulating a sufficient number of spontaneously active electrodes could evoke NBs. We selected the 7 most active channels in each culture for simultaneous stimulation. In 80:20, 50:50, and SAG conditions, the electrical stimulation evoked a synchronized network activity comparable to spontaneous NBs (Figures 7C–7E). CYC cultures did not generate NBs in response to the stimulus (Figure 7F): indeed, the response did not propagate beyond the activated channels (enlarged detail of the raster plot in Figure 7F). This last observation suggests that a proper inhibitory ratio is required to integrate and organize a network circuit capable of propagating NBs (Tremblay et al., 2016). Moreover, we evaluated the duration of the induced NB (NBD). In the 80:20 configuration (Figure 7C), the duration was similar to the one of spontaneous NBs, leaving the network almost unchanged. In 50:50 and SAG conditions, the duration of the induced NBs was shorter than the spontaneous NBs (Figures 7D and 7E) and chemically induced NBs (Figure 5F). These findings demonstrate that the presence of PV^+ interneurons significantly impacts the dispersion of neuronal activity, influencing network connectivity and determining the capacity of cortical networks to generate synchronous NBs.

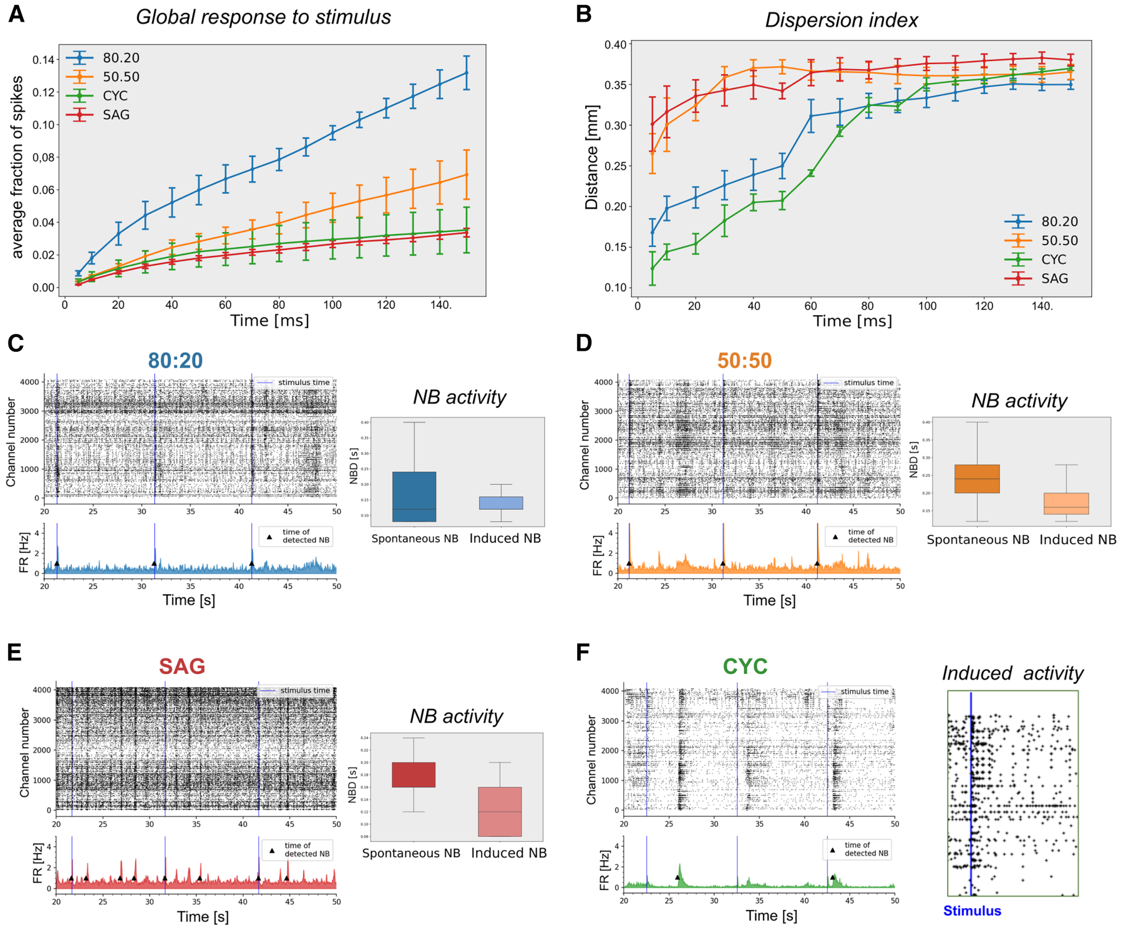

Figure 7. Global response, dispersion index, and NBs evoked by stimulus are affected by E/I ratio
(A) Distribution of the global response to single-channel stimulation, showing the average fraction of spikes starting from 5 to 150 ms (n = 3 independent experiments; mean ± SEM is shown).
(B) Dispersion index indicating the distance of responses from the stimulated channel at different times from the stimulus (n = 3 independent experiments; mean ± SEM is shown).
(C–F) Representative raster plots (C–F) and boxplots (C–E) showing NBs and NBD, respectively, during multiple electrode stimulation. The stimulus time is indicated by the blue line. On the right in (F): enlarged detail of CYC raster plot, highlighting only the electrodes that show a response to the stimulus (n = 3 independent experiments).

DISCUSSION

We studied how isolated and mixed cultures of DT and VT neurons develop and mature *in vitro*. Our analysis showed that these cultures, which have different molecular identities, also develop distinct network activity patterns.

CYC and SAG cultures expressed key marker genes consistent with DT and VT identity and matured opposite ratios of PV$^+$ and SST$^+$ inhibitory interneurons, very high (73% and 7%) in SAG cultures and very low (7% and less than 1%) in CYC cultures. Moreover, CYC cultures were enriched in glutamatergic vGlut1$^+$ neurons (73%) whereas vGlut1$^+$ neurons were 16% in SAG cultures. We observed that isolated CYC and SAG neurons could mature in each other's cultures, allowing us to investigate the activity properties of networks with different E/I balance. We aimed to understand the basic requirements for generating neuronal networks that can produce spontaneous, correlated activity, similar to what's seen in early brain regions (Chiu and Weliky, 2001; Crochet et al., 2005). Although pure CYC cultures and mixed cultures had a similar MFR, the pure CYC cultures showed very little correlated (burst) activity. This changed when a low percentage of SAG neurons were added: the 80:20 cultures exhibited more synchronous firing patterns. This also reduced the percentage of random spikes and increased the number of bursting electrodes, significantly boosting the network burst rate (NBR) and NBD. This is a puzzling finding, because SAG

cultures, which contain 80% PV^+ and SST^+ GABAergic neurons, show almost no network bursting activity on their own.

Sukenik et al. sorted hippocampal neurons from E17 mouse embryos by fluorescence-activated cell sorting into GAD^+ and GAD^- populations, which were then seeded at different ratios onto glial layers in microfluidic chambers (Sukenik et al., 2021). Using patch-clamp analysis, the authors observed that an increase in the percentage of inhibitory neurons in primary neuronal cultures leads to a reduction of the total number of active incoming connections received by a neuron. This mechanism allows the network to maintain a similar E/I balance and, consequently, to stabilize its spontaneous excitatory activity, adapting to diverse cellular compositions. While this observation is in line with the similar values of channel MFR that we observed in our different cultures (Figure 4B), it does not explain the lack of proportionality between E/I and synchronous network activity. Thus, we speculated that a proper ratio of GABAergic neurons is required to mature local circuitry capable of burst activity formation and spreading. Our findings indicate that bursting activity peaks within an intermediate range of E/I ratios, both *in vitro* and *in silico* networks. The remarkable consistency across these disparate network models—namely *in vivo* biological systems and *in silico* computational simulations—suggests that the observed relationship between E/I ratio and bursting activity is not merely a sum of individual neuronal characteristics. Instead, it appears to be an emergent property, arising from the intricate and complex interactions within the neural network itself. This emphasizes the critical role of network dynamics in shaping neuronal activity patterns, rather than solely attributing them to intrinsic cellular properties. However, the mechanistic explanation of this phenomenon remains to be addressed both through *in silico* network models and in biological networks.

The presence of different circuits in pure and mixed cultures is suggested by the different types of network activity generated under spontaneous conditions, upon GABAergic release by BIC administration or electrical stimulation. CYC cultures, which showed the lowest spontaneous NBR, generated the highest NBR together with SAG cultures after BIC treatment but were unable to induce NBs upon electrical stimulation. These observations, together with the shortest dispersion index shown by CYC cultures, point out that a too high E/I ratio develops a different network circuitry, confirming that an optimal E/I ratio is required to form local inhibitory networks capable of developing and spreading correlated activity.

Few studies have so far modeled cortical networks with precise E/I ratios. Different E/I ratios of iNeurons were used to evaluate the seizure liability of compounds by microelectrode array (MEA), identifying the 84/16 E/I balance as the best suited to detect concentration-dependent changes and to classify the mechanism of action of seizurogenic compounds (Yokoi et al., 2022). Interestingly, this intermediate E/I balance showed the highest NB activity compared to higher and lower balances, in accordance with our observations that NBs are better supported by an intermediate E/I balance. Yokoi and co-workers observed that iNeuron cultures with the lowest E/I balance have the highest activity and the lowest response to GABA inhibitors, contrary to our observations in mESC cultures. A possible explanation for this difference could lie in the diverse characteristics and composition of the cell subtypes (i.e., different ratios of PV^+ SST^+ interneurons) and the connectivity of the circuits formed in the two types of culture. MEA recordings were also utilized by other groups to study hiPSC-derived networks with varying E/I balance (see introduction) (Parodi et al., 2023, 2024). These studies, which used different types of iNeurons, reported contrasting behaviors of networks with the highest E/I balance (100:0), showing lower or higher network activity and NBR than the other E/I balances in the case of low- or high-density MEAs, respectively. However, the different molecular nature of iNeurons compared to our cultures, especially the expression of non-telencephalic markers (Lin et al., 2021) and the lack of PV^+ interneurons (Zhang et al., 2013) (see introduction), makes it difficult to compare the different datasets.

In our study, we expanded the analysis of various networks by examining their capacity to propagate stimulus-induced activity, underscoring the pivotal role of E/I balance. In addition, our study analyzes the network connectivity at two levels, structural (dendritic branching and number of nodes, Figure 3) and functional (Figures 4, 5, 6, and 7). By taking into account these network parameters, which were neglected in previous research, we could better explain divergent activity patterns in networks with different E/I balances.

Our current *in vitro* cortical network model, derived from *in vivo* observations, has limitations compared to an intact *in vivo* network. These include differing ratios of PV^+ and SST^+ inhibitory interneurons, absence of VIP^+ neurons, lack of thalamic afferents, and the general absence of vascularization, microglia, and fine cellular complexity. Despite these differences, our findings suggest that our model offers significant advantages over iNeuron-based models, as it more faithfully recapitulates the cortical identity of glutamatergic neurons and PV^+ interneurons. When applied to hiPSs, it represents a powerful tool for studying the role of E/I balance in shaping network activity during human neurodevelopment, particularly in the context of neurodevelopmental disorders.

METHODS

mESCs were differentiated into DT or VT lineages following a four-step protocol. In the first step (DIV0–DIV5), cells were cultured in a chemically defined minimal medium supplemented with Wnt and BMP inhibitors (WiBi) and plated on poly-ornithine/laminin-coated (PL) surfaces. In the second step (DIV6–DIV10), DT and VT cells were generated adding cyclopamine (3 μM) and SAG (0.1 μM), respectively, to the WiBi medium. In the third step (DIV11-DIV20), cells were replated onto PL and maintained in neurobasal-A. From DIV20 onward, neurons were maintained in neurobasal-A medium supplemented with 0.2 mM ascorbic acid and 20 ng/mL recombinant human BDNF protein. To prepare the mixed cultures of 80:20 and 50:50, neurons were splitted at specific time points. For MEA preparation, CYC and SAG neurons were detached at DIV22, counted, mixed with the designed proportions and seeded on sterilized HD-MEA (Accura, 3Brain) (60,000 cells/chip), and allowed to adhere O/N.

Quantification analyses for density of glutamatergic and GABAergic vesicles were done by using the ImageJ Synapse Counter plugin (https://github.com/SynPuCo/SynapseCounter). To study neuronal development in pure and mixed cultures, CYC and SAG cells were transduced with EGFP lentivirus at DIV7 and selected via PuroR. At DIV17, EGFP-labeled and unlabeled neurons were combined to create pure (cyclopamine-only, SAG-only) and mixed (80:20, 50:50) cultures, each with 1% EGFP-positive cells, and seeded on PL-treated glass (150,000 cells/cm^2). Cultures were fixed with 2% paraformaldehyde at DIV21 and DIV30 and analyzed by immunofluorescence. Neural length and the node number were evaluated by the Fiji SNT plugin.

Electrophysiological recordings were conducted using high-density CMOS-based 4096 microelectrode arrays (Accura, 3Brain) to evaluate spiking activity, bursting behavior, and network synchronization. Drug responses and functional connectivity were analyzed using custom Python scripts, with spike sorting and network analysis performed using principal-component analysis and cross-correlation methods.

The response to stimuli was measured by comparing spike counts before and after the stimulus within a defined time window (5–150 ms). The stimulus was repeated 25 times, responses were averaged, and statistical confidence intervals were calculated. For data visualization, responses were displayed on a grid, with pixel intensity indicating response strength. The dispersion index, quantifying how spread out significant responses were across the grid, was calculated by estimating the average distances between responsive electrodes.

The self-organizing artificial neural network modeling was performed as described by Izhikevich, 2006 (see supplemental methods).

Comprehensive descriptions of all experimental procedures are provided in the methods section of the supplemental information.

RESOURCE AVAILABILITY

Lead contact

Further information and requests for resources and reagents should be directed to and will be fulfilled by the lead contact, Federico Cremisi (federico.cremisi@sns.it).

Materials availability

This study did not generate new unique reagents. However, any questions about reagents or animals used can be directed to the lead contact.

Data and code availability

This study did not generate new sequencing data. Any questions about gene expression experiments can be directed to the lead contact.

ACKNOWLEDGMENTS

We are thankful to Robert Vignali and Lucio Calcagnile for helpful discussions, Maria Antonietta Calvello, Vania Liverani, and Alessandro Puntoni for technical support, Michèle Studer and Michele Bertacchi for advise in marker immunodetection analysis, and Stefano Guglielmo, Claudia Alia, and Nicola Origlia for advice on the functional analysis of cultured networks. The research was supported by the Matteo Caleo Foundation, by intramural funding of IIT (L.P.) and Scuola Normale Superiore (F.C.), by the PRIN grant #2022M95RC7 from the Italian Ministry of University and Research (F.C.), and by the Tuscany Health Ecosystem – THE grant from MUR (F.C. and A.D.G.). This work was funded by the European Union – Next Generation EU, Mission 4 Component 1 CUP E53C24001460006, project TNE – NEUROBRIDGE, which covered the publication costs.

AUTHOR CONTRIBUTIONS

E.C. and F.C. conceptualized and designed the study and wrote the manuscript. E.C., F.T., and L.I. performed the experiments. E.C. set up the strategy of culture treatments, the connectivity analysis, and the longitudinal functional study. F.T. set up MEA stimulation. L.P. conceptualized the experiments of gene expression analysis. L.I. set up the time-series analysis of MEA data under the advice of A.D.G. G.L. performed the analysis of MEA stimulation under the supervision of G.A. M.F. modeled the artificial neural networks with STDP. All authors discussed the results and commented on the manuscript.

DECLARATION OF INTERESTS

The authors declare no competing interests.

SUPPLEMENTAL INFORMATION

Supplemental information can be found online at https://doi.org/10.1016/j.stemcr.2025.102646.

Received: February 5, 2025
Revised: August 26, 2025
Accepted: August 27, 2025
Published: September 25, 2025

REFERENCES

Bertacchi, M., Pandolfini, L., D'Onofrio, M., Brandi, R., and Cremisi, F. (2015). The double inhibition of endogenously produced BMP and W nt factors synergistically triggers dorsal telencephalic differentiation of mouse ES cells. Dev. Neurobiol. *75*, 66–79. https://doi.org/10.1002/dneu.22209.

Cao, S.-Y., Hu, Y., Chen, C., Yuan, F., Xu, M., Li, Q., Fang, K.-H., Chen, Y., and Liu, Y. (2017). Enhanced derivation of human pluripotent stem cell-derived cortical glutamatergic neurons by a small molecule. Sci. Rep. *7*, 3282. https://doi.org/10.1038/s41598-017-03519-w.

Cederquist, G.Y., Asciolla, J.J., Tchieu, J., Walsh, R.M., Cornacchia, D., Resh, M.D., and Studer, L. (2019). Specification of positional identity in forebrain organoids. Nat. Biotechnol. *37*, 436–444. https://doi.org/10.1038/s41587-019-0085-3.

Chambers, S.M., Fasano, C.A., Papapetrou, E.P., Tomishima, M., Sadelain, M., and Studer, L. (2009). Highly efficient neural conversion of human ES and iPS cells by dual inhibition of SMAD signaling. Nat. Biotechnol. *27*, 275–280. https://doi.org/10.1038/nbt.1529.

Chao, Z.C., Bakkum, D.J., and Potter, S.M. (2007). Region-specific network plasticity in simulated and living cortical networks: comparison of the center of activity trajectory (CAT) with other statistics. J. Neural. Eng. *4*, 294–308. https://doi.org/10.1088/1741-2560/4/3/015.

Charlesworth, P., Cotterill, E., Morton, A., Grant, S.G.N., and Eglen, S.J. (2015). Quantitative differences in developmental profiles of spontaneous activity in cortical and hippocampal cultures. Neural Dev. *10*, 1. https://doi.org/10.1186/s13064-014-0028-0.

Chen, J.K., Taipale, J., Cooper, M.K., and Beachy, P.A. (2002a). Inhibition of Hedgehog signaling by direct binding of cyclopamine to Smoothened. Genes Dev. *16*, 2743–2748. https://doi.org/10.1101/gad.1025302.

Chen, J.K., Taipale, J., Young, K.E., Maiti, T., and Beachy, P.A. (2002b). Small molecule modulation of Smoothened activity. Proc. Natl. Acad. Sci. USA *99*, 14071–14076. https://doi.org/10.1073/pnas.182542899.

Chen, Y.-J.J., Friedman, B.A., Ha, C., Durinck, S., Liu, J., Rubenstein, J.L., Seshagiri, S., and Modrusan, Z. (2017). Single-cell RNA sequencing identifies distinct mouse medial ganglionic eminence cell types. Sci. Rep. *7*, 45656. https://doi.org/10.1038/srep45656.

Chiaradia, I., and Lancaster, M.A. (2020). Brain organoids for the study of human neurobiology at the interface of *in vitro* and *in vivo*. Nat. Neurosci. *23*, 1496–1508. https://doi.org/10.1038/s41593-020-00730-3.

Chiu, C., and Weliky, M. (2001). Spontaneous Activity in Developing Ferret Visual Cortex *In Vivo*. J. Neurosci. *21*, 8906–8914. https://doi.org/10.1523/JNEUROSCI.21-22-08906.2001.

Crochet, S., Chauvette, S., Boucetta, S., and Timofeev, I. (2005). Modulation of synaptic transmission in neocortex by network activities. Eur. J. Neurosci. *21*, 1030–1044. https://doi.org/10.1111/j.1460-9568.2005.03932.x.

Dias, I., Levers, M.R., Lamberti, M., Hassink, G.C., Van Wezel, R., and Le Feber, J. (2021). Consolidation of memory traces in cultured cortical networks requires low cholinergic tone, synchronized activity and high network excitability. J. Neural. Eng. *18*, 046051. https://doi.org/10.1088/1741-2552/abfb3f.

Dickens, S., Goodenough, A., and Kwok, J. (2022). An *in vitro* neuronal model replicating the *in vivo* maturation and heterogeneity of perineuronal nets. Preprint at bioRxiv. https://doi.org/10.1101/2022.01.22.477344.

Fuccillo, M., Joyner, A.L., and Fishell, G. (2006). Morphogen to mitogen: the multiple roles of hedgehog signalling in vertebrate neural development. Nat. Rev. Neurosci. *7*, 772–783. https://doi.org/10.1038/nrn1990.

Gaspard, N., Bouschet, T., Hourez, R., Dimidschstein, J., Naeije, G., Van Den Ameele, J., Espuny-Camacho, I., Herpoel, A., Passante, L., Schiffmann, S.N., et al. (2008). An intrinsic mechanism of corticogenesis from embryonic stem cells. Nature *455*, 351–357. https://doi.org/10.1038/nature07287.

Gelman, D.M., and Marín, O. (2010). Generation of interneuron diversity in the mouse cerebral cortex. Eur. J. Neurosci. *31*, 2136–2141. https://doi.org/10.1111/j.1460-9568.2010.07267.x.

Gelman, D., Griveau, A., Dehorter, N., Teissier, A., Varela, C., Pla, R., Pierani, A., and Marín, O. (2011). A Wide Diversity of Cortical GABAergic Interneurons Derives from the Embryonic Preoptic Area. J. Neurosci. *31*, 16570–16580. https://doi.org/10.1523/JNEUROSCI.4068-11.2011.

Haider, B., Duque, A., Hasenstaub, A.R., and McCormick, D.A. (2006). Neocortical Network Activity In Vivo Is Generated through a Dynamic Balance of Excitation and Inhibition. J. Neurosci. *26*, 4535–4545. https://doi.org/10.1523/JNEUROSCI.5297-05.2006.

He, H.Y., and Cline, H.T. (2019). What Is Excitation/Inhibition and How Is It Regulated? A Case of the Elephant and the Wisemen. J. Exp. Neurosci. *13*, 1179069519859371. https://doi.org/10.1177/1179069519859371.

Izhikevich, E.M., and Edelman, G.M. (2008). Large-scale model of mammalian thalamocortical systems. Proc. Natl. Acad. Sci. USA *105*, 3593–3598. https://doi.org/10.1073/pnas.0712231105.

Jimbo, Y., Kawana, A., Parodi, P., and Torre, V. (2000). The dynamics of a neuronal culture of dissociated cortical neurons of neonatal rats. Biol. Cybern. *83*, 1–20. https://doi.org/10.1007/PL00007970.

Kempf, J., Knelles, K., Hersbach, B.A., Petrik, D., Riedemann, T., Bednarova, V., Janjic, A., Simon-Ebert, T., Enard, W., Smialowski, P., et al. (2021). Heterogeneity of neurons reprogrammed from spinal cord astrocytes by the proneural factors Ascl1 and Neurogenin2. Cell Rep. *36*, 109409. https://doi.org/10.1016/j.celrep.2021.109409.

Li, X.-J., Zhang, X., Johnson, M.A., Wang, Z.-B., LaVaute, T., and Zhang, S.-C. (2009). Coordination of sonic hedgehog and Wnt signaling determines ventral and dorsal telencephalic neuron types from human embryonic stem cells. Development *136*, 4055–4063. https://doi.org/10.1242/dev.036624.

Lim, L., Mi, D., Llorca, A., and Marín, O. (2018). Development and Functional Diversification of Cortical Interneurons. Neuron *100*, 294–313. https://doi.org/10.1016/j.neuron.2018.10.009.

Lin, H.-C., He, Z., Ebert, S., Schörnig, M., Santel, M., Nikolova, M. T., Weigert, A., Hevers, W., Kasri, N.N., Taverna, E., et al. (2021). NGN2 induces diverse neuron types from human pluripotency. Stem Cell Rep. *16*, 2118–2127. https://doi.org/10.1016/j.stemcr.2021.07.006.

Lodato, S., Rouaux, C., Quast, K.B., Jantrachotechatchawan, C., Studer, M., Hensch, T.K., and Arlotta, P. (2011). Excitatory Projection Neuron Subtypes Control the Distribution of Local Inhibitory Interneurons in the Cerebral Cortex. Neuron *69*, 763–779. https://doi.org/10.1016/j.neuron.2011.01.015.

Lu, W., Gray, J.A., Granger, A.J., During, M.J., and Nicoll, R.A. (2011). Potentiation of Synaptic AMPA Receptors Induced by the Deletion of NMDA Receptors Requires the GluA2 Subunit. J. Neurophysiol. *105*, 923–928. https://doi.org/10.1152/jn.00725.2010.

Lupori, L., Totaro, V., Cornuti, S., Ciampi, L., Carrara, F., Grilli, E., Viglione, A., Tozzi, F., Putignano, E., Mazziotti, R., et al. (2023). A comprehensive atlas of perineuronal net distribution and colocalization with parvalbumin in the adult mouse brain. Cell Rep. *42*, 112788. https://doi.org/10.1016/j.celrep.2023.112788.

Marín, O. (2012). Interneuron dysfunction in psychiatric disorders. Nat. Rev. Neurosci. *13*, 107–120. https://doi.org/10.1038/nrn3155.

Marín, O., Valiente, M., Ge, X., and Tsai, L.-H. (2010). Guiding Neuronal Cell Migrations. Cold Spring Harb. Perspect. Biol. *2*, a001834. https://doi.org/10.1101/cshperspect.a001834.

Marsden, K.C., Beattie, J.B., Friedenthal, J., and Carroll, R.C. (2007). NMDA Receptor Activation Potentiates Inhibitory Transmission through GABA Receptor-Associated Protein-Dependent Exocytosis of GABAA Receptors. J. Neurosci. *27*, 14326–14337. https://doi.org/10.1523/JNEUROSCI.4433-07.2007.

Martynoga, B., Morrison, H., Price, D.J., and Mason, J.O. (2005). Foxg1 is required for specification of ventral telencephalon and region-specific regulation of dorsal telencephalic precursor proliferation and apoptosis. Dev. Biol. *283*, 113–127. https://doi.org/10.1016/j.ydbio.2005.04.005.

Mossink, B., Van Rhijn, J.-R., Wang, S., Linda, K., Vitale, M.R., Zöller, J.E.M., Van Hugte, E.J.H., Bak, J., Verboven, A.H.A., Selten, M., et al. (2022). Cadherin-13 is a critical regulator of GABAergic modulation in human stem-cell-derived neuronal networks. Mol. Psychiatry *27*, 1–18. https://doi.org/10.1038/s41380-021-01117-x.

Nelson, S.B., and Valakh, V. (2015). Excitatory/Inhibitory Balance and Circuit Homeostasis in Autism Spectrum Disorders. Neuron *87*, 684–698. https://doi.org/10.1016/j.neuron.2015.07.033.

Nery, S., Fishell, G., and Corbin, J.G. (2002). The caudal ganglionic eminence is a source of distinct cortical and subcortical cell populations. Nat. Neurosci. *5*, 1279–1287. https://doi.org/10.1038/nn971.

Parodi, G., Brofiga, M., Pastore, V.P., Chiappalone, M., and Martinoia, S. (2023). Deepening the role of excitation/inhibition balance in human iPSCs-derived neuronal networks coupled to MEAs during long-term development. J. Neural. Eng. *20*, 056011. https://doi.org/10.1088/1741-2552/acf78b.

Parodi, G., Zanini, G., Chiappalone, M., and Martinoia, S. (2024). Electrical and chemical modulation of homogeneous and heterogeneous human-iPSCs-derived neuronal networks on high density arrays. Front. Mol. Neurosci. *17*, 1304507. https://doi.org/10.3389/fnmol.2024.1304507.

Sohal, V.S., and Rubenstein, J.L.R. (2019). Excitation-inhibition balance as a framework for investigating mechanisms in neuropsychiatric disorders. Mol. Psychiatry *24*, 1248–1257. https://doi.org/10.1038/s41380-019-0426-0.

Steiner, H., and Tseng, K.-Y. (2017). Handbook of Basal Ganglia Structure and Function (Elsevier Academic Press).

Sukenik, N., Vinogradov, O., Weinreb, E., Segal, M., Levina, A., and Moses, E. (2021). Neuronal circuits overcome imbalance in excitation and inhibition by adjusting connection numbers. Proc. Natl. Acad. Sci. USA *118*, e2018459118. https://doi.org/10.1073/pnas.2018459118.

Teppola, H., Aćimović, J., and Linne, M.-L. (2019). Unique Features of Network Bursts Emerge From the Complex Interplay of Excitatory and Inhibitory Receptors in Rat Neocortical Networks. Front. Cell. Neurosci. *13*, 377. https://doi.org/10.3389/fncel.2019.00377.

Terrigno, M., Busti, I., Alia, C., Pietrasanta, M., Arisi, I., D'Onofrio, M., Caleo, M., and Cremisi, F. (2018). Neurons Generated by Mouse ESCs with Hippocampal or Cortical Identity Display Distinct Projection Patterns When Co-transplanted in the Adult Brain. Stem Cell Rep. *10*, 1016–1029. https://doi.org/10.1016/j.stemcr.2018.01.010.

Tremblay, R., Lee, S., and Rudy, B. (2016). GABAergic Interneurons in the Neocortex: From Cellular Properties to Circuits. Neuron *91*, 260–292. https://doi.org/10.1016/j.neuron.2016.06.033.

Watanabe, K., Kamiya, D., Nishiyama, A., Katayama, T., Nozaki, S., Kawasaki, H., Watanabe, Y., Mizuseki, K., and Sasai, Y. (2005). Directed differentiation of telencephalic precursors from embryonic stem cells. Nat. Neurosci. *8*, 288–296. https://doi.org/10.1038/nn1402.

Weihberger, O., Okujeni, S., Mikkonen, J.E., and Egert, U. (2013). Quantitative examination of stimulus-response relations in cortical networks *in vitro*. J. Neurophysiol. *109*, 1764–1774. https://doi.org/10.1152/jn.00481.2012.

Wonders, C.P., and Anderson, S.A. (2006). The origin and specification of cortical interneurons. Nat. Rev. Neurosci. *7*, 687–696. https://doi.org/10.1038/nrn1954.

Xue, M., Atallah, B.V., and Scanziani, M. (2014). Equalizing excitation–inhibition ratios across visual cortical neurons. Nature *511*, 596–600. https://doi.org/10.1038/nature13321.

Yokoi, R., Shigemoto-Kuroda, T., Matsuda, N., Odawara, A., and Suzuki, I. (2022). Electrophysiological responses to seizurogenic compounds dependent on E/I balance in human iPSC-derived cortical

neural networks. J. Pharmacol. Sci. *148*, 267–278. https://doi.org/10.1016/j.jphs.2021.12.006.

Zhang, Y., Pak, C., Han, Y., Ahlenius, H., Zhang, Z., Chanda, S., Marro, S., Patzke, C., Acuna, C., Covy, J., et al. (2013). Rapid Single-Step Induction of Functional Neurons from Human Pluripotent Stem Cells. Neuron *78*, 785–798. https://doi.org/10.1016/j.neuron.2013.05.029.

Druga R, Salaj M, Al-Redouan A. Parvalbumin - Positive Neurons in the Neocortex: A Review. Physiol Res. 2023 Jul 31;72(Suppl 2): S173-S191. doi: 10.33549/physiolres.935005. PMID: 37565421; PMCID: PMC10660579.

Izhikevich EM. Polychronization: computation with spikes. Neural Comput. 2006 Feb;18(2):245-82. doi: 10.1162/089976606775093882. PMID: 16378515.

Stem Cell Reports, Volume *20*

Supplemental Information

A proper excitatory/inhibitory ratio is required to develop synchronized network activity in mouse cortical cultures

Eleonora Crocco, Ludovico Iannello, Fabrizio Tonelli, Gabriele Lagani, Luca Pandolfini, Marcello Ferro, Giuseppe Amato, Angelo Di Garbo, and Federico Cremisi

Supplemental Figures

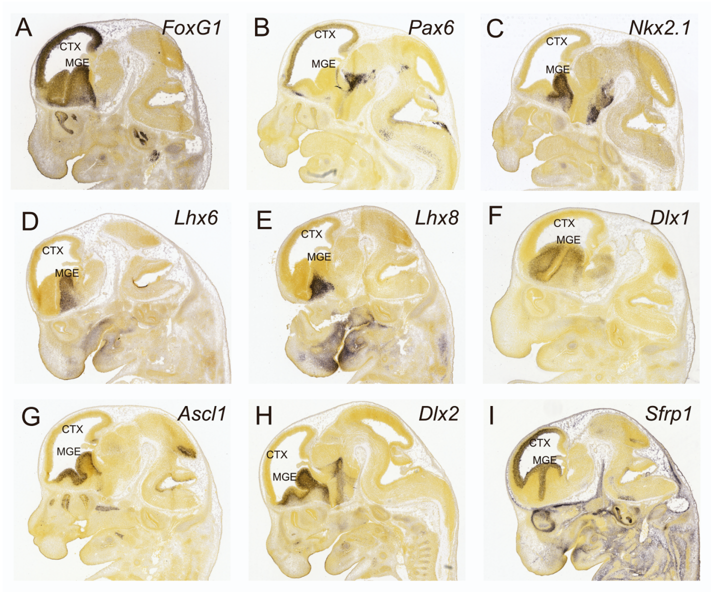

Figure S1. Positional markers of dorsal and ventral telencephalon. A-I) ISH of P13.5 mouse showing the expression of markers of telencephalic (A), dorsal (B), ventral (C) and subpallial (D-I) identity analyzed in Figure 1C-F. CTX: cortex; MGE: medial ganglionic eminence. Images from Allen Brain Atlas: Developing Mouse Brain (https://developingmouse.brain-map.org/).

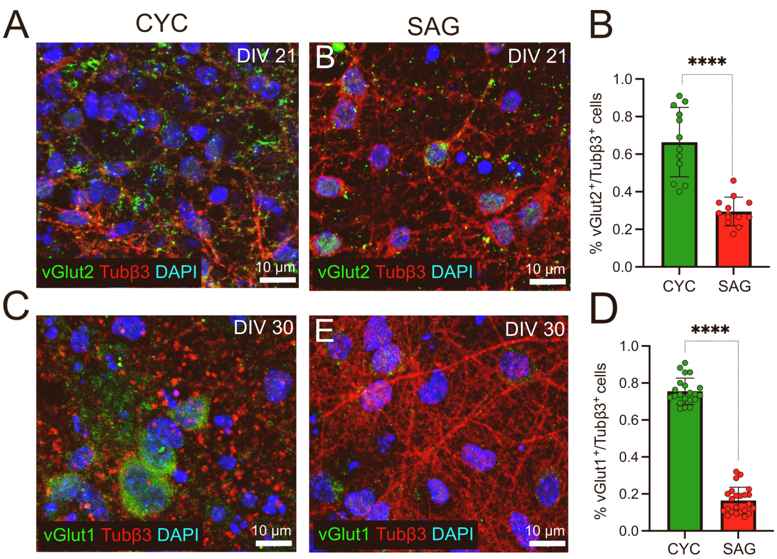

Figure S2. Glutamatergic marker expression in CYC and SAG cultures. A,C) Representative images of vGlut2⁺ and vGlut1⁺ neurons in CYC and SAG neurons at DIV21 and DIV30. B,D) Percentages of vGlut1 and vGlut2 positive neurons, respectively, in CYC and SAG cultures (n = 3 independent experiments). Positive cells were evaluated as Tubβ3 positive neurons surrounded by synaptic vesicles. Mean ± SD is shown, unpaired t-test, ****p-value < 0.0001.

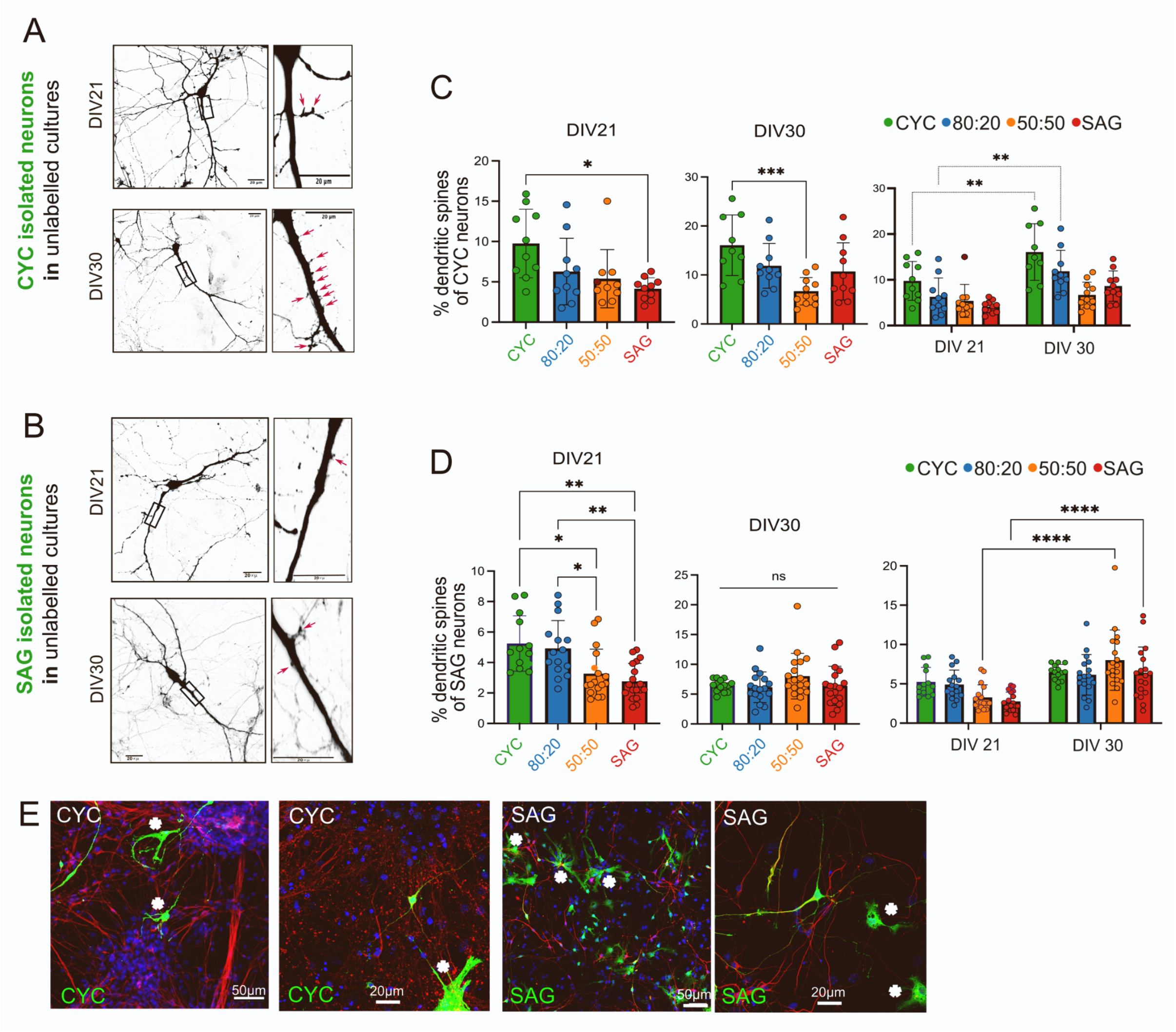

Figure S3. Spine analysis of CYC and SAG labeled neurons in pure and mixed CYC and SAG unlabeled cultures.

A,B) Representative images of dendritic branching of CYC and SAG EGFP-labeled neurons in pure and mixed cultures of unlabeled cells at DIV21 and DIV30. Insets on the right show enlarged details of the main image, with spines indicated by red arrows. Spines were identified according to Miura et al., (2020) using the Simple Neurite Tracer (SNT) toolbox (see Methods). C,D) Quantification of dendritic spines of CYC (C) and SAG (D) neurons at DIV21 and DIV30; mean ± SD is shown. Ordinary one-way ANOVA with Tukey's multiple comparisons test was performed to compare samples at each time point, and two-way ANOVA with Šídák's multiple comparisons test was performed for comparisons over time. N = 3 independent experiments; p-values: *p-value < 0.05, **p-value < 0.01, ***p-value < 0.001, ****p-value < 0.0001; ns = not significant. F) Examples of astrocytes (asterisks) labeled by the EGFP lentivector and not included in the analysis.

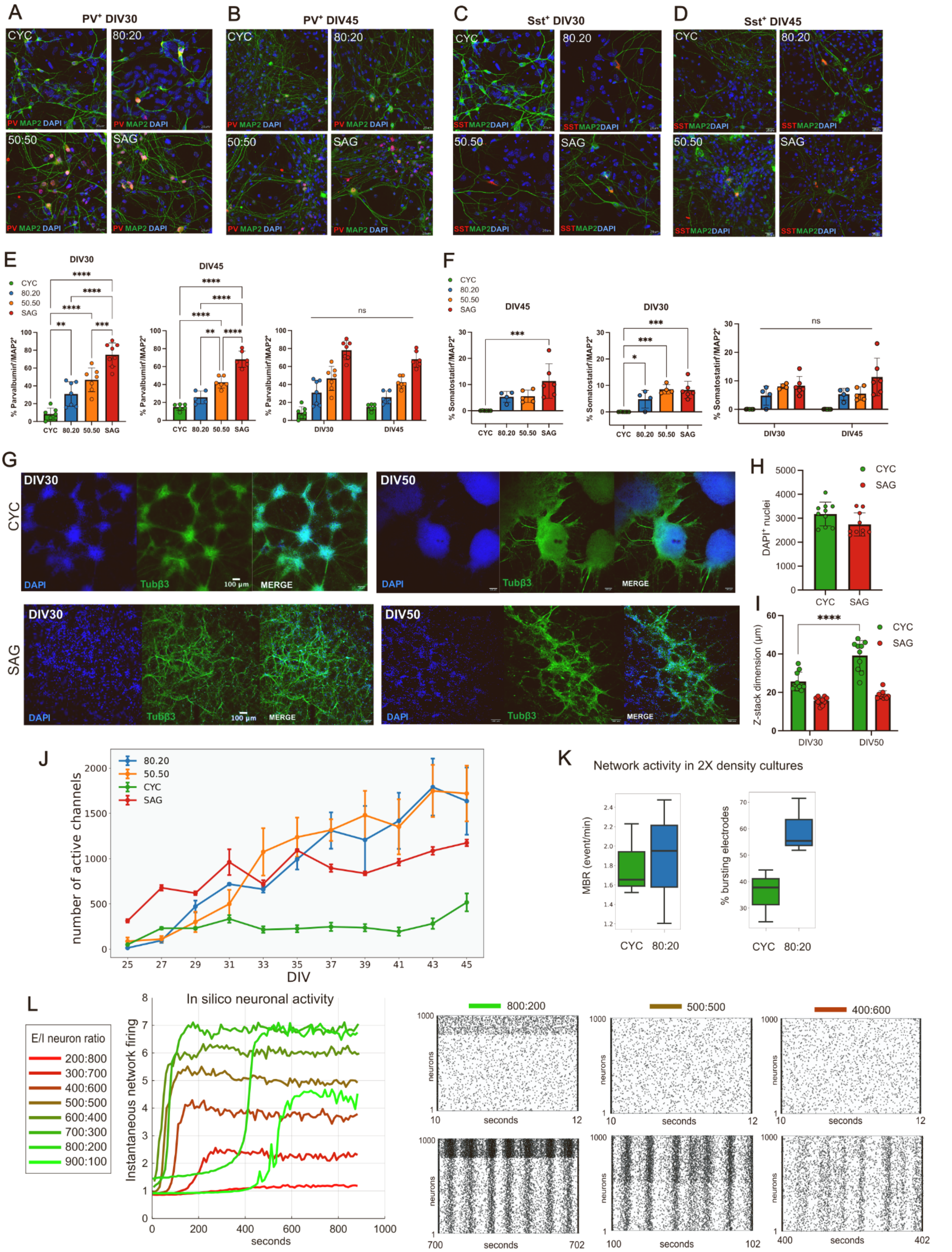

Figure S4. *In vitro* and *in silico* analysis of networks with different E/I balance. A–D) Representative immunodetection images of pure and mixed CYC and SAG cultures. Parvalbumin (PV) is shown in A and B, Somatostatin (SST) in C and D. Images were taken at DIV30 in A and C, and at DIV45 in B and D. E,F) Quantification of positive cells as in A-D); mean ± SD is shown; Ordinary one-way ANOVA with Tukey's multiple comparisons test was performed to compare samples

at each time point, and two-way ANOVA followed by Šídák's multiple comparisons test was performed for comparisons over time. P-values: **p-value < 0.01, ***p-value < 0.001, ****p-value < 0.0001, ns = not significant; (n = 3 independent experiments). G) Representative images of CYC and SAG cultures at DIV30 and at DIV50, showing morphological differences between the two cultures. H) Quantification of DAPI^{+} nuclei in each image (n = 3 independent experiments); mean ± SD is shown. I) Quantification of cluster size in CYC and SAG cultures (n = 3 independent experiments); mean ± SD is shown; two-way ANOVA followed by Šídák's multiple comparisons test was performed; ****p-value < 0.0001. J) Number of active channels in each condition over time (n = 3 independent experiments; mean ± SEM is shown). K) MBR and percentage of active electrodes at DIV 45 in CYC and 80:20 cultures seeded at double cell density (to be compared to cultures shown in Figure 4D,E). L) *In silico* modeling of network activity. Spiking activity is shown from a numerically integrated Izhikevich neuron network of 1000 neurons, varying the E/I neuronal ratio from 900:100 to 200:800. Each neuron formed 100 random connections with other neurons. The network incorporated axonal conduction delays, with excitatory synapses having random delays up to 20 ms and inhibitory synapses fixed at 1 ms. Excitatory synaptic strengths underwent Spike-Timing Dependent Plasticity (STDP), dynamically adapting based on the precise relative timing of pre- and postsynaptic spikes throughout the simulation. The total analysis duration was segmented into 10 seconds-bins and each neuron's instantaneous firing rate was determined within smaller 10 ms sub-bins, identifying bursts when the firing rate exceeded 10 Hz. Instantaneous Network Firing (INF) for each 10 seconds-bin was determined by averaging individual neuronal burst rates across all recorded channels. The evolution of INF (left) and raster plots (right) illustrate the network's evolving activity patterns as synaptic weights are continuously modulated by STDP.

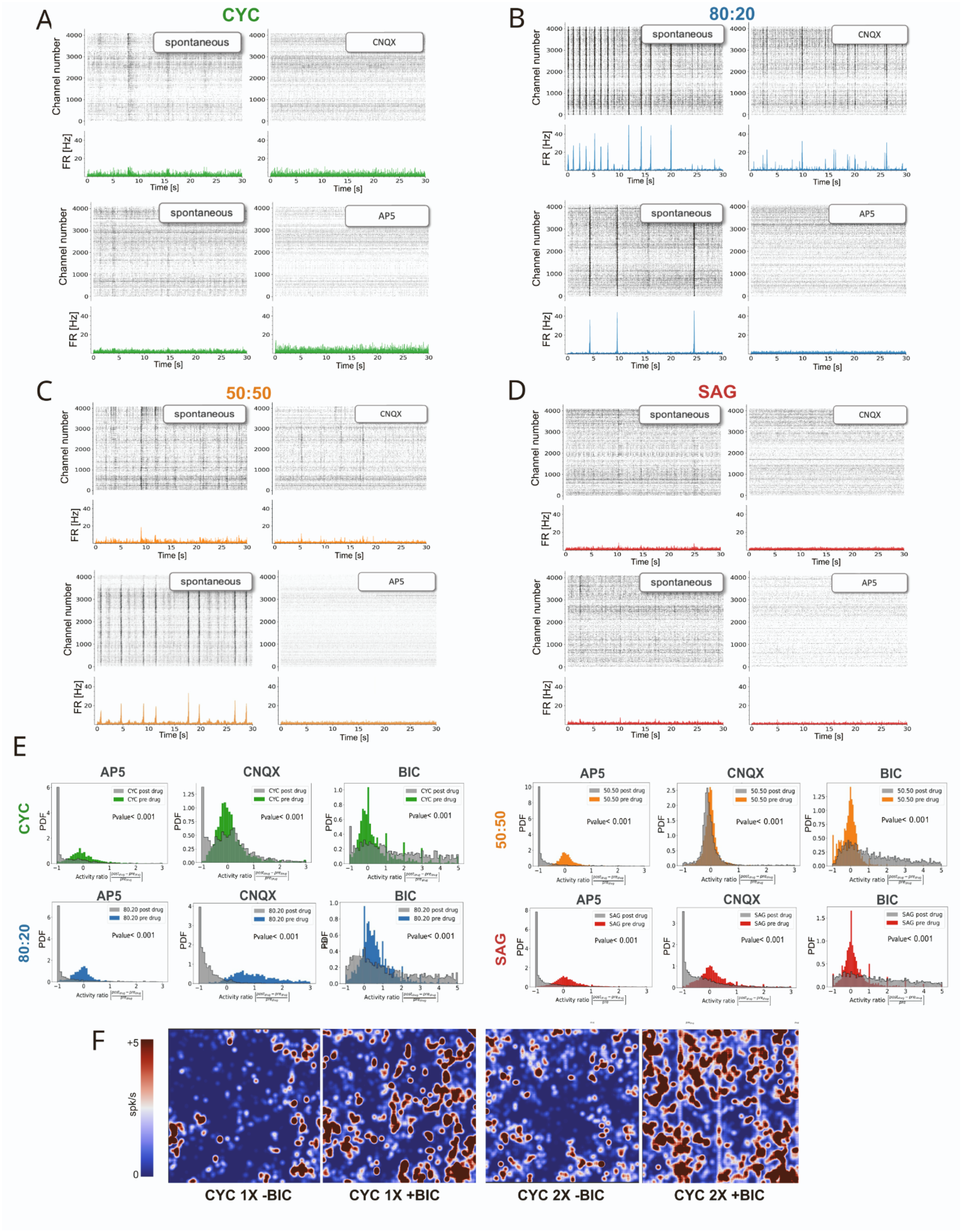

Figure S5. AP5, CNQX and Bicuculline significantly affect the firing activity in pure and mixed cultures. A-D) Representative raster plots for each culture condition showing the activity before and after drug administration (AP5 and CNQX). E) Probability distribution of the activity variation of each channel, before and after drug administration. The colored distributions represent the variation of baseline activity (without drugs) considering different time portions of spontaneous activity. The gray

distributions represent the variation after drug administration; Non-parametric Mann-Whitney U test between the baseline and post-drug distributions (p-values are displayed on the graph); PDF, probability density function. F) Heatmaps of global activity (5 minutes) of CYC cultures at normal (1X) or double (2X) cell density, before (-) and after (+) bicuculline (BIC) administration.

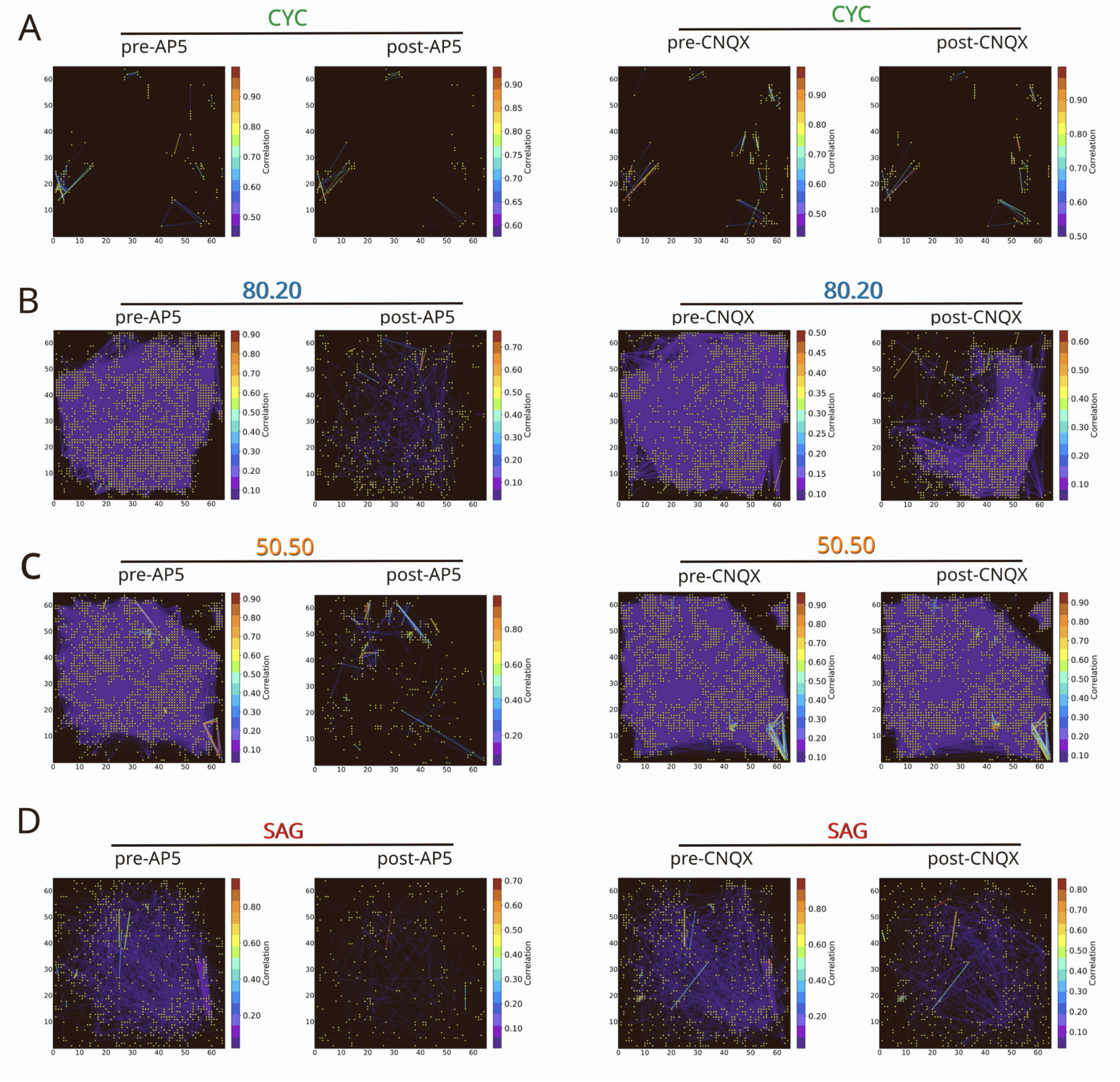

Figure S6. Representation of functional connectivity upon AP5 and CNQX administration. A-D) Connectivity plots of representative CYC (A), 80:20 (B), 50:50 (C) and SAG (D) cultures during spontaneous activity before (pre-drug) and after (post-drug) drug administration. Each yellow point represents a node of the functional graph; colored lines represent the correlation strength between two points (only the 10% of the functional links are shown). The color bar indicates the correlation index.

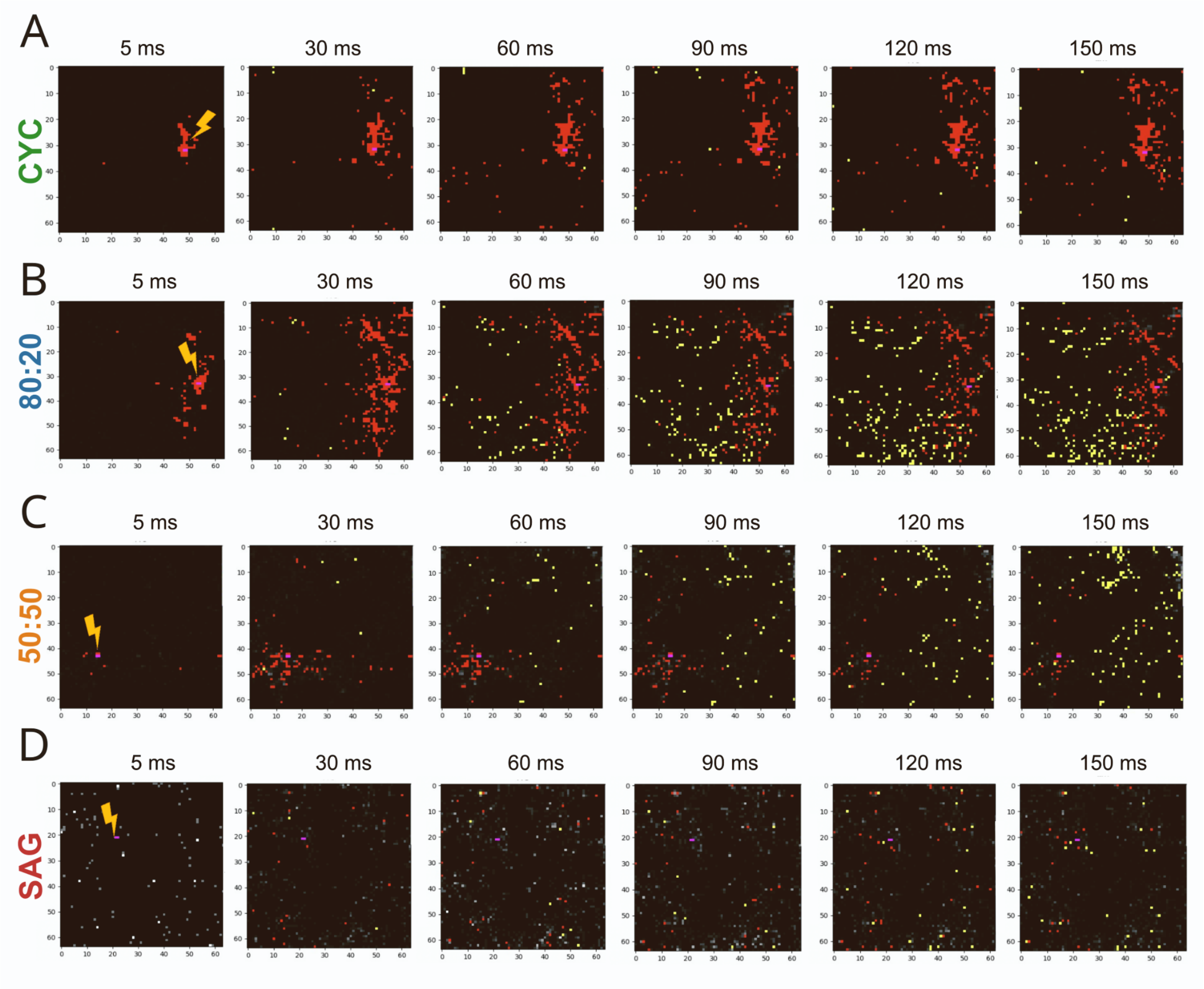

Figura S7. Schematic representation of single channel stimulation. A) Schematic representation of the chip plate, where each pixel represents a channel: purple pixels are two stimulated electrodes (cathode and anode), also indicated by the yellow lighting; white/grey pixels represent electrodes with baseline activity, red pixels represent channels that had a significantly increased modification as compared to the baseline activity (above the 95% CI), while yellow pixels are those with a significantly decreased modification as compared to the baseline activity (below 5% CI) (See Methods).

Supplemental information

Methods

Maintenance of mouse ES cells

Mouse embryonic stem cells (ES, clone E14Tg2A) were expanded and differentiated based on our previous method (Bertacchi et al., 2015) as follows. Cells were kept on 0,1% gelatin-coated culture dishes, seeding at a density of 50000 cells/cm^2 and splitting when at 70-80% of confluence. Cells were maintained in ES cell medium based on GMEM (Gibco, 11710035), containing 10% Fetal Bovine Serum (Euroclone ECS0180L), 2mM Glutamine, 1 mM Sodium Pyruvate, 100 U/ml Penicillin-streptomycin, 1mM Non-essential amino acids, 0.05mM β-mercaptoethanol. The medium was replaced daily.

ES cells were expanded in ES medium for three to four passages, then cultured in 2i+LIF medium, based on GMEM supplemented with 1x N-2 Supplement (Gibco, 17502001), 1x B-27 Supplement minus Vitamin A (Gibco, 12587010), 2mM Glutamine, 1mM Sodium Pyruvate, 1mM NEAA, 0.05mM β-mercaptoethanol, 1µM MEK inhibitor PD0325901 (Mirdametinib, Selleck Chemicals, S1036), 3µM GSK3 inhibitor CHIR99021 (Sigma-Aldrich, SML1046), and 10ng/mL recombinant mouse LIF (Silva et al., 2008).

Differentiation of ES cells to different cortical fates

Differentiation of ES cells into cortical neurons was performed as previously described (Bertacchi et al., 2015; Tonelli et al., 2025). For neural induction, a chemically defined minimal medium (CDMM) containing DMEM/F12 (Gibco, 11320033), 2mM Glutamine, 1mM Sodium Pyruvate, 0.1mM NEAA, 0.05mM β-mercaptoethanol, 1x N-2 Supplement, and 1x B-27 Supplement minus Vitamin A was used. The differentiation protocol started by culturing ES cells ($3x10^4$ cell/cm^2) onto 0,1% gelatin plastic coated dishes in 2i+LIF Medium for one day, marked as Day *in Vitro* -1 (DIV -1).

The next day (DIV0), the medium was replaced with Wnt and BMP double inhibition (WiBi) medium: CDMM with 2.5µM 53AH (Wnt pathway inhibitor, Cellagen Technology, C5324-2s) and 0.25µM LDN193189 hydrochloride (BMP inhibitor, Sigma-Aldrich, SML0559). Cells were cultured in WiBi medium for 3 days (DIV0 - DIV3). At DIV3, differentiating ES cells were dissociated and seeded (30000 cells/cm2) in CDMM on dishes coated with Poly-ornithine (PLO, Sigma; 4 µg/cm^2 in sterile water, 3 hours coating at 37°C) and purified mouse Laminin (msLam, Sigma-Aldrich, CC095-M; 1 µg/cm^2 in PBS, O/N coating at 37°C). Next day, the medium was changed to WiBi medium and cells were cultured until DIV5 with daily medium changes.

From DIV5 until DIV10 Cyclopamine (Sigma, S- 4116, 3µM) or Smoothened agonist SAG (Santa Cruz Biotechnology, SC-212905, 0.1µM) were added to WiBi medium to

differentiate the two neural populations. At DIV7 cells were split again onto mouse-Laminin coating dishes with a density of 110.000 cells/cm^2.
From DIV11 until DIV21 cells were maintained in “young” Neurobasal (yNb), containing Neurobasal medium, 2mM Glutamine, 1mM sodium Pyruvate, 0.05mM β-mercaptoethanol, 0.2mM Ascorbic Acid (Vit. C), and B-27 Supplement minus Vitamin A. From DIV13, half of the eNb medium was changed daily to allow conditioning of the medium by the differentiating neurons.
At DIV21, medium was changed to “old” Neurobasal medium (oNb), containing Neurobasal-A (Gibco, 10888022), 2mM Glutamine, 1mM sodium Pyruvate, 0.05mM β-mercaptoethanol, 0.2mM Ascorbic Acid (Vit. C), B-27 Supplement 50x (Gibco, 17504044), and 20 ng/ml recombinant human BDNF protein (Novus Biologicals, NBP2-52006). The oNb medium was changed every 2 to 3 days to condition the medium and allow the differentiated neurons to mature.

Passaging and long-term culture of neurons
Cells were passaged during differentiation to avoid overgrowth and hypoxia, last time at DIV22 for long term experiments. Cells were first washed with 1x Versene and then incubated with 1x Trypsin for 5-20 min at 37°C. When most of the cells detached, trypsin was inactivated by adding 20% FCS to the cell suspension and diluting in warm PBS (1:5 ratio).
The cell pellet was rinsed with a warm yNb medium containing 4µM of Rock inhibitor Y-27632 (Cell Guidance Systems, SM02) to reduce the mortality of post-mitotic neurons. Cells were seeded on PLO/msLam-treated glass (200000 cells/cm2) or sterilized high-density microelectrode array (HD-MEA Accura, 3Brain) (60000 cells/chip) and allowed to adhere O/N. The next day, the medium was changed, and the Rock inhibitor was removed. Occasionally, 1.5µg/mL msLam was added to the medium to increase neuron attachment and long-range projection.
To prepare the mixed cultures of 80:20 and 50:50, neurons were splitted at DIV22.

Lentiviral vectors transduction
Lentiviral vectors were prepared by transfecting HEK293T cells O/N with Lipofectamine 2000 (Invitrogen, 11668019) and a DNA mixture according to the manufacturer's protocols. The DNA mixture consisted of the lentiviral vector of interest together with the psPAX2 packaging (Addgene #12260), the pCMV-VSV-G envelope (Addgene #8454), and the pCMV-Rev(NL4.3) (Addgene #115776) expressing plasmids in a 4:3:1:1 ratio. Transfection medium was discarded the next morning, and viral particles were collected 48 hours later and used immediately or frozen at -80°C. To achieve a high rate of cell transduction, lentiviral vectors were used fresh in a 1:1 ratio with the culture medium along with 8µg/mL Polybrene (Sigma-Aldrich, TR-1003), and cells were typically transduced O/N after passaging to increase lentiviral access to the cell surface. The lentiviral vectors used in this work consisted of the pWPXLd lentiviral backbone (Addgene #12258) containing the EGFP coding sequence. To clone the EGFP reporter with PuroR sequence, the EGFP enhancer construct was amplified by PCR using a forward and reverse primer

carrying a MluI and EcoRI restriction site, respectively (forward: actacgggatccaggcctaagcttACGCGT; reverse: tagctagctactaGAATTCgagatctgagt). The vector carrying EGFP was constructed replacing the original EF1α promoter and PuroR sequence in the pLV-EF1α-IRES-Puro vector (Addgene #85132) with the amplicon carrying the EGFP reporter using the MluI/EcoRI restriction sites. The ligated vector was then sequenced to ensure correct cloning of the reporter.

Immunofluorescence (IF) analysis

All cell cultures were fixed with PFA solution (2%), incubated at RT for 9 minutes, followed by aspiration and 3 washes with 1XPBS at room temperature (RT). Cells were then permeabilized and blocked in 3%FCS, 3%BSA (Blocking Buffer solution) + 0.3% Triton at RT for 1 hour. The permeabilization buffer was then aspirated and replaced with a primary antibody solution in Blocking containing 0,1% Triton and the corresponding dilution in the table S1 below. Primary antibody solution was incubated at 4°C overnight. The next day, the primary antibody solution was removed, and cells were washed 3 times with 1XPBS at RT.

The secondary antibody solution was supplemented with corresponding anti-(host) secondary antibodies in the Blocking Buffer solution, diluted at 1:1000, and cells were incubated for 1 hour at RT. Secondary antibody solution was removed, and cells were washed 3 times with 1XPBS at RT. DAPI was added to the last 10’ PBS wash, diluted 1:10.000. After the final PBS wash, all PBS was aspirated, and cells were mounted in Aqua/Poly-mount (Polysciences, 18606-100) for confocal imaging.

Images were produced on a Leica Stellaris 5 or Zeiss LSM 900 confocal microscope, by acquiring z-stack images 10-15 optical slices thick at 40x or 63x magnification.

Table S1. Primary,secondary antibodies and chemicals used in this section:

**Antibody**	**Dilution**	**Host**	**Company**	**Catalog no**
GFP	1:3000	Chicken	Aves	GFP-1020
Tubulinβ3	1:10000	Mouse	BioLegend	801202
PARVALBUMIN	1:1000	Guinea pig	Synaptic Systems	AB-2156476
SST	1:500	Rat	Millipore	MAB354
vGLUT2	1:1000	Rabbit	Synaptic Systems	135403
vGLUT1	1:1000	Rabbit	Invitrogen	482400
MAP2	1:6000	Chicken	Novus	NB300-213
vGAT	1:500	Guinea pig	Synaptic Systems	131011C2
GFAP	1:1000	Mouse	Sigma-Aldrich	MAB360

SATB2	1:200	Mouse	Santa Cruz Biotechnology	sc-81376
Tbr1	1:400	Guinea pig	Synaptic System	328005
Pax6	1:400	Rabbit	Covance	PRB-278P
Nkx2.1	1:400	Rabbit	Abcam	ab76013
FOXG1	1:500	Rabbit	Abcam	ab18259

**Antibody**	**Fluorophore**	**Company**	**Catalog no**
DONKEY Anti-Mouse IgG	Alexa fluor 546	Invitrogen	A10036
GOAT Anti-Rabbit IgG	Alexa fluor 488	Abcam	AB150077
GOAT Anti-Chicken IgG	Alexa fluor 488	Invitrogen	A11039
GOAT Anti-Guinea Pig IgG	Alexa fluor 488	Invitrogen	A11073
GOAT Anti-Rabbit IgG	Alexa fluor 546	Invitrogen	A11035

**Chemicals**	**Company**	**Catalog no**
biotinylated Wisteria Floribunda Lectin (WFA)	Vector Laboratories	B-1355-2
Streptavidin, Alexa Fluor™ 488 conjugate	Thermo Fisher	S11223

Quantification of Neurite Branch Length and Spine Number Using SNT

Images were acquired with an HC PL APO 63x/1,40 OIL CS2 objective lens and quantification of neuronal morphology, including neurite branch length and spine number, was performed using the Simple Neurite Tracer (SNT) toolbox (v3.1.114), implemented within the Fiji distribution of ImageJ (Arshadi et al., 2021). Quantification of images was conducted under a double-blind protocol. Images were imported into Fiji and preprocessed as necessary to ensure optimal contrast for tracing (e.g., background subtraction, channel separation for multi-channel images). Neuronal processes were traced using SNT's semiautomated pathfinding algorithm. Centerlines of dendritic and axonal arbors were generated from intensity thresholded images, with tracing supported across multi-dimensional image stacks. After completion of the tracing procedure, quantitative morphometric analysis was carried out using SNT's built-in metrics. Individual branch segments were defined between bifurcation or termination points, and total cable length was computed per cell.

Spine detection and quantification were performed from EGFP-labeled fluorescent images for dendritic morphology (Miura et al., 2020). The analysis was performed observing the morphology of different types of spines according to the literature (Harris KM et al., 1992; Kuo et al., 2023). Spot density (number of spines) was computed for each traced segment and aggregated per cell. Tracing data were stored in SNT's traces format and exported in CSV format for compatibility with downstream analysis tools. Statistical comparisons between conditions were performed using ANOVA with Tukey's multiple comparisons test.

Quantification analyses of the density of glutamatergic and GABAergic vesicles

This analysis was performed using the ImageJ Synapse Counter plugin (https://github.com/SynPuCo/SynapseCounter). To determine the density of synaptic puncta per fiber, vGlut1, vGlut2, and vGat positive puncta were divided by the total area of the Tubβ3 fluorescent signal in each field (see Figure 2). Moreover, the counting of the percentage of vGlut1 and vGlut2 positive cells was calculated by dividing the percentage of nuclei surrounded by synaptic vesicles by the number of neurons (Tubβ3 positive cells) (see Figure S2) (Kempf et al., 2021).

For the analysis of cell clusters in CYC and SAG cultures at DIV30 and DIV50 (Figure S4), the number of DAPI positive cells was calculated using ImageJ's "Analyze Particles" feature for each image (confocal images taken with a 10x objective).

Quantification was performed on three independent experiments and on selected fields for each sample. Statistical significance was assessed using one-way ANOVA and Student's t-test followed by Tukey's multiple comparison test after testing for normality and lognormality. To compare the two time points, statistical significance was assessed using two-way ANOVA with Šídák's multiple comparisons test.

RNA extraction and qRT-PCR analysis

Samples for RNA extraction were harvested following the same protocol used for splitting. After the centrifugation step, the supernatant was removed, and the cell pellet was processed using the NucleoSpin® RNA kit (Machery-Nagel, 740955.250). RNA concentration was measured with the NanoDropTM Lite Spectrophotometer. For each RNA sample, approximately 200 ng of RNA were reverse transcribed into cDNA for qRT-PCR analysis using the Reverse Transcriptase Core Kit 300 (Eurogentec RT-RTCK-03). 8 µL of cDNA were then mixed with SensiFAST SYBR mix (12 ml, BioLine BIO-98020) and the amplification analyses were quantified with Qiagen 72- Well Rotorgene (Corbett).

The Relative Expression method supplied with the software of the Rotorgene device was employed. The CT for each gene was obtained directly from Rotorgene. An internal control was used to reduce the variability caused by possible changes in the amount of RNA/DNA between each sample, following the ΔCT analysis method (Pfaffl, 2001). β-actin was used as the reference gene. The PCR efficiency of each

sample was raised to the ΔCT to obtain the fold change of the target gene relative to the expression of β-actin (which expression was set to 1 with this method).

Table S2. qRT-PCR primer sequences.

GENE OF INTEREST	FORWARD PRIMER	REVERSE PRIMER
βActin	AATCGTGCGTGACATCAAAG	AAGGAAGGCTGGAAAAGAGC
FoxG1	CGACCCTGCCCTGTG	GGAAGAAGACCCCTG
Nkx2.1	CAATGAGGCTGACGC	GAAGTGGGTTTCCTG
VIP	GCACCAGCAGGCAGTAACAG	ACAAGGAGCTGGGCCTTATT
Sst	TCGCTCTAAGTCTCACTCGCC	CGCTCCCATATTTCAGCCAC
Pvalb	TCTTTTCGCACTTGCTCTGC	CCTTCTTCACCTCATCCGGG
Ascl1	GCCCGAATCACAGATGGGTT	ATCAACCCAGTTTCAGGGGG
Dlx2	TCCTACTCCGCCAAAAGCAG	GGAGTAGATGGTGCGTGGTT
Pax6	CCTCCTTCACATCAGGTTCC	CATAACTCCGCCCATTCACT
Lhx6	CGGCCTGATGGATCTCACTG	CTGGGCCATCACCTGCAAT
Lhx8	AAACACGTCAGTCCCAACCA	ACGTAGGCAGAATAAGCCATTT
Dlx1	GGTTTCTGGGGCGGGAAGCG	GGAGCGGGACGCACAATGGG
Sfrp1	CTGCCTCCTGCATGTGTGTA	TCTGGATGGGCTTTTCGCTT

Electrophysiological recordings and analysis

Neuronal cultures (DIV22) were seeded for recordings at a density of $5*10^4$ cells/MEA chip in all experiments, except control experiments with double density in which 10^5 cells/MEA chip were seeded. Cells were cultured onto commercially available Accura HD-MEA chips (3Brain GmbH), each equipped with 4096 CMOS microelectrodes with 60µm pitch and 21×21µm size, allowing recording of extracellular local field potentials. The 4096 electrodes are arranged in a 64×64 grid of 3.8 x 3.8 mm. Electrophysiological recordings were performed on DIV25-45 using the BioCam DupleX system (3Brain GmbH). After a 5 min acclimation period outside the incubator, spontaneous neuronal activity was recorded for 5 min under stable conditions (37°C, 5% CO2) and sampled at 20kHz. Spike detection was performed using BrainWave 5 software (3Brain GmbH; see next paragraph). Finally, chemical stimulation was induced by adding specific compounds to the medium: Bicuculline (BIC, 20µM; Sigma-Aldrich, 14340) to block GABA receptors, D-2-amino-5-phosphonopentanoic acid (AP5, 25µM; Sigma-Aldrich, A8054) to block NMDA receptors, and 6-cyano-7-nitroquinoxaline-2,3-dione (CNQX, 25µM;

Sigma-Aldrich, C127) to block AMPA receptors. Electrophysiological activity was recorded 5 min before and 6 min after drug administration.

Protocol of stimulation

We stimulated all the types of cultures with electrical stimulation of one pair of electrodes (for single stimulation) or on seven pairs of electrodes (for multiple stimulations) that had the highest Firing Rate (>5 spks/sec). We used a biphasic stimulus with the following parameters: current amplitude of 10 μA per electrode, with a duration of 100 μs (50 % duty cycle) and an interphase delay of 10 μs. We applied a protocol of 25 stimuli at 0,1 Hz. To evaluate network-induced activity, we performed network burst (NB) detection and classified a network burst as electrically induced if the absolute difference between its start time and the stimulus time was less than twice the time bin used in the NB detection algorithm. Then we computed the temporal duration for the spontaneous and the induced NB.

Dispersion Index and Distribution of the global response to the stimulus

The results of dispersion index and global response were based on the computation of temporally aggregated neural activity in response to a given stimulus.

When a stimulation pulse was delivered, electrical activity was recorded from every other MEA electrode. A spike sequence was then extracted by means of a spike detection algorithm (described in the following subsection).

Some spurious spikes and electrical artifacts were removed by means of a filtering algorithm, removing spike signals with amplitude $|V|_{max}$ above a threshold $V_{thr} = 1000\, mV$. Another filtering algorithm calculates the area under the spike curve A and divides it by the spike amplitude in order to obtain an estimate of the spike temporal width, and filters out the spike when this value is above another threshold $W_{thr} = 25us$:

$$|V_{max} - V_{min}| > V_{thr}\ , \quad \frac{A}{|V_{max} - V_{min}|} > W_{thr}$$

The response to a stimulus observed on a given electrode is defined as the difference in spike count between a time window immediately after the stimulus instant, and one immediately preceding it. The window size W is chosen to range from 5 to 150 ms. Mathematically, the response r_i over electrode i (i=1, 2, …, 4096) can be expressed as:

$$r_i^{(t_s)} = \sum_{t=t_s}^{t_s+W} s_i(t) - \sum_{t=t_s-W}^{t_s} s_i(t)$$

Where t_s denotes the stimulus delivery time, and $s_i(t)$ is the spike signal recorded from electrode i, i.e. a function that takes value 1 when t corresponds to the time of a spike event, and 0 everywhere else.

During a recording session, stimulations were repeated multiple times, in order to account for statistical variability. Therefore, from the recordings, multiple values of the response variable over each electrode are obtained. Calling the stimulation

instants t_0, t_1, …, t_k, it is possible to obtain responses $r_i^{(t\_0)}$, $r_i^{(t\_1)}$, …, $r_i^{(t\_k)}$. We used k=25 in our experiments. Each of these values can be seen as a realization of a random variable R_i, of which we wish to provide a statistically grounded estimate. This is by computing the sample mean and the corresponding confidence intervals from the observations $r_i^{(t\_0)}$, $r_i^{(t\_1)}$, …, $r_i^{(t\_k)}$:

$$\hat{R}_i = \frac{1}{k} \sum_{s=0}^{k} r_i^{(t_s)}$$

Concerning the confidence intervals, initially we employed both a parametric and a nonparametric estimator. However, we found the non-parametric estimator to be ill suited for scenarios with very few spikes in the time window, so we resorted to the parametric estimator instead. This estimator is simply based on the T-Student estimation for the confidence intervals of the sample mean:

$$CI_\alpha^{+}, CI_\alpha^{-} = \hat{R}_i \pm t^{k-1}_{1-\alpha/2} \frac{\sigma_i}{\sqrt{k}}$$

where σ_i is the sample standard deviation of the observations $r_i^{(t\_0)}$, $r_i^{(t\_1)}$, …, $r_i^{(t\_k)}$, and $t^{k-1}_{1-\alpha/2}$ is the required T-Student's percentile.

Each pixel of the matrices in Fig. S7 represent the corresponding value $\hat{R}_i$ measured for each electrode i, and represented graphically in grayscale: darker colors represent weaker responses, while brighter colors correspond to stronger responses. Moreover, when the measured confidence intervals indicate that the response value of a given pixel is significantly above zero in a statistical sense, then the corresponding pixel is colored in red. Specifically, the required confidence level is set to 95%. Similarly, when a confidence level indicates that the response of an electrode is significantly below zero, the pixel is denoted in yellow. In this case we require the positive tail of the distribution to be below zero at the 100 - 95 = 5 percentile.

Finally, the dispersion index in Fig. 7 is obtained by considering the geometric dispersion of the electrodes with significant response to a stimulus. Ideally, if all the response is concentrated in a very localized portion of the MEA grid, the corresponding dispersion should be low, while if the response is spread out all over the grid, the dispersion is large. More formally, the dispersion index is computed by considering the positions of the significantly responding electrodes over the grid, represented as x-y coordinates in the domain [0, 1]x[0, 1] (where coordinates 0, 0 denote the top-left corner, and 1-1 denote the bottom right corner of the MEA grid).

Let's denote with (x_i, y_i) the coordinates of electrode i, and let R = {i_1, i_2, …} be the set of significantly responsive electrodes. It is possible to evaluate the distance between any pair of such electrodes as

$$d_{a,b} = \sqrt{(x_a - x_b)^2 + (y_a - y_b)^2}$$

This can be again considered as a realization of a random variable D, which depends on the particular pair a, b that was chosen. It is also possible to obtain

several samples of D by selecting all possible electrode pairs from R. From all these samples, we can once more evaluate the sample mean D, and the corresponding confidence intervals, for statistical comparisons.

$$\hat{D} = \frac{1}{k} \sum_{a,b} d_{a,b}$$

$$CI_{\alpha}^{+}, CI_{\alpha}^{-} = \hat{D}_i \pm t^{k-1}_{1-\alpha/2} \frac{\sigma}{\sqrt{k}}$$

In this case, k=$|R|(|R| - 1)/2$, and σ is the sample standard deviation of the observations $d_{a,b}$.

The parametric estimation methods discussed above are based on the central limit theorem, and rely on the assumption of variance finiteness in the random variables being statistically estimated. This assumption was checked by observing the homoscedasticity of the observations, i.e. sample variance converging stably as the number of observations increased.

Spike and Burst detection

The spike detection algorithm used is the PTSD (precision time spike detection) (Maccione et al., 2009) that requires 3 parameters:

- Noise threshold (set to 10 times the standard deviation of the baseline noise for each channel individually).
- Peak lifetime period (set to 2 ms), corresponding approximately to the spike duration.
- Refractory period (set to 2 ms), which corresponds to the minimum time interval between one spike and the next (Parodi et al., 2023).

After spike detection, each channel burst was detected based on the channel spike train. The burst detection was performed as proposed in the literature (Chiappalone et al., 2005). The implemented algorithm requires 2 parameters: the maximum inter-spike interval (ISI) between two consecutive spikes of a burst (*maxISI*, set to 50 ms) and the minimum number of spikes in a burst (*minspk*, set to 5 spikes).

Spike bursts are defined as sequences of spikes with ISI smaller than *maxISI* and containing at least a number of spikes equal to *minspk*. The values of 50 ms and 5 spikes for these two parameters were set after a series of comparisons between the results of the burst detection algorithm and visual inspection of various experimental recordings. Mean firing rate (MFR) and mean burst rate (MBR) were calculated by counting the average number of spikes or bursts in 5 minute recordings divided by the number of active channels (firing rate greater than 0.1 spikes/s for spiking activity and burst rate greater than 0.3 bursts/min for bursting activity). To fully characterize the bursting activity, the mean burst duration (MBD), the percentage of bursting electrodes and the percentage of random spikes were calculated. The mean burst duration (MBD) was calculated as the average temporal length of the bursts. The percentage of bursting electrodes was determined as the proportion of active channels exhibiting bursting activity. Lastly, the percentage of random spikes was computed by considering all spikes that were not part of any burst activity. All these

quantities were calculated for all replicates of the same culture and the Mean ± SEM was plotted over time (Figure 4).

Network Burst activity

Network bursts (NBs) are events of collective synchronization within the culture. To quantify the level of synchronization of the neuronal network activity, we derived the mean network burst rate (NBR, number of network events per minute) and the network burst duration (NBD) for each culture. A NB is identified when the activity is composed of at least 50 consecutive spikes within a 50 ms window and the firing rate (number of spikes per bin) exceeds a threshold determined from the mean and standard deviation of the firing rate signal. NB detection was performed as follows:

- The network's firing rate (number of spikes per bin) was calculated using a time bin of 50 ms.
- Firing rate peaks were considered if the local firing rate maxima were greater than the mean of the firing rate signal plus 4 times the standard deviation.
- The beginning and end of each NB were defined such that the firing rate before and after each peak fell below the mean of the firing rate signal plus 2 times the standard deviation (in cases where two or more adjacent local maxima correspond to the same onset, only one event is detected).

NBR was calculated for all replicates of the same culture and the Mean ± SEM was plotted over time.

Center of Activity Trajectories (CATs)

To quantify the propagation of coordinated network activity, we performed Center of Activity Trajectory (CAT) (Chao et al., 2007) analysis, which calculates the spatial and temporal evolution of each NB event. CAT provides a sort of center of mass for spikes, where the location of the center of mass is the physical location of the channels in the MEA map, and mass is replaced by spike activity.

The algorithm to compute NB trajectories is as follows: firstly, the start and end points of a NB are detected (as explained in the previous section) to define the time window of interest. All active channel spikes are then considered to compute the neuronal activity trajectory in this time window. A time step $\delta_t = 20$ ms, is fixed for the binning of neuronal firing. Then, the activity is computed by counting all spikes for each channel in each time window $[t, t + \delta_t]$. Finally, the activity trajectory is defined by : $CA(t) = \frac{\sum_{ch} A_{ch}(t)(row_{ch}, col_{ch})}{\sum_{ch} A_{ch}(t)}$, where $A_{ch}(t)$ represents the activity (counted spikes) of the channel ch at time t (in the corresponding time window $[t, t + \delta_t]$) and (row_{ch}, col_{ch}) are the physical coordinates in the MEA map (row and column) of the channel ch.

For each NB, we computed the spatial and temporal evolution of its activity trajectory throughout the duration of the synchronized event. The time evolution (with a time window of 150 ms for each NB) is represented in the plots by a colored scale, while the onset of each NB is highlighted by a blue dot.

Network connectivity analysis

To estimate the functional connectivity within the neural networks, we used a previously validated cross-correlation-based approach (Ullo et al., 2014), dealing with point processes of events (e.g., spike trains). More precisely, for each pair of active electrodes $\{x,\ y\}$, the cross-correlation function (cross-correlogram) between their spike trains was estimated as:

$$C_{x,y}(\tau) = \frac{1}{\sqrt{N_x N_y}} \sum_{s=1}^{N_x} \sum_{t=t_s-\frac{\Delta_t}{2}}^{t_s+\frac{\Delta_t}{2}} x(t_s) y(t + \tau)$$

where N_x, N_y are the total number of spikes for channels x and y respectively. The time bin Δ_t was set to 1 ms and the time delay tau (τ) was varied in a range from -2 to 2 ms. The absence of delay (τ = 0 ms) was excluded to eliminate synchronous spikes less than 1 ms apart, which are incompatible with the synaptic time delay (Poli et al., 2015). For each pair of channels, the maximum correlation value at delay τ was considered. To determine which correlations are statistically significant, it is necessary to select a threshold. We implemented a shuffling procedure that randomizes the temporal order of events to create a null hypothesis scenario where all observed correlations are purely due to chance as described in Tonelli et al., 2025.

The connectivity matrix of the randomized spike trains is computed, and the threshold is determined by computing the 99.9th percentile of the random correlation distribution. From the connectivity matrix, we derived the adjacency matrix, where the strength of the connections for the graph is represented by the correlation values (Figure 6A). Connectivity graphs were plotted while maintaining the physical position of the channels in the MEA map, with each connection colored based on the correlation value. From these graphs, we extracted the number of nodes and links (Figure 6).

Characterization of neuronal activity after chemical drug administration

To evaluate the effect of chemical drugs on the five different cultures, we computed the probability distributions of single channel activity variation before and after drug administration. For each culture, we computed the number of spikes per channel, considering all active channels under normal and drug conditions. The drug condition was considered excluding the first 100 seconds of recording after the moment of administration. The activity variation was calculated as the differences between the channel activity after and before drug administration (number of spikes per channel), divided for the channel activity before administration. To evaluate the real effect of the drugs, for each replicate of the same culture, we calculated the variation of the baseline activity in normal condition, considering different time portions of spontaneous activity (excluding 100 seconds of recordings between one portion and another). The distributions of firing rate variation account for the activity of individual

channels across all replicates. Finally, a non-parametric Mann-Whitney U test was performed to determine statistically significant differences between the baseline and post-drug distributions. Furthermore, to quantify the changes in the functional connectivity before and after drug administration, we computed the variation of the number of nodes and links compared to the baseline conditions (Figure 6C).

Neural network simulation

We implemented a published neuronal network model that establishes random connectivity with distinct populations of excitatory and inhibitory neurons, explicitly modeling axonal conduction delays and implementing Spike-Timing Dependent Plasticity (STDP) (Izhikevich, 2006). Neural network simulation was performed in the Matlab environment. The neural network was constructed with a total of 1000 neurons, varying the number of inhibitory cells, from 100 to 900. The dynamic behavior of each individual neuron was modeled using the Izhikevich formalism. For excitatory neurons, the recovery variable time scale was set at 0.02, with an after-spike reset value for the recovery variable of 8. Inhibitory neurons, in contrast, were characterized by a faster recovery variable time scale of 0.1 and a smaller after-spike reset value of 2 for the recovery variable. All neurons were initialized with a membrane potential of −65 mV, and their recovery variable was set to 0.2 times this initial membrane potential. A uniform spike threshold of 30 mV was applied across all neurons.

To stick to the Izhikevich formalism, network connectivity was established such that each neuron formed 100 synaptic connections with randomly selected target neurons. Synaptic weights were initialized differently based on the presynaptic neuron type: excitatory synapses began with a weight of 6, while inhibitory synapses were set to a fixed weight of −5. Importantly, excitatory synaptic weights were subject to a maximum constraint of 10 and a minimum of 0, and their values evolved over time through a mechanism of Spike-Timing Dependent Plasticity (STDP). In contrast, inhibitory synaptic weights remained constant throughout the simulation. To introduce biological realism, axonal conduction delays were incorporated into the model. Excitatory connections were assigned delays randomly drawn from a uniform distribution spanning 1 to 20 milliseconds. All inhibitory connections, reflecting their typically faster local influence, were assigned a fixed conduction delay of 1 millisecond. These values differ from those used by biological networks because in Izhikevich's formalism they proved optimal for modelling network activity.

The network received a continuous stochastic external drive, modeled as a random thalamic-like input. At each millisecond of the simulation, a single neuron was randomly selected to receive an excitatory current of 20 units, ensuring persistent but non-targeted external stimulation. This served to stimulate background activity within the network. The core of the network's learning capability lay in its implementation of STDP, which modulated excitatory synaptic strengths based on the precise relative timing of pre- and postsynaptic spikes. Upon a neuron's firing, its STDP trace was immediately incremented by 0.1, with this trace decaying

exponentially by a factor of 0.95 per millisecond between subsequent spikes. The synaptic weight derivatives accumulated based on these STDP traces, incorporating a scaling factor of −1.2 for presynaptic effects. At the completion of each simulated second, the excitatory synaptic weights were updated following the rule: se(t+1)=max(0,min(10,0.01+se(t)+sd(t))), where 0.01 was a small constant preventing excessive weight decay. Concurrently, the accumulated weight derivatives themselves decayed by a factor of 0.9 at the end of each second.

The simulation was carried out for a total duration of 15 minutes (900 seconds). The Izhikevich neuron model equations were numerically integrated using an Euler method, effectively employing a 0.5 ms time step for the membrane potential update, applied twice per millisecond simulation step. To ensure reproducibility of the simulation results, a specific random seed was set at the outset of the script. All occurrences of neural spiking, including the time of the spike and the identity of the firing neuron, were meticulously recorded and subsequently stored in a .mat file for comprehensive post-simulation analysis.

Network activity analysis was performed by analyzing spikes across multiple channels, with spikes binned into a predefined time interval of 10s, to estimate firing rates. Since the Izhikevich network does not generate burst dynamics with metrics directly comparable to biological networks, we employed an alternative metric that best approximates the average bursting activity observed in biological systems. We calculated Instantaneous Network Firing (INF). The burst rate for each neuron was quantified by first segmenting the activity within 10-second analysis bins into smaller, 10-millisecond sub-bins. Within these sub-bins, the instantaneous firing rate for individual neurons was computed. Bursts were then identified as discrete periods where the instantaneous firing rate exceeded a threshold of 10 Hz. The number of such burst events per second was subsequently calculated for each neuron. Finally, INF for each 10-second bin was determined by averaging these individual neuronal burst rates across all recorded channels.

Quantification and statistical data analysis

Unless otherwise stated, the data presented herein were analyzed with: GraphPad Prism software was used for statistical analysis and data plotting; Proprietary Leica and Zeiss confocal software was used for IF imaging, while Fiji software (imageJ) was used for downstream analysis; BrainWave 5 software and custom codes developed in Python were used to analyze the raw recording data of neuronal activity on the HD-MEA.

SUPPLEMENTAL REFERENCES

Arshadi, C., Günther, U., Eddison, M., Harrington, K.I.S., and Ferreira, T.A. (2021). SNT: a unifying toolbox for quantification of neuronal anatomy. Nat Methods *18*, 374–377. https://doi.org/10.1038/s41592-021-01105-7.

Bertacchi, M., Pandolfini, L., D'Onofrio, M., Brandi, R., and Cremisi, F. (2015). The double inhibition of endogenously produced BMP and W nt factors synergistically triggers dorsal telencephalic differentiation of mouse ES cells. Developmental Neurobiology *75*, 66–79. https://doi.org/10.1002/dneu.22209.

Chao, Z.C., Bakkum, D.J., and Potter, S.M. (2007). Region-specific network plasticity in simulated and living cortical networks: comparison of the center of activity trajectory (CAT) with other statistics. J. Neural Eng. *4*, 294–308. https://doi.org/10.1088/1741-2560/4/3/015.

Chiappalone, M., Novellino, A., Vajda, I., Vato, A., Martinoia, S., and Van Pelt, J. (2005). Burst detection algorithms for the analysis of spatio-temporal patterns in cortical networks of neurons. Neurocomputing *65–66*, 653–662. https://doi.org/10.1016/j.neucom.2004.10.094.

Harris KM, Jensen FE, and Tsao B (1992). Three-dimensional structure of dendritic spines and synapses in rat hippocampus (CA1) at postnatal day 15 and adult ages: implications for the maturation of synaptic physiology and long-term potentiation. Journal of Neuroscience 12 (7) 2685-2705. https://doi.org/10.1523/JNEUROSCI.12-07-02685.1992.

Izhikevich, E.M. (2006). Polychronization: Computation with Spikes. Neural Computation *18*, 245–282. https://doi.org/10.1162/089976606775093882.

Kempf, J., Knelles, K., Hersbach, B.A., Petrik, D., Riedemann, T., Bednarova, V., Janjic, A., Simon-Ebert, T., Enard, W., Smialowski, P., et al. (2021). Heterogeneity of neurons reprogrammed from spinal cord astrocytes by the proneural factors Ascl1 and Neurogenin2. Cell Reports *36*, 109409. https://doi.org/10.1016/j.celrep.2021.109409.

Kuo, H.-Y., Yang, Y.-H., Chen, S.-Y., Kuo, T.-H., Lin, W.-T., and Liu, F.-C. (2023). Differential Development of Dendritic Spines in Striatal Projection Neurons of Direct and Indirect Pathways in the Caudoputamen and Nucleus Accumbens. eNeuro *10*, ENEURO.0366-22.2023. https://doi.org/10.1523/ENEURO.0366-22.2023.

Maccione, A., Gandolfo, M., Massobrio, P., Novellino, A., Martinoia, S., and Chiappalone, M. (2009). A novel algorithm for precise identification of spikes in extracellularly recorded neuronal signals. Journal of Neuroscience Methods *177*, 241–249. https://doi.org/10.1016/j.jneumeth.2008.09.026.

Miura, Y., Li, M.-Y., Birey, F., Ikeda, K., Revah, O., Thete, M.V., Park, J.-Y., Puno, A., Lee, S.H., Porteus, M.H., et al. (2020). Generation of human striatal organoids and cortico-striatal assembloids from human pluripotent stem cells. Nature Biotechnology *38*, 1421–1430. https://doi.org/10.1038/s41587-020-00763-w.

Parodi, G., Brofiga, M., Pastore, V.P., Chiappalone, M., and Martinoia, S. (2023). Deepening the role of excitation/inhibition balance in human iPSCs-derived neuronal networks coupled to MEAs during long-term development. J. Neural Eng. *20*, 056011. https://doi.org/10.1088/1741-2552/acf78b.

Pfaffl, M.W. (2001). A new mathematical model for relative quantification in real-time RT-PCR. Nucleic Acids Research *29*, 45e–445. https://doi.org/10.1093/nar/29.9.e45.

Poli, D., Pastore, V.P., and Massobrio, P. (2015). Functional connectivity in in vitro neuronal assemblies. Front. Neural Circuits *9*. https://doi.org/10.3389/fncir.2015.00057.

Silva, J., Barrandon, O., Nichols, J., Kawaguchi, J., Theunissen, T.W., and Smith, A. (2008). Promotion of Reprogramming to Ground State Pluripotency by Signal Inhibition. PLoS Biol *6*, e253. https://doi.org/10.1371/journal.pbio.0060253.

Tonelli, F., Iannello, L., Gustincich, S., Di Garbo, A., Pandolfini, L., and Cremisi, F. (2025). Dual inhibition of MAPK/ERK and BMP signaling induces entorhinal-like identity in mouse ESC-derived pallial progenitors. Stem Cell Reports 102387. https://doi.org/10.1016/j.stemcr.2024.12.002.

Ullo, S., Nieus, T.R., Sona, D., Maccione, A., Berdondini, L., and Murino, V. (2014). Functional connectivity estimation over large networks at cellular resolution based on electrophysiological recordings and structural prior. Front. Neuroanat. *8*. https://doi.org/10.3389/fnana.2014.00137.
